# Analyses of Transcriptomics upon IL-1β-Stimulated Mouse Chondrocytes and the Protective Effect of Catalpol through the NOD2/NF-κB/MAPK Signaling Pathway

**DOI:** 10.3390/molecules28041606

**Published:** 2023-02-07

**Authors:** Yong Pang, Lu Zhao, Xueyan Ji, Kaijin Guo, Xiaoxing Yin

**Affiliations:** 1 School of Basic Medical Sciences, Nanjing Medical University, Nanjing 211166, China; 2 Department of Orthopedics, The Affiliated Hospital of Xuzhou Medical University, Xuzhou 221004, China; 3 Jiangsu Key Laboratory of New Drug Research and Clinical Pharmacy, Xuzhou Medical University, Xuzhou 221004, China

**Keywords:** osteoarthritis (OA), catalpol, NOD2, MAPK

## Abstract

The overall objective of this study was to investigate the mechanism of inflammation on chondrocyte injury and the protective effect of catalpol on chondrocytes in an inflammatory environment. Chondrocytes were isolated and cultured from the knee joints of three-day-old newborn mice. Alcian Blue staining and the immunocytochemistry staining of type II collagen were used to identify the purity of chondrocytes. Primary chondrocytes were stimulated by IL-1β (10 ng/mL) and subjected to transcriptome analysis. Differentially expressed genes (DEGs) were further analyzed based on Gene Ontology (GO) and Kyoto Encyclopedia of Genes and Genomes (KEGG) pathway enrichment analyses. In this experimental study, we performed the viability assay to determine the effects of different concentrations of catalpol on the cell viability of chondrocytes. Chondrocytes were seeded in six-well plates and exposed to 10 μM catalpol 2 h prior to treatment with IL-1β (10 ng/mL). Quantitative real-time (qPCR) and Western blotting were performed to evaluate the RNA and protein expression, respectively. Based on the results of transcriptomics analysis, we found the NOD2 signaling pathway, the NF-kappa B signaling pathway, and the MAPK signaling pathway showed significant changes in chondrocyte damage caused by inflammation. Catalpol (10 μM and 100 μM) could significantly reduce NO, IL-6, IL-1β, and TNF-α in supernatant of chondrocytes. Catalpol significantly inhibited the mRNA expression of IL-1, IL-6, and IL-12 in chondrocytes induced by IL-1β. Catalpol markedly inhibited MMP3, MMP13 mRNA, and protein levels. Catalpol could significantly reduce TNF-α mRNA levels in inflammatory chondrocytes. Inflammation causes significant increases in mRNA levels and protein levels of NOD2, mRNA levels, and protein levels were markedly suppressed by catalpol. In addition, catalpol could significantly increase IKBα protein levels and significantly lower intranuclear P65 levels. Catalpol significantly lowered the phosphorylation protein levels of ERK, p38, and JNK. Our transcriptomic analysis demonstrated that the activation of NOD2 and its downstream pathways, NF-κB and MAPK, is an important cause of the inflammatory injury to chondrocytes induced by IL-1β. Catalpol inhibited the activation of the NOD2 signaling pathway, which reduced the phosphorylation of ERK, p38, and JNK, inhibited the degradation of IκBα, inhibited p65 translocation into the nucleus, reduced the release of inflammatory cytokines, and attenuated the inflammatory damage to chondrocytes.

## 1. Introduction

Osteoarthritis (OA), a chronic progressive joint disease, represents a significant socioeconomic and public health burden [1]. Currently, the pathogenesis of OA remains largely unknown, and pharmacotherapies relieve OA symptoms temporarily but fail to prevent or slow down the disease’s progression. It is urgent to discover effective therapeutic targets and effective therapeutic methods to provide clinical benefits for patients with OA [2]. As cartilage degradation induced by chondrocyte inflammation is the major determining factor for the development of pathogenesis, chondrocyte hypertrophy and apoptosis have been proposed as mechanisms of OA initiation [3]. Thus, the prevention of chondrocyte cell death and inflammation may contribute to the discovery of new therapeutic drugs and targets for OA treatment.

NOD2, one of 22 different cytosolic pattern recognition receptors known as NOD-like receptors (NLRs), acts as an intracellular sensor for molecules that contain a specific structure called muramyl dipeptide (MDP), a peptidoglycan constituent of both Gram-positive and Gram-negative bacteria [4,5]. After stimulation, NOD2 recruits receptor-interacting serine/threonine-protein kinase 2 (RIP2), resulting in activation of the transcription factors NF-κB and mitogen-activated protein kinase (MAPK), which, in turn, promotes the expression of numerous genes involved in the inflammatory process expression [6]. NF-κB and MAPK, two major pathways involved in OA development, are involved in the degradation of articular cartilage in OA development [7]. In addition, the chondroprotective effects of many compounds are mediated by inhibiting the activation of NF-κB and MAPK signaling.

*Rehmannia glutinosa* has been widely used in Chinese medicine for the treatment of bone diseases, including osteoporosis [8,9,10] and osteoarthritis [11]. Catalpol, an iridoid glucoside, is the major active ingredient of *Rehmannia glutinosa*. Numerous studies have proven that catalpol has a variety of biological activities, including neuroprotective, hypoglycemic, anticancer, and anti-inflammatory effects. In addition, there is a study showing that catalpol treatment can prevent IL-1ß-induced chondrocyte injury through inhibiting the NF-κB pathway [12]. However, the protective mechanisms on chondrocytes underlying its anti-inflammatory effects are still poorly understood. In order to further investigate the mechanisms of chondrocyte inflammatory damage in OA pathogenesis, we decided to use transcriptomics to study the underlying mechanisms. Moreover, based on the obtained results of transcriptomics, we explore the protection mechanism of catalpol on chondrocyte damage caused by inflammation.

## 2. Results

### 2.1. Identification of Chondrocytes and Inflammatory Model Conditions

**Figure 1:**
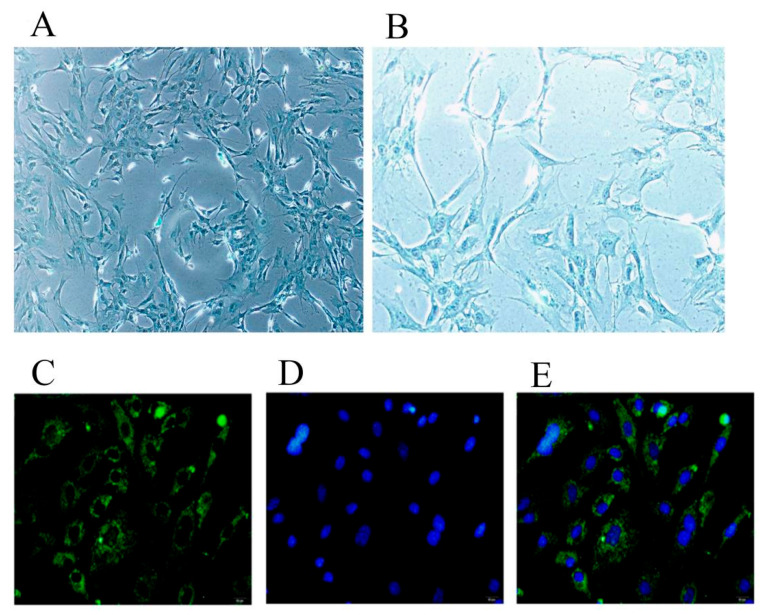
Identification of the purity of chondrocytes. (**A**) Chondrocytes were identified by Alcian Blue staining (magnification, ×100). (**B**) Chondrocytes were identified by Alcian Blue staining (magnification, ×200). (**C**–**E**) Chondrocytes were identified by the immunocytochemistry staining of type II collagen. (**C**) Type II collagen; (**D**) DAPI; and (**E**) the merged image (magnification, ×200).

**Figure 2:**
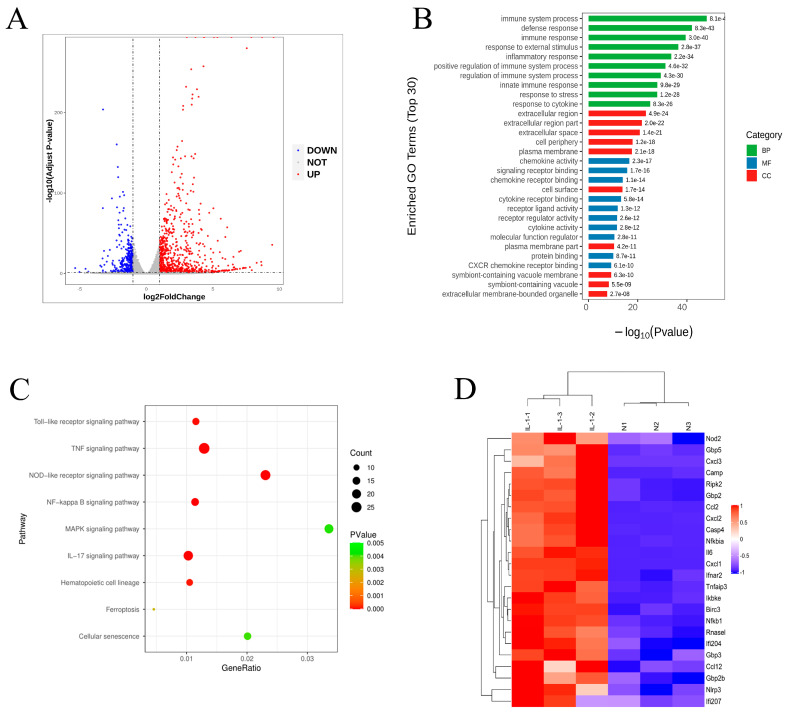
Transcriptome analysis of inflammation induced by IL-1β on the functionality of chondrocytes. (**A**) Volcano plot showing comparative gene expression in normal chondrocytes and chondrocytes induced by IL-1β (n = 3). Red dots indicated the significantly increased transcripts (870), and blue dots represented the significantly decreased transcripts (368) in inflammatory chondrocytes induced by IL-1β (|log 2 FC| > 1, *padj* < 0.05). (**B**) GO enrichment analysis for differential gene expression in normal chondrocytes and inflammatory chondrocytes. GO enrichment analyses were performed for each of the three GO categories: BP, MF, and CC. (**C**) KEGG pathway analysis of differential gene expression in normal chondrocytes and inflammatory chondrocytes. (**D**) A heat map showing hierarchical clustering of differential gene expression along the NOD2 signaling pathway in normal and inflammatory chondrocytes. Red indicated high relative expression, and blue represented low relative expression.

As shown in Figure 1, the cells were identified as chondrocytes by Alcian Blue staining and immunofluorescence of type II collagen staining. As showcased in Figure 2, IL-1β can induce a stable inflammatory state of chondrocytes at a concentration greater than or equal to 10 ng/mL for 36–72 h. In subsequent experiments, we used 10 ng/mL IL-1β to treat chondrocytes for 48 h as the condition for establishing an inflammatory injury model in transcriptomic experiments and experiments of the effects of catalpol on chondrocytes.

### 2.2. Signaling Pathway Prediction Based on GO and KEGG Analyses

To gain further insight into the molecular basis for inflammation action on chondrocytes, we conducted GO and KEGG analyses. A volcano plot shows comparative gene expression in normal chondrocytes and chondrocytes induced by IL-1β (n = 3) (Figure 2A). Red dots indicated the significantly increased transcripts (870), and blue dots represented the significantly decreased transcripts (368) in inflammatory chondrocytes induced by IL-1β (|log 2 FC| > 1, *padj* < 0.05). We conducted GO analyses for the three GO domains: BP, CC, and MF. Immune system processes, defense responses, inflammatory responses, responses to external stimuli, and regulation of multicellular organismal processes differ significantly from the control group (Figure 2B). KEGG analysis was performed as described previously using the KEGG database [13]. Through transcriptomic KEGG enrichment analysis, the main differential pathways of IL-1β induced inflammatory injury in mouse chondrocytes were found in this study, including the TNF signaling pathway, cytokine-cytokine receptor interaction, IL-17 signaling pathway, NOD-like receptor signaling pathway, chemokine signaling pathway, NF-kappa B signaling pathway, toll-like receptor signaling pathway, cell adhesion molecules, hematopoietic cell lineage, calcium signaling pathway, MAPK signaling pathway, and cellular senescence (Figure 2C). In addition, heat map analysis of differentially expressed genes showed hierarchical clustering of differential gene expression along the NOD2 signaling pathway between two groups (Figure 2D).

### 2.3. Effect of Catalpol on Cell Viability of Chondrocytes

**Figure 3:**
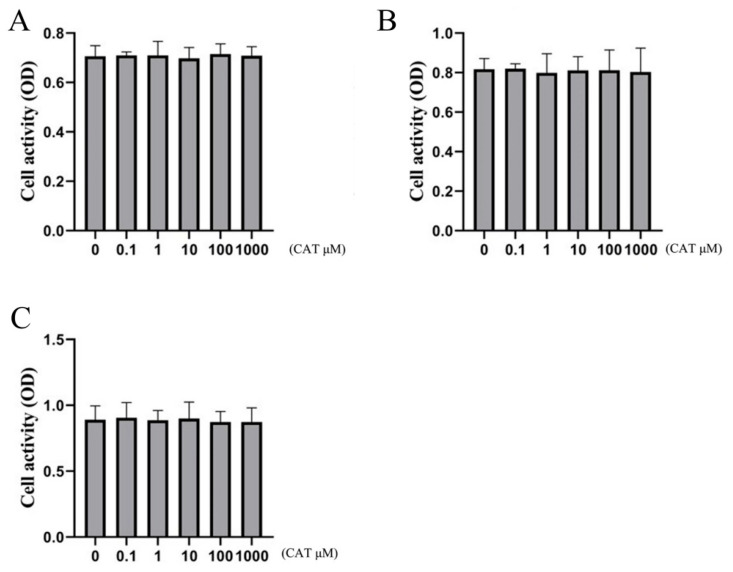
Effect of catalpol on the cell viability of chondrocytes. To confirm that catalpol was not itself cytotoxic to the chondrocytes, we used the CCK-8 assay to estimate the potential cytotoxic effect of catalpol. (**A**) 24 h, (**B**) 48 h, and (**C**) 72 h. The data are the mean ± S.D. of the mean (n = 10).

To confirm that catalpol itself was not cytotoxic up to the highest concentration tested, 1000 mM, we used the CCK-8 assay to estimate the potential cytotoxic effect of catalpol. These results can be seen in Figure 3. The CCK-8 assay indicated that catalpol did not have cytotoxicity within a concentration range of 1000 μM.

### 2.4. Catalpol Inhibits IL-1β-Induced Inflammatory Cytokine Release

**Figure 4:**
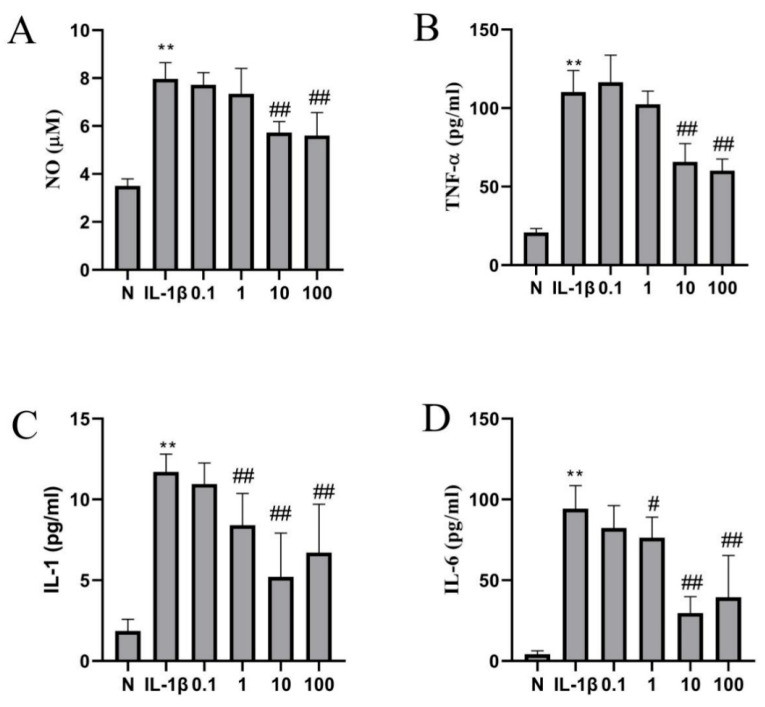
The effect of catalpol on the secretion of nitric oxide and inflammatory cytokines of chondrocytes induced by IL-1β. (**A**) NO, (**B**) TNF-α, (**C**) IL-1, (**D**) IL-6. The data are the mean ± S.D. of the mean (n = 6). ** *p* < 0.01 vs. normal group (N); # *p* < 0.05 vs. IL-1β group; ## *p* < 0.01 vs. IL-1β group.

We tested the effects of 0.1 μM, 1 μM, 10 μM, and 100 μM catalpol on NO levels in an inflammatory cell model induced by 10 ng/mL IL-1β. As shown in Figure 4A, the NO level in the supernatant of chondrocytes was significantly decreased in 10 μM (*p* < 0.01) and 100 μM (*p* < 0.01) catalpol groups onward trend compared to the IL-1β group was. Although there was no significant difference in the groups of 0.1 and 1 μM catalpol, a doobserved.

In addition, catalpol markedly decreased levels of IL-6, IL-1β, and TNF-α in chondrocytes. As shown in Figure 4B–D, the levels of IL-6 (*p* < 0.01), IL-1β (*p* < 0.01), and TNF-α (*p* < 0.01) in the supernatant of chondrocytes in the model group were significantly increased after IL-1β (10 ng/mL) treatment. An amount of 10 μM (*p* < 0.01) catalpol and 100 μM (*p* < 0.01) catalpol significantly attenuates TNF-α levels evoked by IL-1β (10 ng/mL) in the supernatant of chondrocytes. An amount of 1μM catalpol (*p* < 0.05), 10 μM (*p* < 0.01) catalpol, and 100 μ M catalpol (*p* < 0.01) significantly lowered the IL-1 and IL-6 levels in chondrocytes evoked by IL-1β (10 ng/mL)-induced inflammation. Referring to these results and the result of the CCK-8 assay, we chose 10 μM of catalpol in the subsequent experiment.

### 2.5. The Effects of Catalpol on the Levels of Inflammasome Activation-Associated Proteins and mRNA

**Figure 5:**
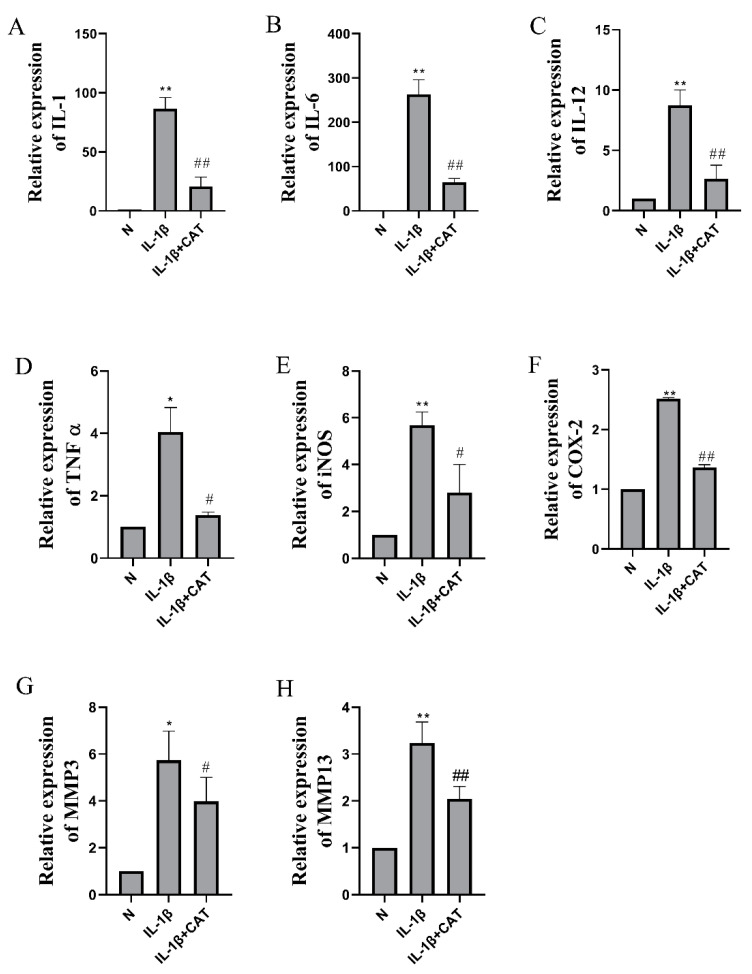
The effects of catalpol on the levels of inflammatory-related mRNA. (**A**) IL-1, (**B**) IL-6, (**C**) IL-12, (**D**) TNF-α, (**E**) iNOS, (**F**) COX-2, (**G**) MMP3, (**H**) MMP13. Data are mean ± S.D. of the mean (n = 3). * *p* < 0.05 vs. normal group (N); ** *p* < 0.01 vs. normal group (N); # *p* < 0.05 vs. IL-1β group; ## *p* < 0.01 vs. IL-1β group.

**Figure 6:**
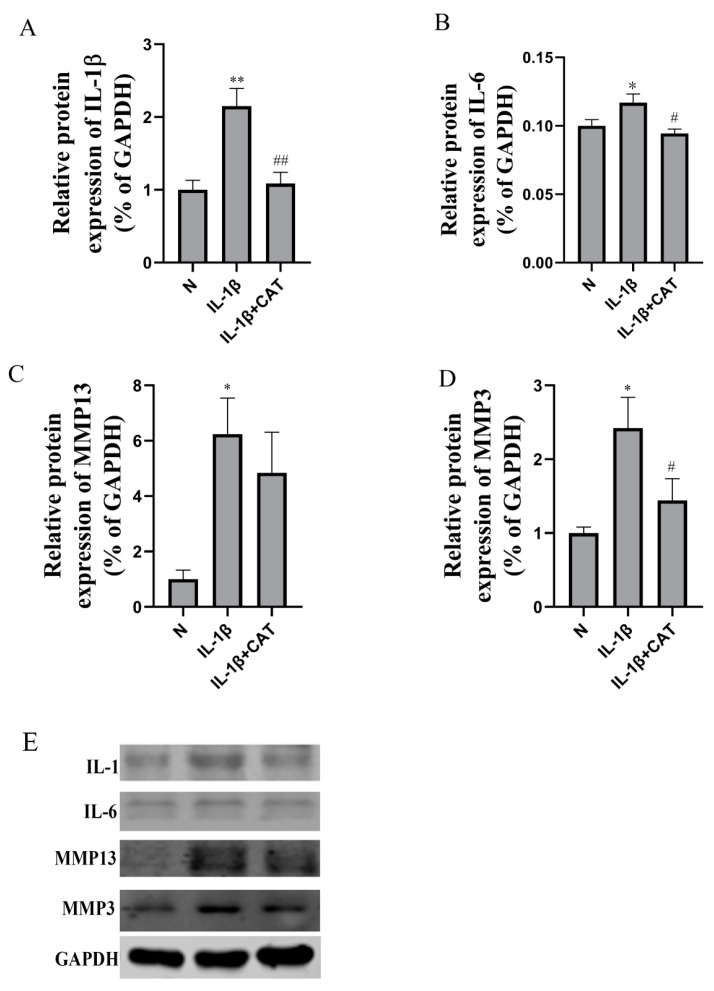
Western blot analysis of inflammasome activation-associated proteins expression in chondrocytes. (**A**) IL-1 protein level; (**B**) IL-6 protein level; (**C**) caspase-3 protein level; (**D**) MMP13 protein level; (**E**) MMP3 protein level. Above, densitometric analysis; below, representative images of immunoblots. The data are the mean ± S.D. of the mean (n = 3). * *p <* 0.05 vs. normal group (N); ** *p* < 0.01 vs. normal group (N); # *p* < 0.05 vs. IL-1β group; ## *p* < 0.01 vs. IL-1β group.

As shown in Figure 5, the qPCR results confirmed that inflammatory mRNA cytokine (IL-1, IL-6, and IL-12) levels were increased after IL-1β induction (*p* < 0.01). Catalpol significantly inhibited the mRNA expression of IL-1, IL-6, and IL-12 in chondrocytes induced by IL-1β. These results were consistent with the results of our previously mentioned ELISA analysis. The above results proved that catalpol possessed a favorable anti-inflammatory effect on chondrocytes induced by IL-1β. As shown in Figure 6, significant increases were also observed in the protein levels of IL-1β (*p* < 0.01) and IL-6 (*p* < 0.05) in the model group. IL-1β (*p* < 0.01) and IL-6 (*p* < 0.05) protein expression was markedly suppressed by catalpol.

In addition, the mRNA level of TNF-α in mouse chondrocytes after IL-1β (10 ng/mL) treatment was significantly increased (*p* < 0.05), and catalpol could significantly inhibit TNF-α mRNA level in inflammatory chondrocytes (*p* < 0.05) (Figure 5). IL-1β induces elevated levels of iNOS and COX-2 in mouse chondrocytes, and the upward trend of iNOS (*p* < 0.05) and COX-2 can be significantly restrained by catalpol. To investigate whether catalpol induced MMP3 and MMP13 downregulation in chondrocytes, we performed Western blotting and RT-PCR to detect MMP3 and MMP13 protein and mRNA expression, respectively. The results indicated that IL-1β can significantly upregulate MMP3 and MMP13, and catalpol markedly inhibited MMP3 and MMP13 mRNA (Figure 5) and protein levels (Figure 6).

### 2.6. Catalpol Inhibits Activation of the NOD2-NF-κB/MAPK Pathway

**Figure 7:**
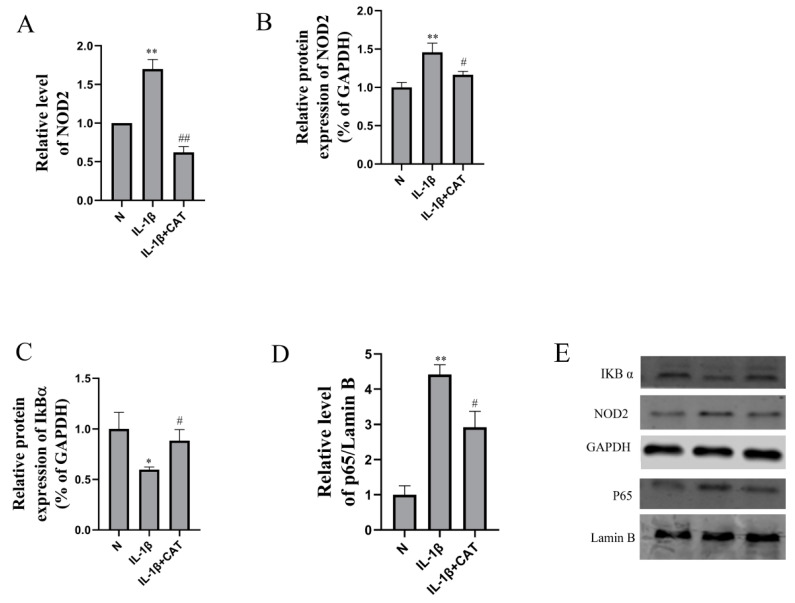
The effects of catalpol on the levels of NOD2, IKBα, and P65. (**A**) NOD2 mRNA level (n = 6); (**B**) NDD2 protein level; (**C**) IKBα protein level; (**D**) P65 protein level (**B**–**D** n = 3); above, densitometric analysis; below, (**E**) representative images of immunoblots. The data are the mean ± S.D. of the mean. * *p* < 0.05 vs. normal group (N); ** *p* < 0.01 vs. normal group (N); # *p* < 0.05 vs. IL-1β group; ## *p* < 0.01 vs. IL-1β group.

**Figure 8:**
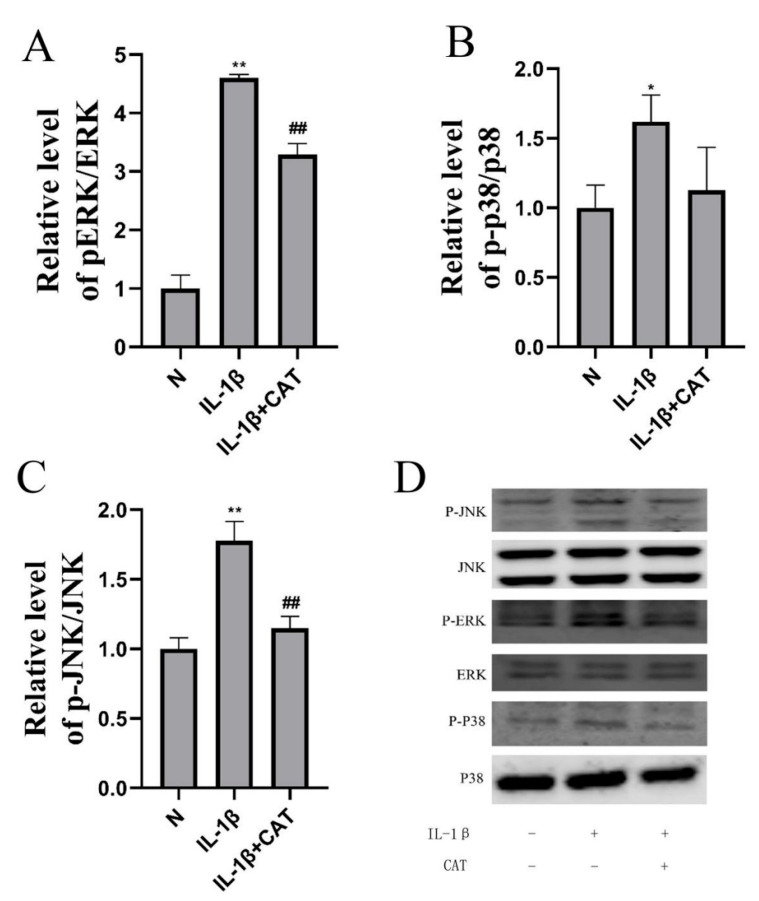
Catalpol inhibits activation of the MAPK pathway. (**A**) pERK/ERK level; (**B**) p-p38/p38 level; (**C**) p-JNK/JNK level; (**D**) representative images of immunoblots. The data are the mean ± S.D. of the mean (n = 3). * *p* < 0.05 vs. normal group (N); ** *p* < 0.01 vs. normal group (N); ## *p* < 0.01 vs. IL-1β group.

Western blotting was used to determine the levels of NOD2, MAPK, and NF-κB pathway related proteins. Inflammation causes significant increases in mRNA levels (Figure 7A) (*p* < 0.01) and protein levels (Figure 7B) (*p* < 0.01) of NOD2, and mRNA levels (Figure 7A) (*p* < 0.01) and protein levels (Figure 7B) (*p* < 0.05) were markedly suppressed by catalpol. In addition, IL-1β decreases IKBα protein level (*p* < 0.05), and the protein levels were increased in the catalpol-treated group (*p* < 0.05). In addition, P65 nuclear translocation was evaluated by IL-1β, and catalpol significantly lowered the intranuclear P65 level (Figure 7B, *p* < 0.05). The mitogen-activated protein kinase (MAPK) pathway, which includes MEK-ERK1/2, JNK, and p38 MAPK, is involved in chondrocyte proliferation and hypertrophy [14]. As shown in Figure 8, the protein phosphorylation of ERK, p38, and JNK was calculated as the ratio of phosphorylated-to-total protein expression. Inflammation caused significant increases in pERK/ERK level (*p* < 0.01), p-p38/p38 level (*p* < 0.05), and p-JNK/JNK level (*p* < 0.01), and catalpol significantly lowered the phosphorylation protein levels of ERK, p38, and JNK.

## 3. Discussion

In the current study, we initially found that catalpol, the main active ingredient in the Chinese traditional medicine *R. glutinosa*, can attenuate inflammatory responses in chondrocytes by inhibiting the hyperactivation of the NOD2-MAPK signaling pathway. Catalpol can reduce the mRNA expression and protein levels of inflammation-related factors including IL-1, IL-6, IL-12, TNF-α, iNOS, COX-2, MMP3, and MMP13 in an IL-1β-induced inflammatory injury model of chondrocytes. We found in the present study that catalpol reduced the level of NOD2 protein and inhibited the degradation of IKBα in chondrocytes, as well as the phosphorylation of JNK, P38, and ERK. In summary, all the above results suggest that catalpol protects against inflammatory injury in chondrocytes by inhibiting hyperactivation of NOD2 and its downstream pathway.

In the transcriptomics experiment, we found that the hyperactivation of NOD2 and its downstream pathways, NF-κB and MAPK, play important roles in the inflammation of chondrocytes. NOD2, a kind of cytosolic receptor such as nucleotide-binding oligomerization domain (NOD)-like receptors, mediates cytokine responses by interacting with the CARD-containing kinase RIP2 to activate MAPK and NF-κB signaling [15]. Despite extensive research on articular degenerative disorders, the mechanisms of these disorders remain to be determined. Although studies with a few clinical cases have found that NOD2/RIPK2 signaling is upregulated in immune cells of RA patients compared with OA patients [16], inhibition of the hyperactivation of the NOD2 pathway could be a new potential strategy for treatment and prevention of OA. In addition, a recent study found that PKR activation induced by TNF-α also plays an important role in the pathogenesis of osteoarthritis, triggering oxidative stress-mediated inflammation and MMP-13 in chondrocytes [17]. Catalpol may protect articular cartilage from degeneration through a variety of mechanisms, and the specific mechanisms require further studies.

Radix Rehmanniae Praeparatac, the steamed root of *R. glutinosa*, which is an important ingredient of diverse TCM formulas, has been used for various medicinal purposes in East Asia for thousands of years [18]. This traditional Chinese medicine exhibits a wide range of pharmacological activities with low toxicity and side effects. Catalpol has been demonstrated to possess a variety of activities such as alleviating diabetes mellitus and its complications [19], anti-atherosclerosis [20,21], anti-oxidation [22], anti-tumor [23], and anti-osteoporosis based on in vitro and in vivo pharmacodynamic experiments [24,25]. The present study found that catalpol protects against inflammatory injury in chondrocytes by inhibiting hyperactivation of NOD2 and its downstream pathway.

However, there are several limitations that need to be addressed in future studies. Although hyperactivation of NOD2 and its downstream pathway have been demonstrated to play an important role in inflammation-induced chondrocyte injury at the cellular level, this conclusion needs to be verified in future animal and clinical studies. In addition, a detailed mechanism for catalpol would need further investigation to become a candidate drug in the treatment of OA.

## 4. Materials and Methods

### 4.1. Reagents

Catalpol (99% purity) was obtained from Nanjing King Bamboo Biological Technology Co., Ltd. (Nanjing, China), and fetal bovine serum was purchased from Hyclone. The GAPDH (AP0063) was purchased from Bioworld (Jiangsu, China), IL-1 beta (26048-1-AP), IL-6 (66146-1-lg), MMP3 (17873-1-AP), MMP13 (18165-1-AP), IkB Alpha (10268-1-AP), Lamin B1 (12987-1-AP), and NF-κB p65 (10745-1-AP) were purchased from Proteintech (Wuhan, China), NOD2 (DF12125) was purchased from Affinity (Jiangsu, China), and JNK(ET1601-28), P-JNK(ET1609-42), ERK(ET1601-29), P-ERK(ET1610-13), P38(ET1602-26), and P-P38(ER2001-52) were purchased from HUABIO (Zhejiang, China), and all other chemicals were purchased from Sigma.

### 4.2. Culturing and Identification of Chondrocytes

All animal manipulations in this study were approved by the Animal Care and Use Committee of Xuzhou Medical University (ethical approval no. 202209S020). Chondrocytes were isolated and cultured as previously detailed [26]. In brief, cartilage was obtained from the knee joint of 3-day-old newborn mice obtained from the Laboratory Animal Center of Xuzhou Medical University under a stereo light microscope. Then, the cartilage was digested using 0.25% trypsinase and 0.1% collagenase II for 4 h to obtain cells. Chondrocytes were then washed with PBS and seeded in a cell culture flask with DMEM supplemented with 1% penicillin-streptomycin solution and 10% fetal bovine serum at a density of 5 × 10^5^ cells/cm^2^ at 37 °C with 5% CO_2_. The primary cells were used at passages 3–8.

We used Alcian Blue staining and the immunocytochemistry staining of type II collagen to identify the purity of chondrocytes. For Alcian Blue staining, cells were cultured in six-well plates at a density of 5.0 × 10^4^ cell/well and subsequently stained at 80% confluence. The cells were then fixed in 4% paraformaldehyde for 30 min before being washed three times with 1 × PBS. The cells were stained with Alcian Blue solution (0.1% Alcian Blue 8GX in 0.1 N HCl) for 30 min at room temperature. Following Alcian Blue staining, cells were rinsed for 3 min, three times, with PBS. The staining results were observed using a microscope.

Isolated chondrocytes were seeded onto the coverslips and stained at 80% confluence using the following procedure: the cells were fixed with 4% paraformaldehyde at 4 °C for 30 min and blocked with 10% bovine serum albumin for 30 min at room temperature. The slides were then incubated with a monoclonal primary antibody targeting type II collagen overnight at 4 °C, followed by an incubation with a secondary antibody for 30 min. Finally, images were captured using a microscope [27].

### 4.3. Establishment of an Inflammation Model

The cells that were passaged for 3–8 generations were used in subsequent experiments. Chondrocytes were cultured in 96-well plates and stimulated by 5 ng/mL, 10 ng/mL, 25 ng/mL, 50 ng/mL, and 100 ng/mL IL-1β, respectively. The levels of NO in the supernatant were detected from 12 to 72 h after addition using a Griess assay (Biyuntian, Shanghai, China, S0021S). First, a standard curve was created to correlate the linear relationship between NO concentration and absorbance spectra. Then, add 50 μL of supernatant to 96-well plates. In that order, 50 μL Griess Reagent I and 50 μL Griess Reagent II were added. The absorbance of each sample was measured at 540 nm with a microplate reader [28].

### 4.4. Transcriptome Analysis

To demonstrate the effect of inflammation induced by IL-1β on the functionality of chondrocytes, the cells were collected after being incubated on the specimens for 48 h. Transcriptome sequencing and functional annotation analysis Total RNA extraction, RNA library construction, and transcriptome sequencing were performed at Shanghai Zhongke New Life Biotechnology Co., Ltd (Shanghai, China). Quality control checks were performed to confirm sequencing saturation and gene mapping distribution. Values of fragments per kilobase of transcript per million mapped reads (FPKM) were applied to express relative gene abundance. Differentially expressed genes (DEGs) were further analyzed based on Gene Ontology (GO) and Kyoto Encyclopedia of Genes and Genomes (KEGG) pathway enrichment analyses [29] (Supplementary Materials).

### 4.5. Effect of Catalpol on Chondrocyte Viability

Cell Counting Kit-8 (CCK-8) (Biyuntian, China; C0038) was used to assess the effects of catalpol on chondrocyte viability. Chondrocytes were seeded in a 96-well plate at a density of 5 × 10^3^ cells per well in 100 µL of DMEM/F-12 medium. After cell attachment, the cultured cells were then pretreated with increasing concentrations (0 μM, 0.1 μM, 1 μM, 10 μM, 100 μM, and 1000 μM) of catalpol for 24 h, 48 h, and 72 h. For each well, 10 µL CCK-8 solution was added and incubated for 2 h at 37 °C. Then the cell density was calculated by a spectrophotometer at 450 nm. All experiments were performed six times.

### 4.6. Q-PCR

Chondrocytes were seeded in 6-well plates and exposed to 10 μM catalpol 2 h prior to treatment with IL-1β (10 ng/mL). After 48 h, the cells were harvested for the follow-up experiments. The methods and parameters of the quantitative polymerase chain reaction (qPCR) assay were performed as previously described [26]. Total RNA was extracted from collected cells using Trizol reagent (cat#: 9109; Invitrogen, Carlsbad, CA, USA) and was reverse transcribed into cDNA with the PrimeScript™ RT Reagent Kit (cat#: RR037A; TAKARA BIO INC, Shiga, Japan) according to the manufacturer’s instructions. The primer sequences used for qPCR are summarized in Table 1. Cycling conditions were 95 °C for 10 min, followed by 40 cycles of 95 °C for 15 s and 60 °C for 1 min. The q-PCR analysis was carried out 3 times. The relative graphs and statistical analyses of transcript quantities were calculated using the 2-ΔΔCt method with glyceraldehyde-3-phosphate dehydrogenase (GAPDH) as the endogenous reference gene amplified from the samples.

### 4.7. ELISA and the Griess Reaction

The concentration of NO was determined by the Griess reaction. The levels of inflammatory factors in cell supernatants were measured by ELISA according to the instructions of the ELISA kit (Biyuntian, China). ELISA was performed to detect the secreted concentrations of IL-6, IL-1β, and TNF-α using specific ELISA assay kits. The samples were added to 96-well plates pre-coated with antibodies and incubated. After washing with PBS-Tween, secondary goat anti-rabbit IgG-horseradish peroxidase (HRP) was added to the plates and incubated for 1 h at 37 °C, after which chromogen solution was poured into each well. The optical density was spectrophotometrically measured at 450 nm using a microplate reader in 30 min after 100 μL of stop buffer was added [30].

### 4.8. Western Blotting

In brief, cells were collected and lysed using RIPA buffer with 1% phenylmethanesulfonyl fluoride (PMSF), and then the protein concentration was quantified using the BCA Kit (Jiancheng, Nanjing, China). Then, equal amounts of 20 μg were separated and transferred using sodium dodecyl sulfate–polyacrylamide gel electrophoresis (SDS-PAGE) and a polyvinylidene fluoride (PVDF) membrane, followed by 1 h of sealing with 5% skimmed milk. Then, the primary antibodies in blocking buffer were added for overnight incubation at 4 °C. The excess primary antibody was washed off with Tris-buffered saline and 0.1% Tween-20 (TBST) three times for 5 min, followed by incubation with the secondary antibody at room temperature for 1 h. After being rinsed by TBST three times for 5 min, the band density was quantified using ImageJ software (LI-COR Biosciences) [31].

### 4.9. Statistical Analyses

Data were analyzed by SPSS and presented as mean ± SD. Every experiment has been repeated at least three times. Graphs were drawn using the GraphPad Prism (version 6.0 for Windows). Values with *p* < 0.05 were considered statistically significant, and *p* < 0.01 extremely significant.

## Figures and Tables

**Table 1:** Primer sequences in qRT-PCR.

Oligo Name	Sequence (5′–3′)
IL-1	F: CTG CAC TAC AGG CTC CGA
	R: GCC ACA GGT ATT TTG TCG TT
IL-6	F: TTA GCC ACT CCT TCT GTG ACT CC
	R: ACC CCA ATT TCC AAT GCT CT
IL-12a	F: GACCTGTTTACCACTGGAACTA
	R: GATCTGCTGATGGTTGTGATTC
TNF-α	F: TCG TAT GAA ATG GCA AAT CG
	R: GGT CCC AAC AAG GAG GAG
INOS	F: ATC CCG AAA CGA TAC ACT T
	R: TCT GGC GAA GAA CAA TCC
COX2	F: TCT ACA AGA CGC CAC ATC CC
	R: ACG GGG TTG TTG ATT TCG TCT
MMP3	F: GGA GGC AGC AGA GAA CCT AC
	R: TCC AAC CCG AGG AAC TTC TG
MMP13	F: CAG TGC TGC GGT TCA CTT TG
	R: TCA TCA TAA CTC CAC ACG TGG TT

## Data Availability

The data presented in this study are available in Supplementary Materials.

## Supplementary Materials

The following supporting information can be downloaded at: https://www.mdpi.com/article/10.3390/molecules28041606/s1, Differentially expressed genes (DEGs) analyzed data based on Gene Ontology (GO) and Kyoto Encyclopedia of Genes and Genomes (KEGG) pathway enrichment are provided.
