# Supplementary material for: Analyses of Transcriptomics upon IL-1β-Stimulated Mouse Chondrocytes and the Protective Effect of Catalpol through the NOD2/NF-κB/MAPK Signaling Pathway
Source: Molecules. 2023 Feb 7;28(4):1606. doi: 10.3390/molecules28041606 (PMC9962284; doi:10.3390/molecules28041606)
Supplement: Supplementary file 1 [file molecules-28-01606-s001.zip › raw data/KEGG/IL-1b_vs_N/DEG_pathway/mmu00900.html]

mmu00900.png


Close
