# Supplementary figures and images for: Analyses of Transcriptomics upon IL-1β-Stimulated Mouse Chondrocytes and the Protective Effect of Catalpol through the NOD2/NF-κB/MAPK Signaling Pathway (part 1 of 2)
Source: Molecules. 2023 Feb 7;28(4):1606. doi: 10.3390/molecules28041606 (PMC9962284; doi:10.3390/molecules28041606)

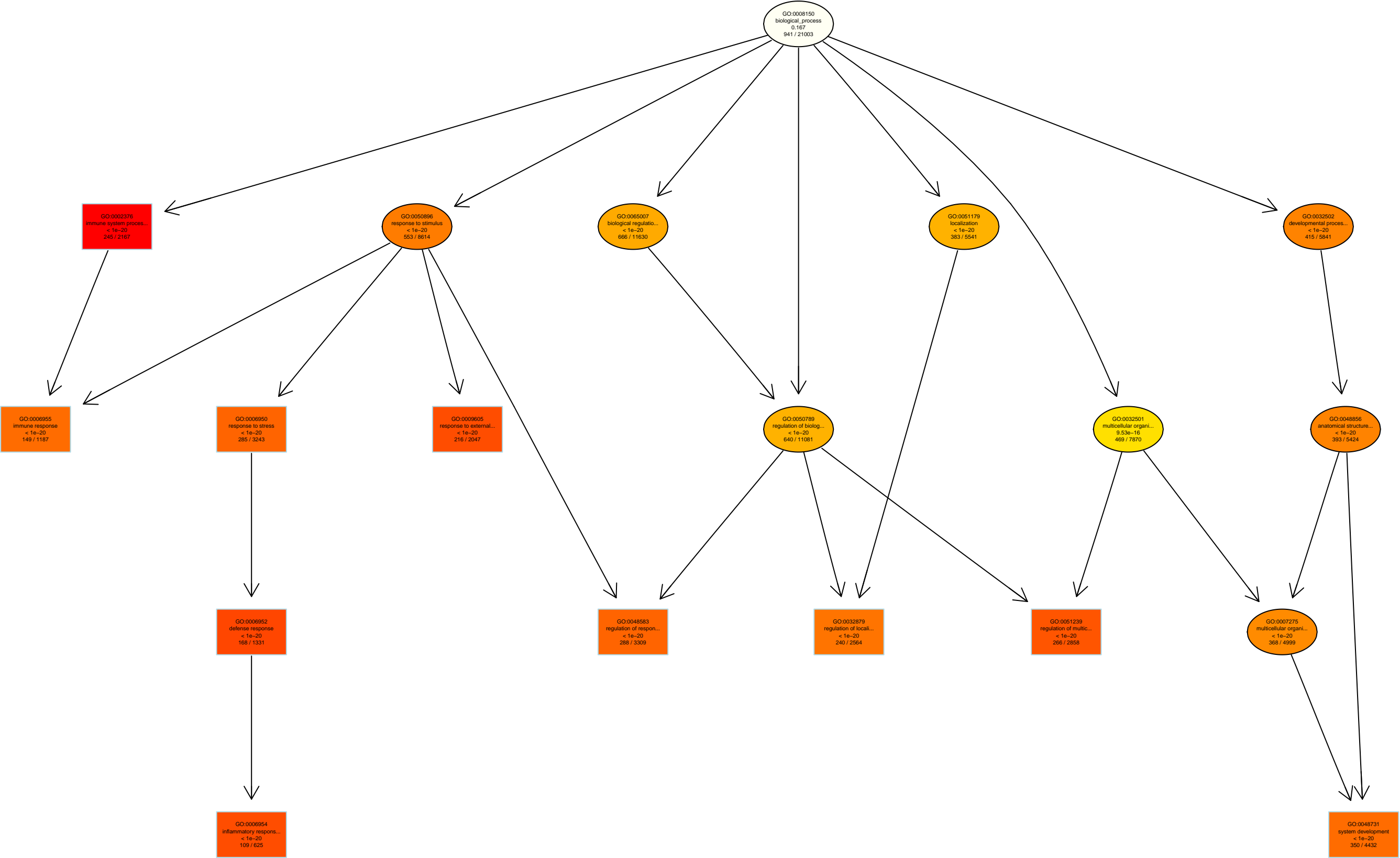

Supplement: Supplementary file 1 [file molecules-28-01606-s001.zip › raw data/GO/IL-1b_vs_N/DAG/IL-1b_vs_N.DEG_bp_DAG.pdf]

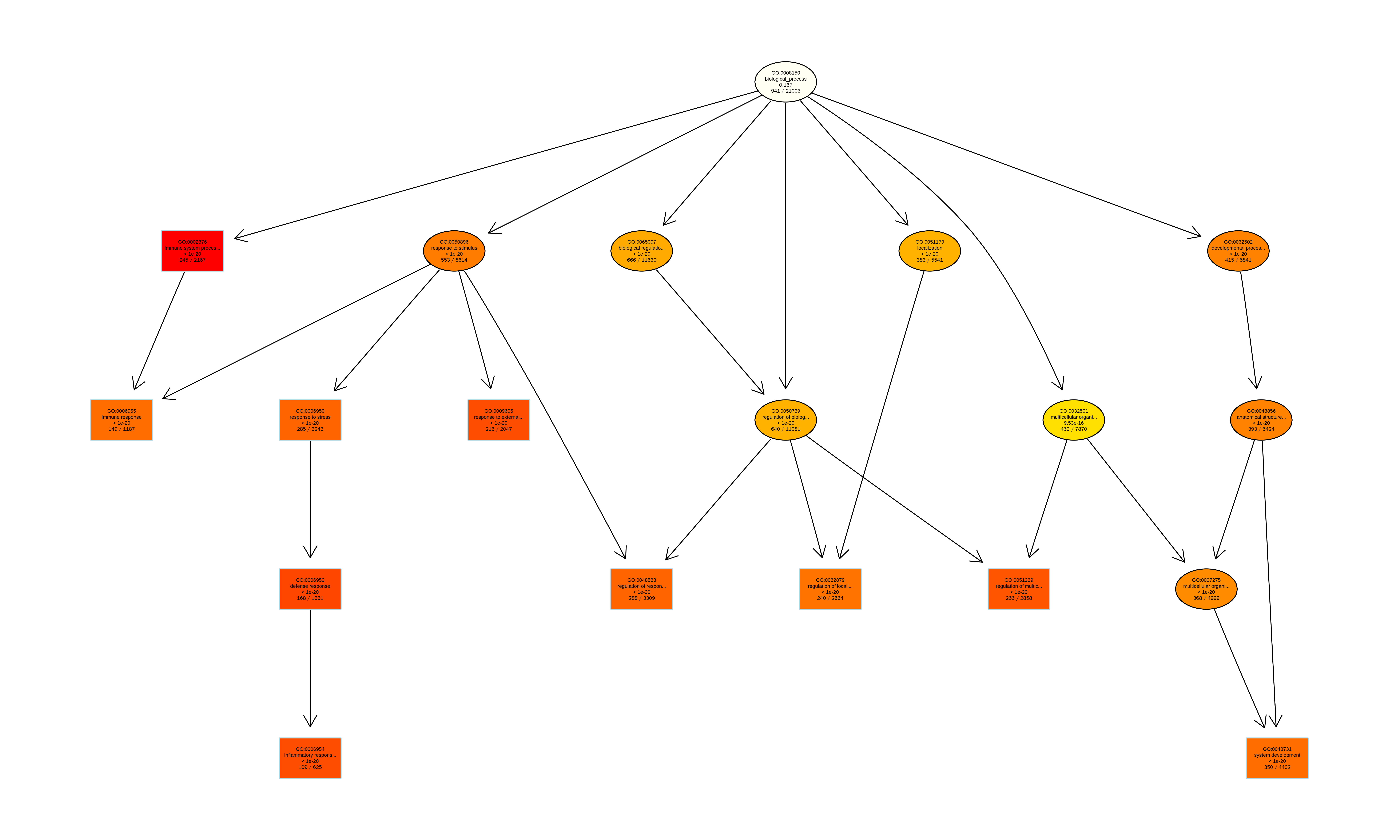

Supplement: Supplementary file 1 [file molecules-28-01606-s001.zip › raw data/GO/IL-1b_vs_N/DAG/IL-1b_vs_N.DEG_bp_DAG.png]

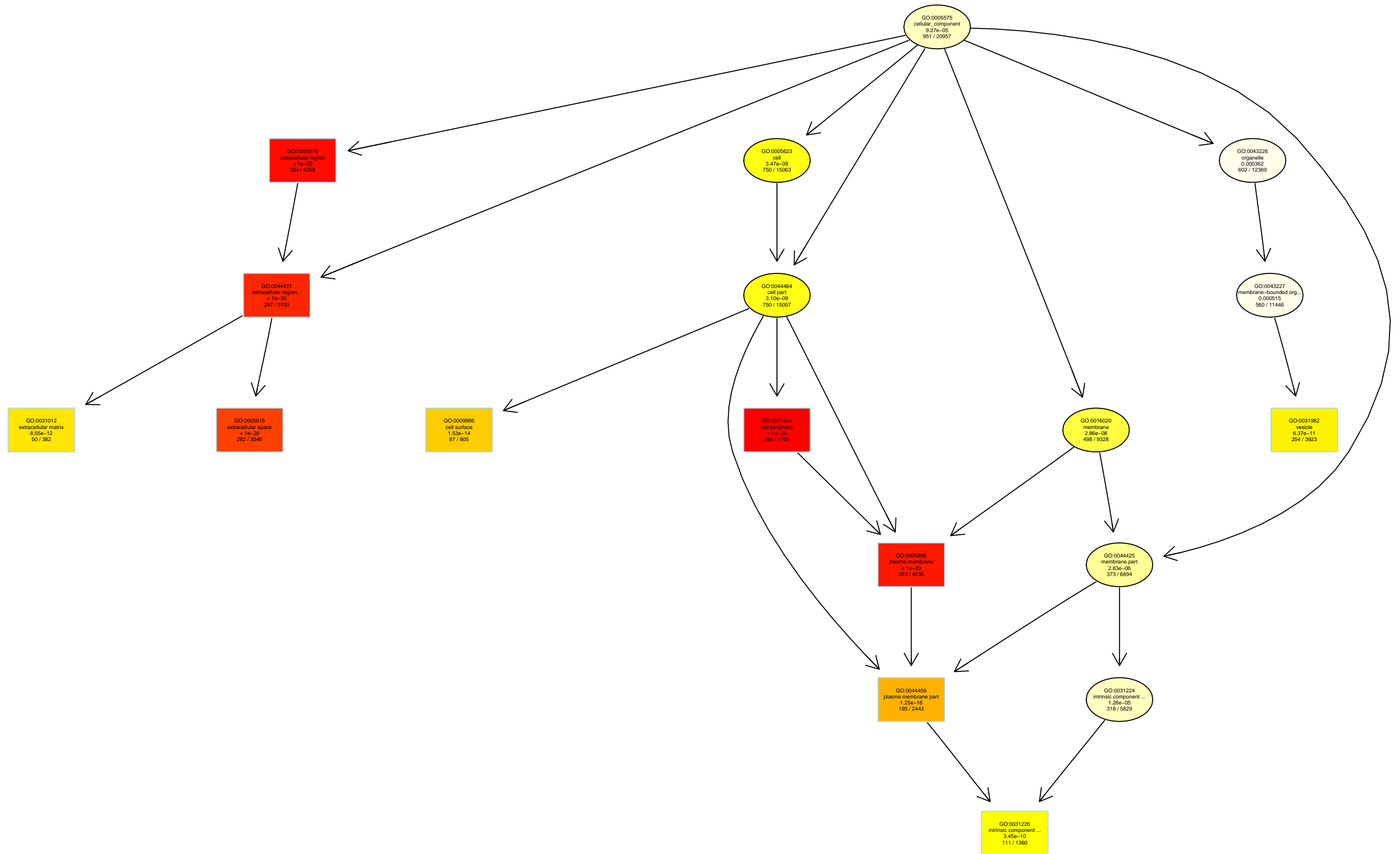

Supplement: Supplementary file 1 [file molecules-28-01606-s001.zip › raw data/GO/IL-1b_vs_N/DAG/IL-1b_vs_N.DEG_cc_DAG.pdf]

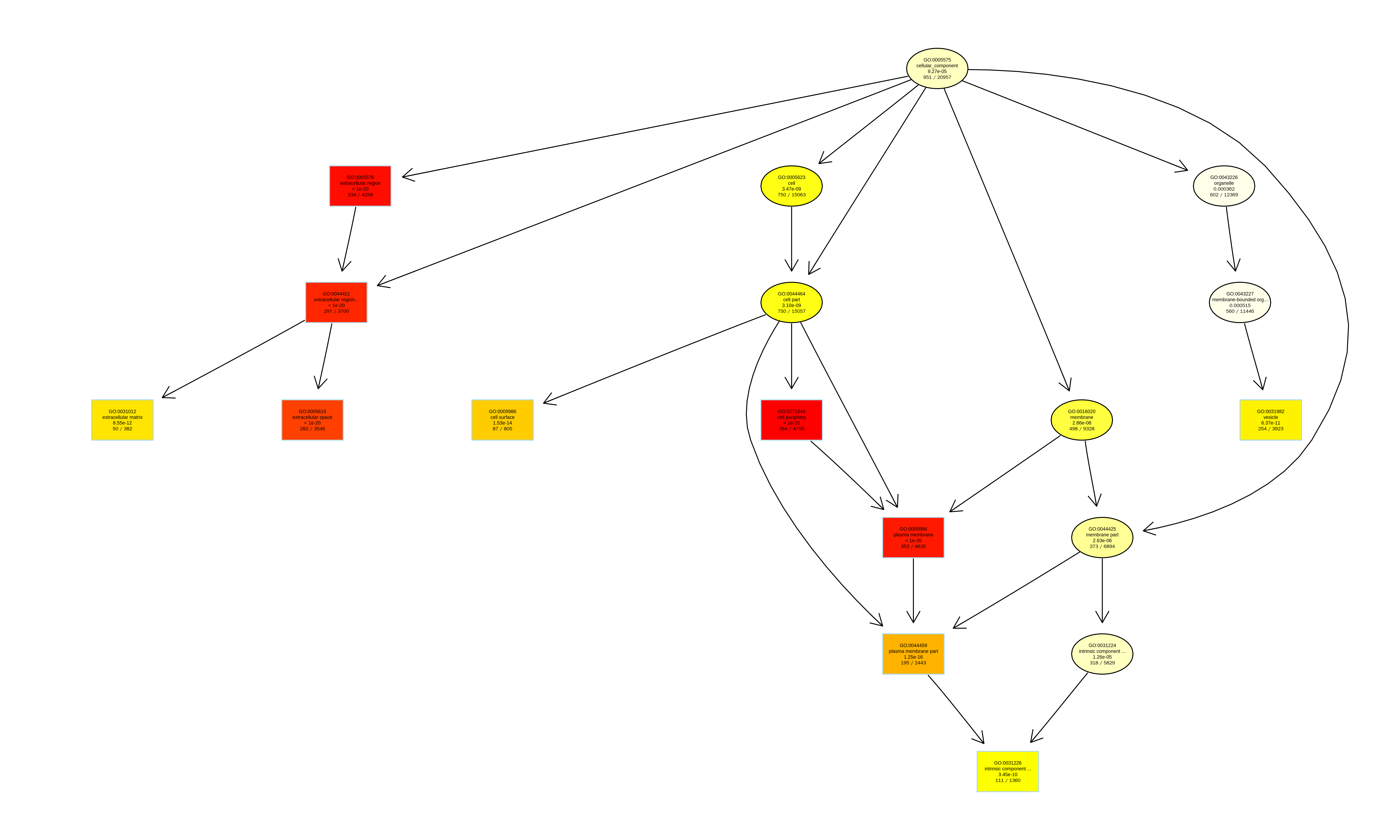

Supplement: Supplementary file 1 [file molecules-28-01606-s001.zip › raw data/GO/IL-1b_vs_N/DAG/IL-1b_vs_N.DEG_cc_DAG.png]

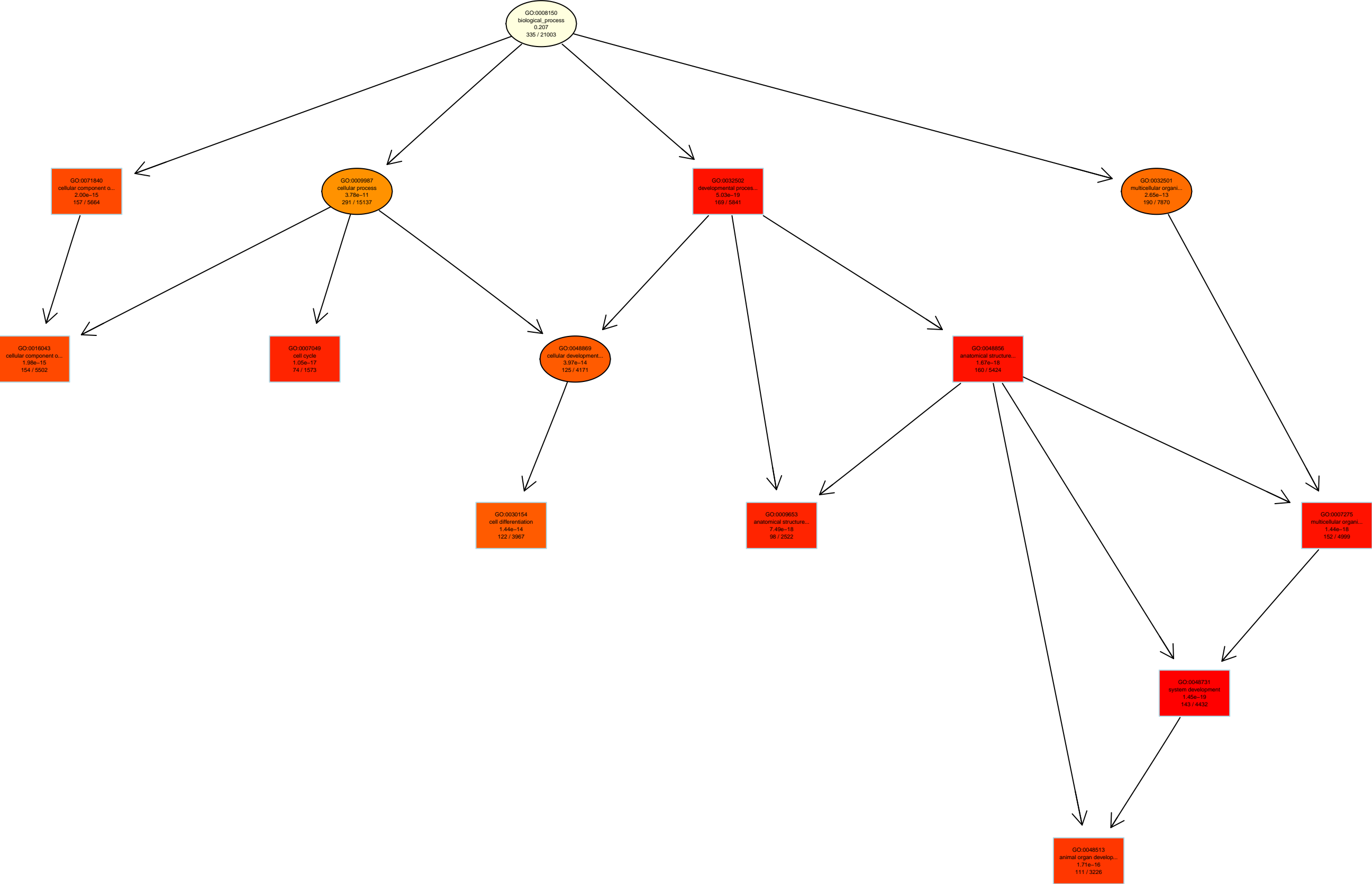

Supplement: Supplementary file 1 [file molecules-28-01606-s001.zip › raw data/GO/IL-1b_vs_N/DAG/IL-1b_vs_N.DEG_down_bp_DAG.pdf]

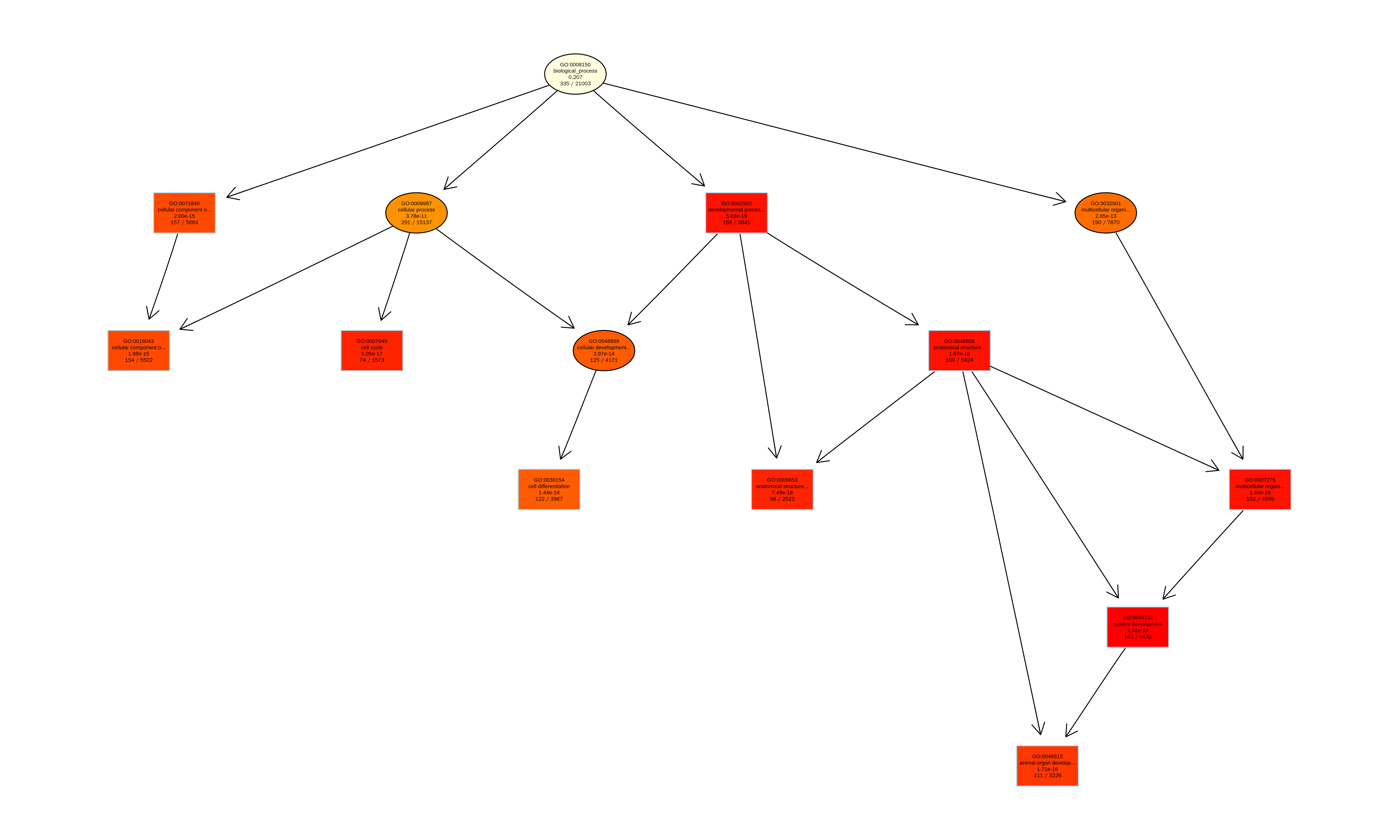

Supplement: Supplementary file 1 [file molecules-28-01606-s001.zip › raw data/GO/IL-1b_vs_N/DAG/IL-1b_vs_N.DEG_down_bp_DAG.png]

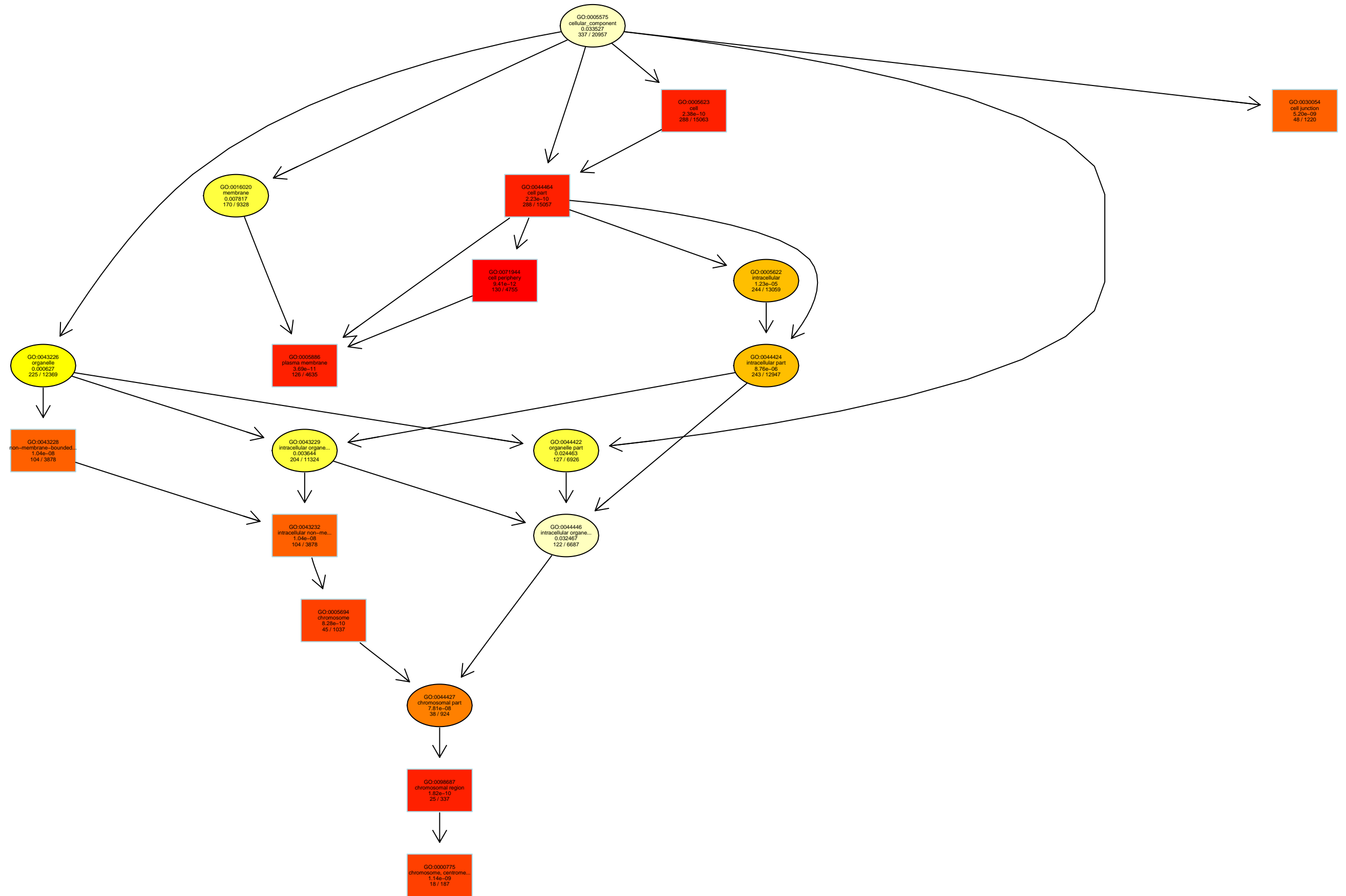

Supplement: Supplementary file 1 [file molecules-28-01606-s001.zip › raw data/GO/IL-1b_vs_N/DAG/IL-1b_vs_N.DEG_down_cc_DAG.pdf]

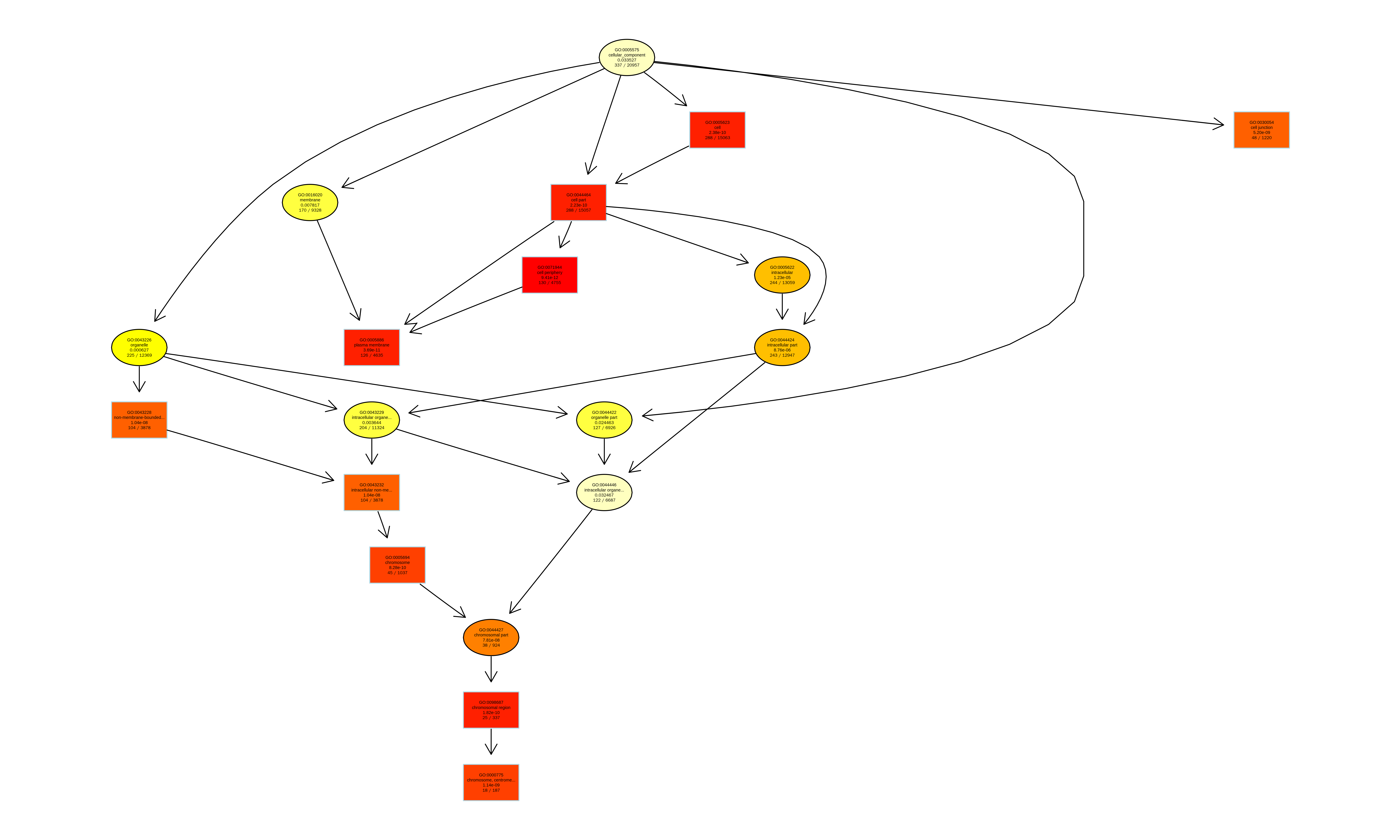

Supplement: Supplementary file 1 [file molecules-28-01606-s001.zip › raw data/GO/IL-1b_vs_N/DAG/IL-1b_vs_N.DEG_down_cc_DAG.png]

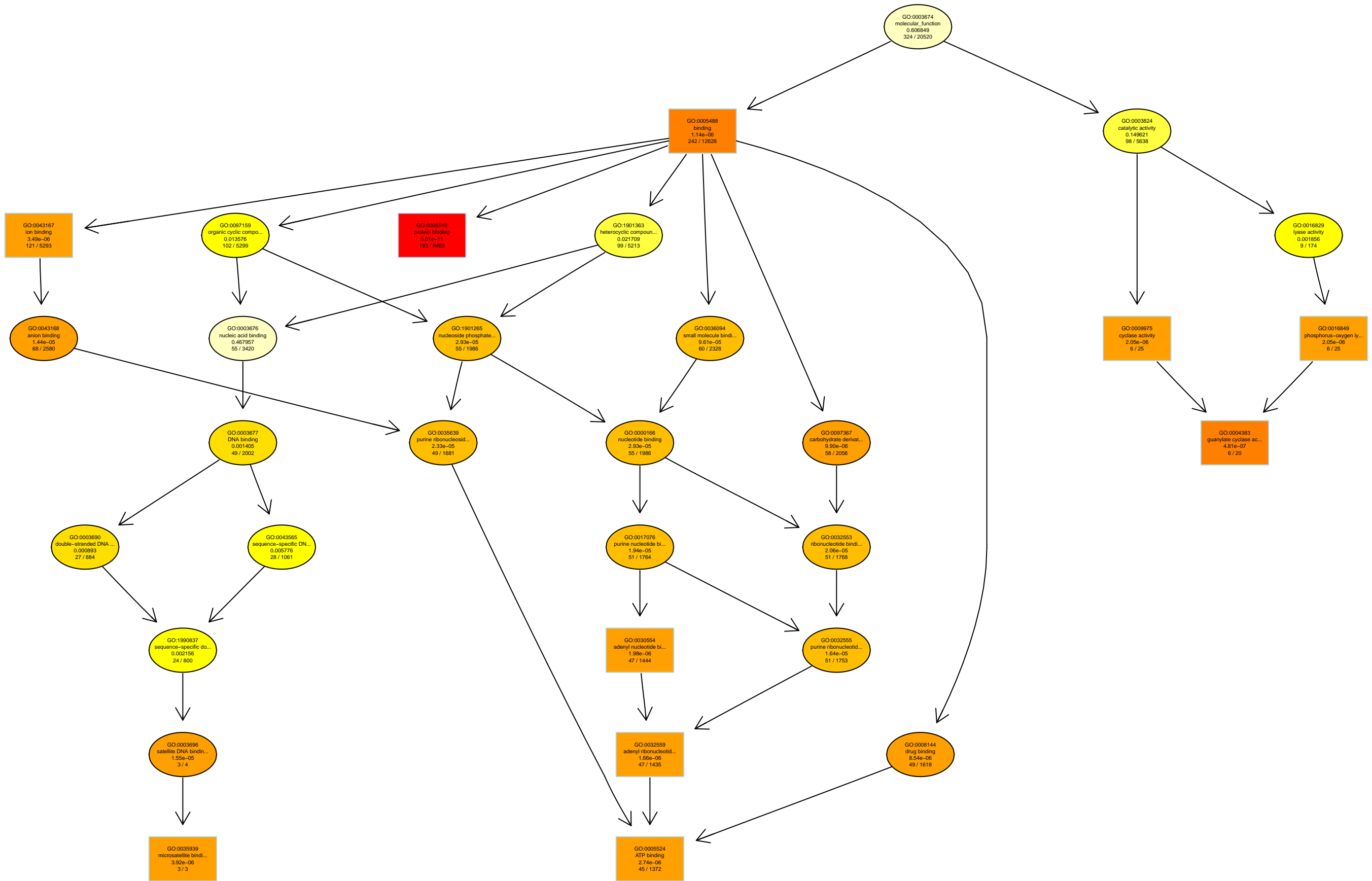

Supplement: Supplementary file 1 [file molecules-28-01606-s001.zip › raw data/GO/IL-1b_vs_N/DAG/IL-1b_vs_N.DEG_down_mf_DAG.pdf]

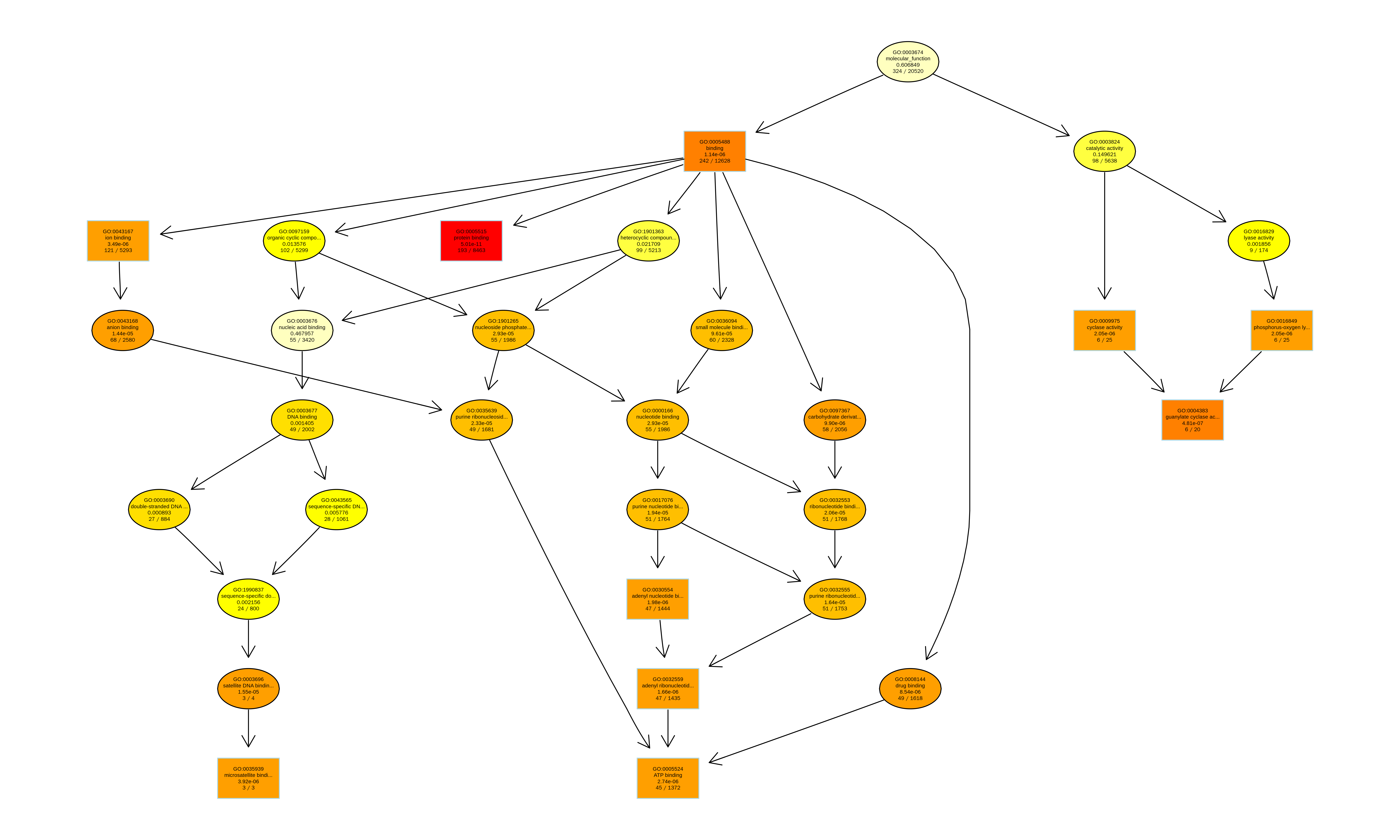

Supplement: Supplementary file 1 [file molecules-28-01606-s001.zip › raw data/GO/IL-1b_vs_N/DAG/IL-1b_vs_N.DEG_down_mf_DAG.png]

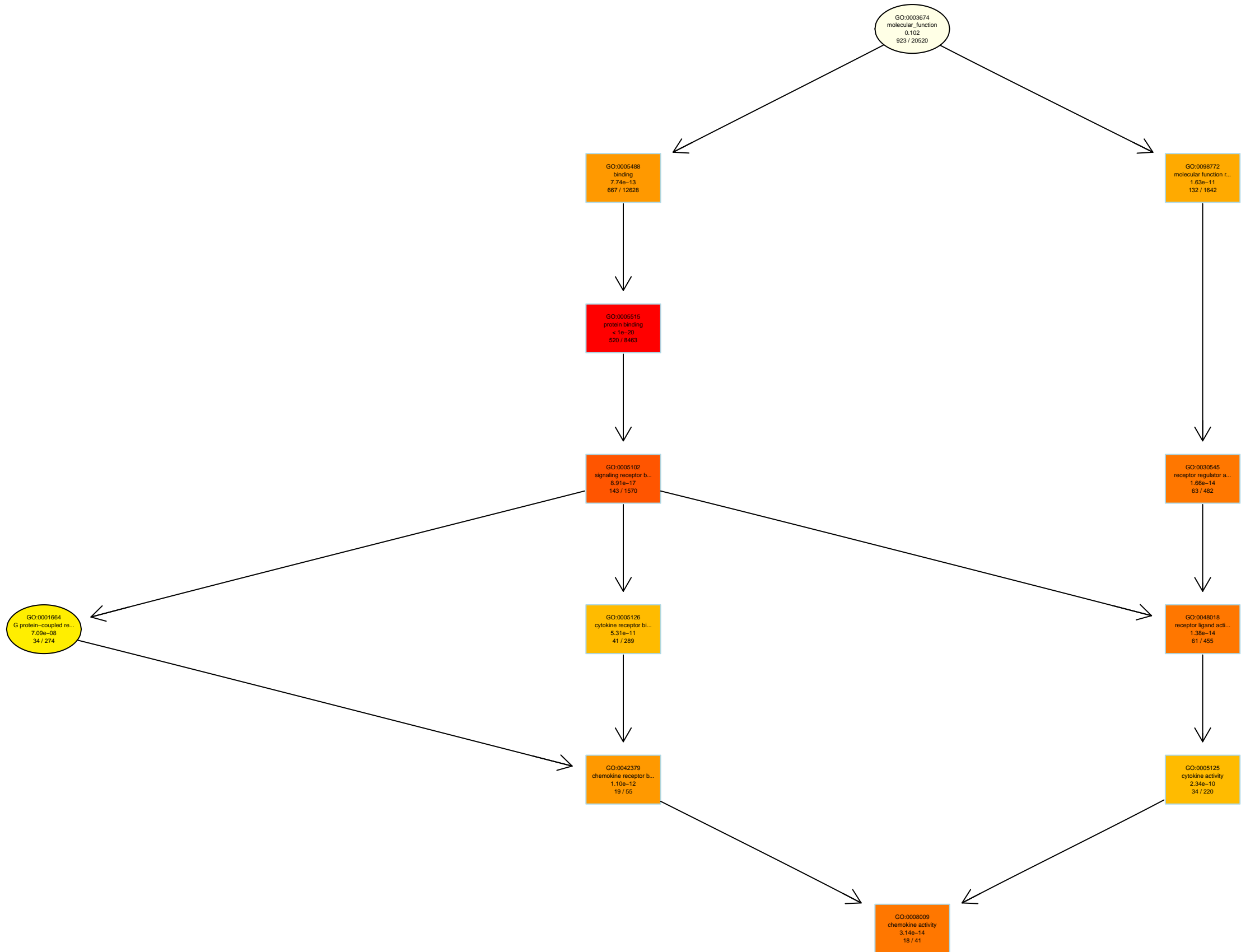

Supplement: Supplementary file 1 [file molecules-28-01606-s001.zip › raw data/GO/IL-1b_vs_N/DAG/IL-1b_vs_N.DEG_mf_DAG.pdf]

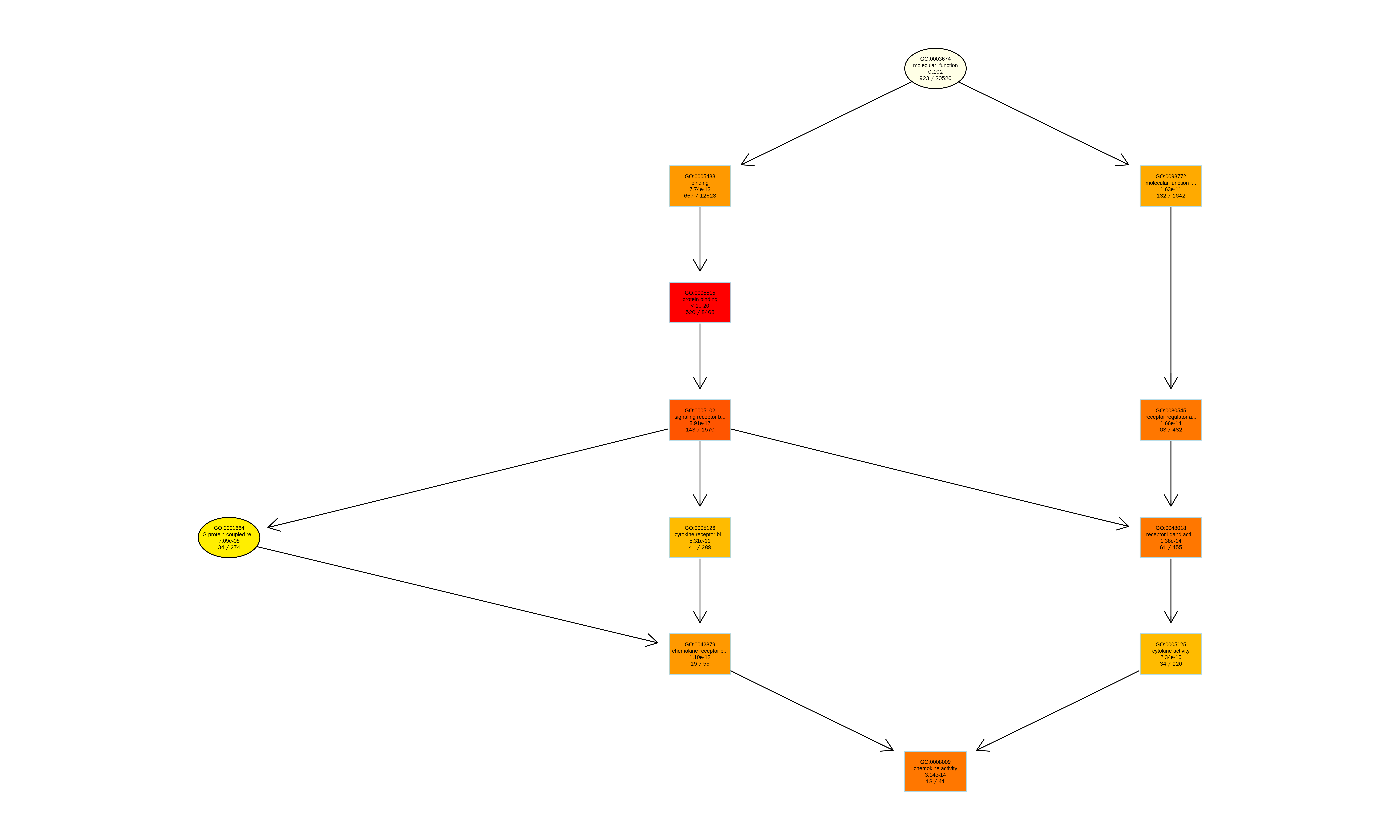

Supplement: Supplementary file 1 [file molecules-28-01606-s001.zip › raw data/GO/IL-1b_vs_N/DAG/IL-1b_vs_N.DEG_mf_DAG.png]

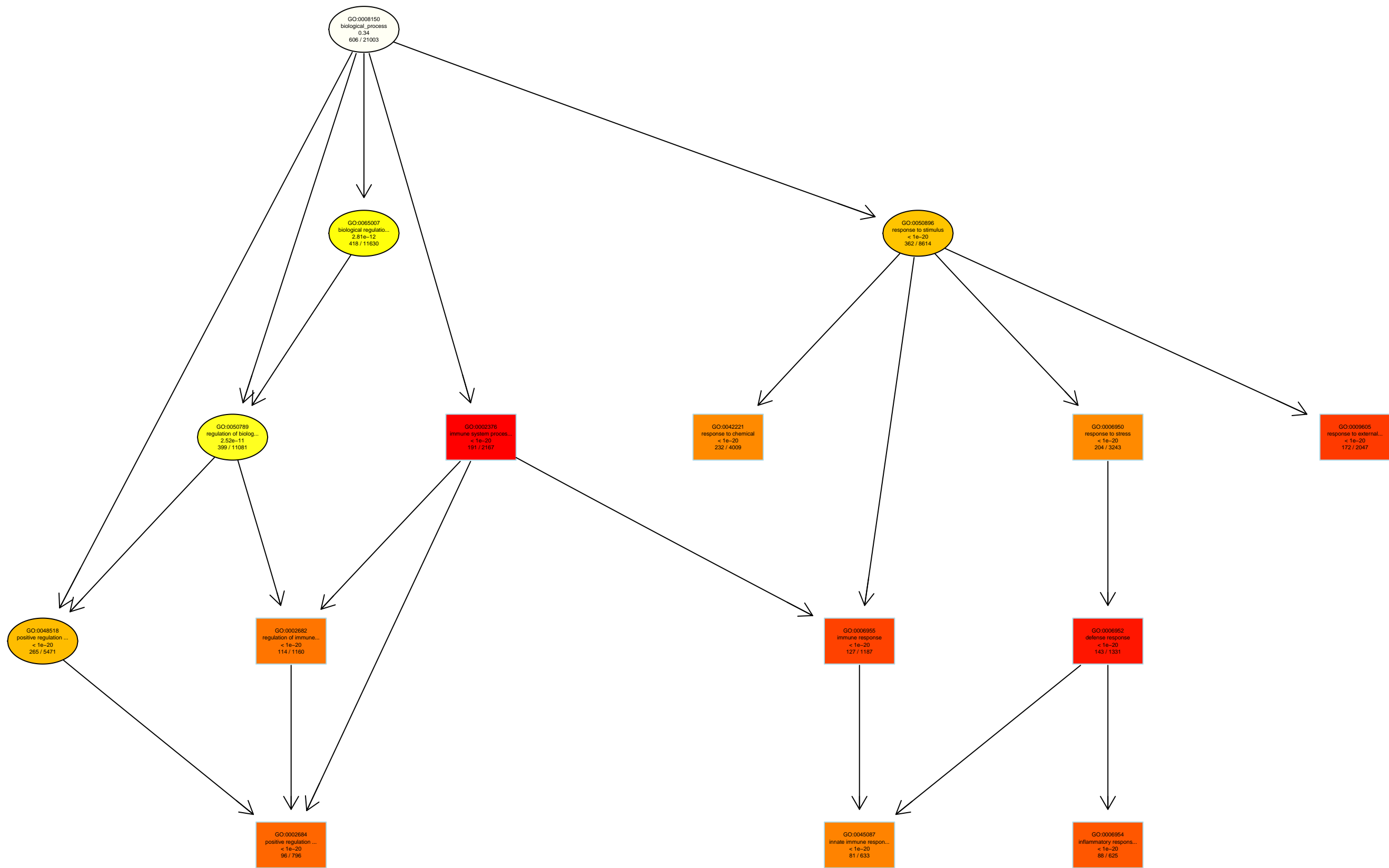

Supplement: Supplementary file 1 [file molecules-28-01606-s001.zip › raw data/GO/IL-1b_vs_N/DAG/IL-1b_vs_N.DEG_up_bp_DAG.pdf]

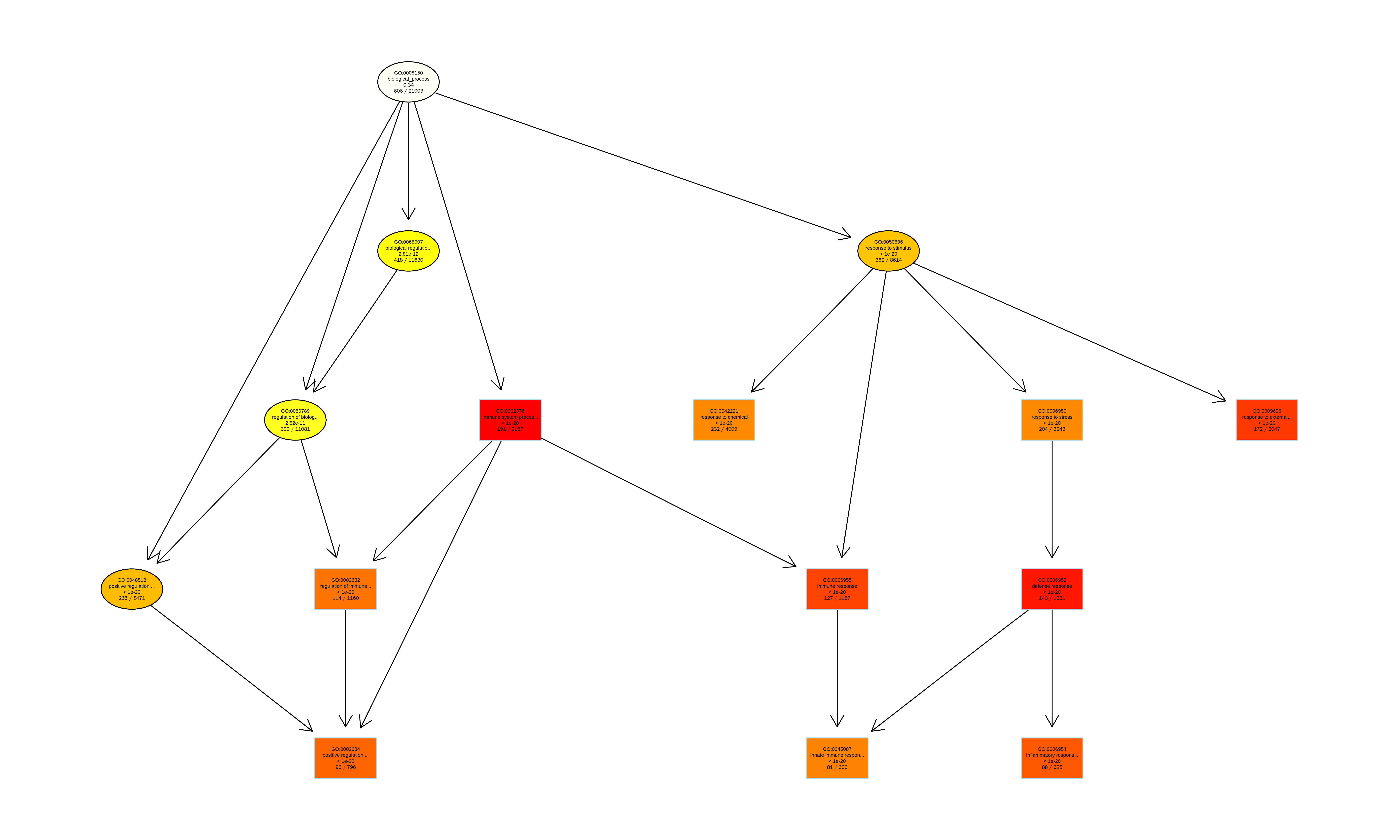

Supplement: Supplementary file 1 [file molecules-28-01606-s001.zip › raw data/GO/IL-1b_vs_N/DAG/IL-1b_vs_N.DEG_up_bp_DAG.png]

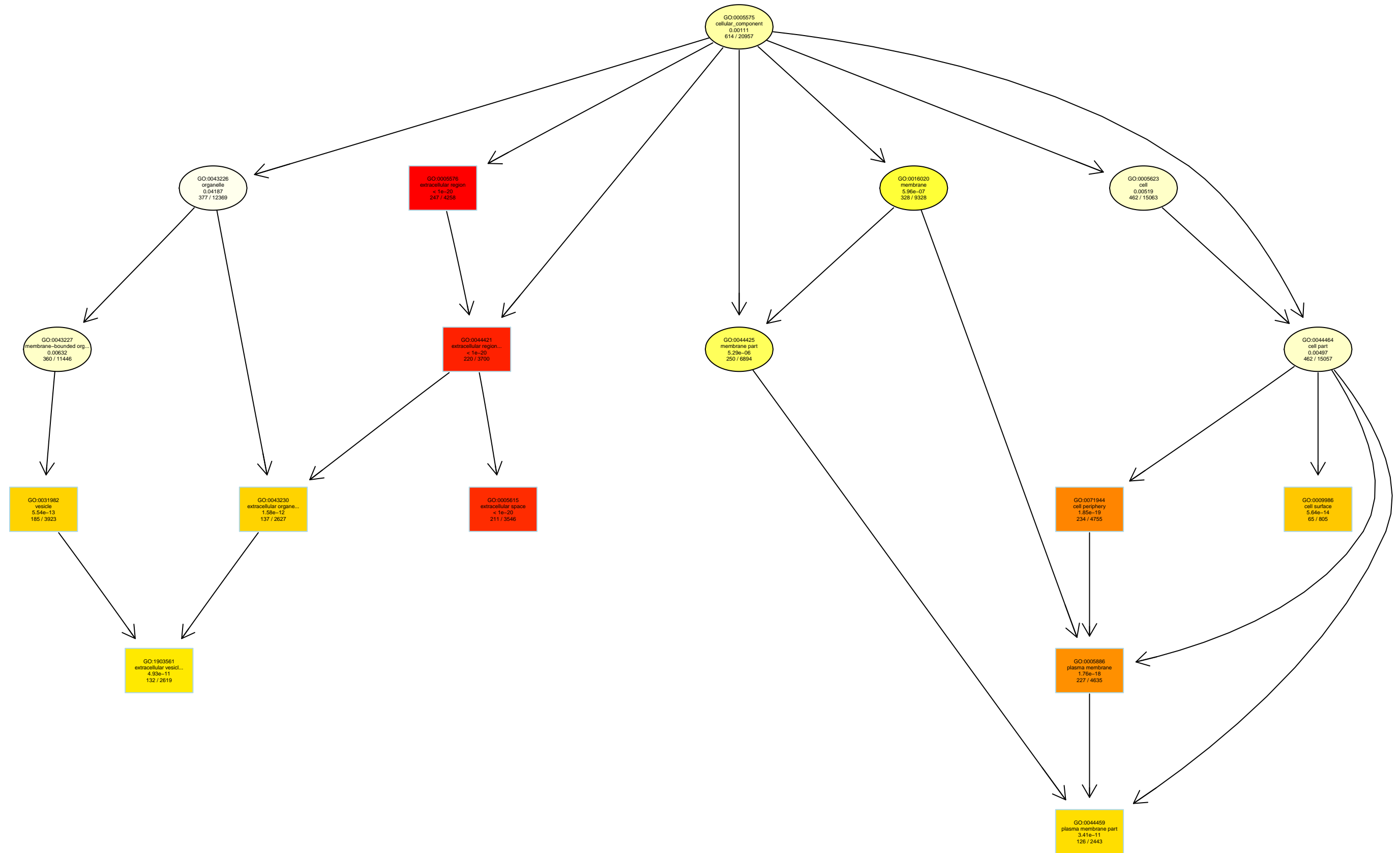

Supplement: Supplementary file 1 [file molecules-28-01606-s001.zip › raw data/GO/IL-1b_vs_N/DAG/IL-1b_vs_N.DEG_up_cc_DAG.pdf]

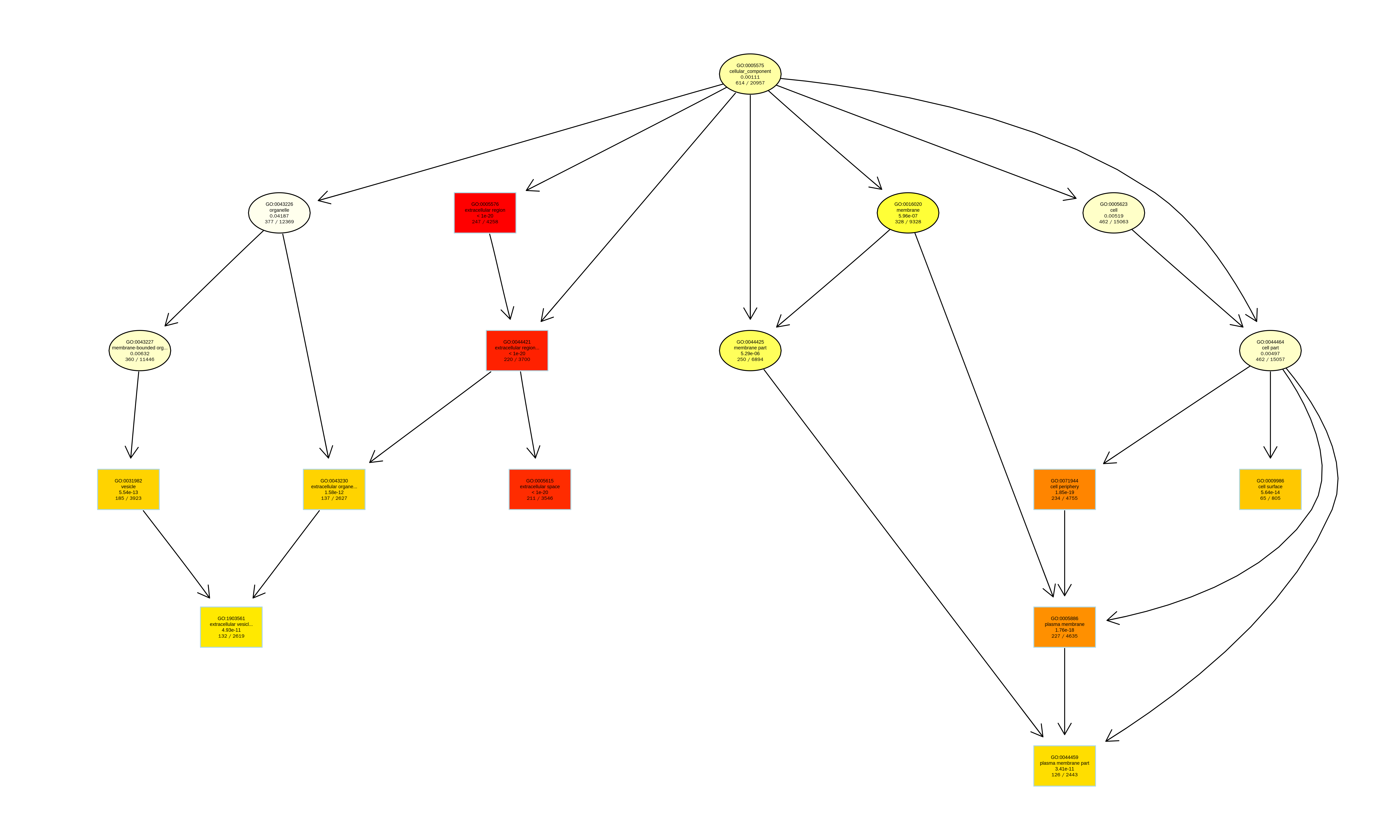

Supplement: Supplementary file 1 [file molecules-28-01606-s001.zip › raw data/GO/IL-1b_vs_N/DAG/IL-1b_vs_N.DEG_up_cc_DAG.png]

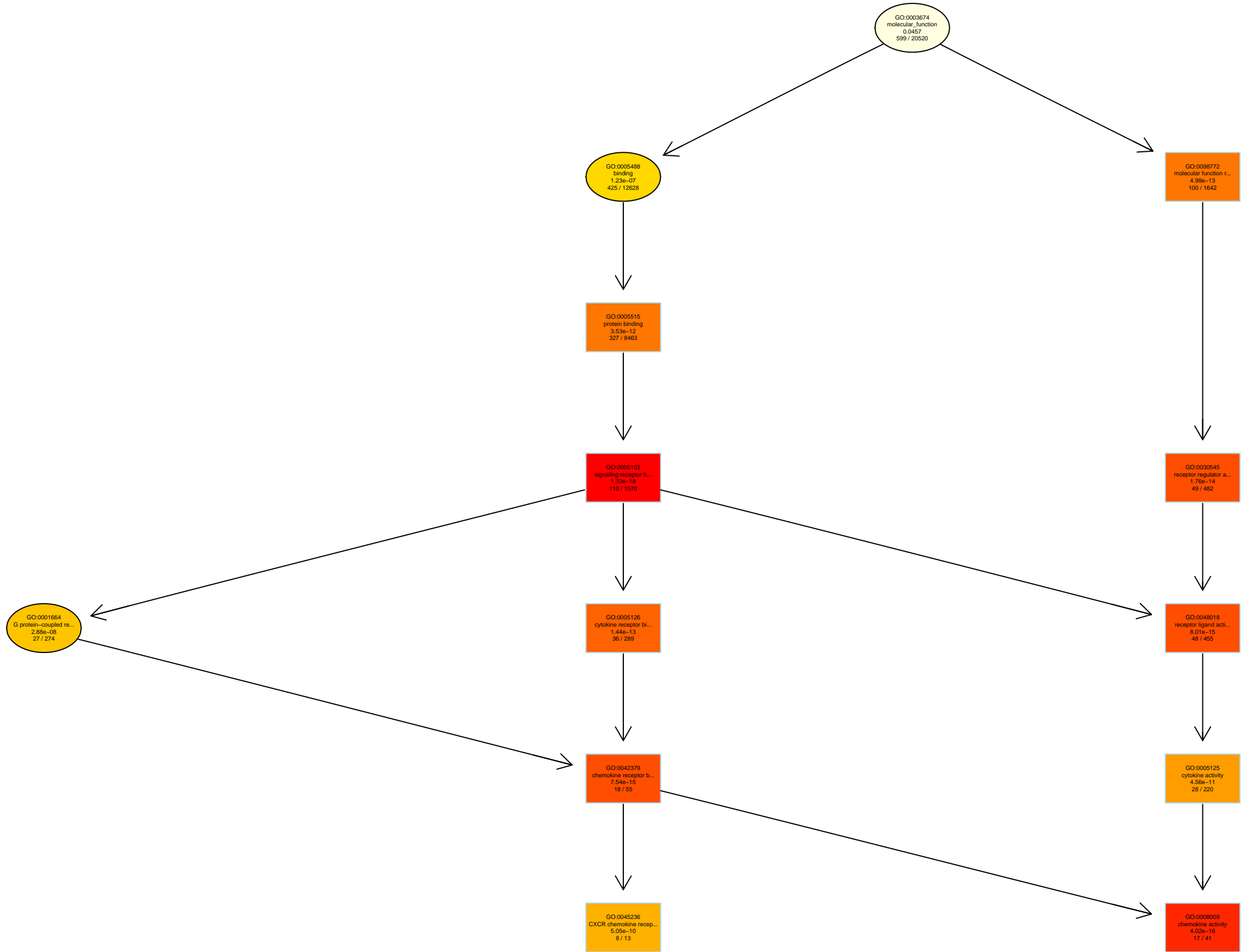

Supplement: Supplementary file 1 [file molecules-28-01606-s001.zip › raw data/GO/IL-1b_vs_N/DAG/IL-1b_vs_N.DEG_up_mf_DAG.pdf]

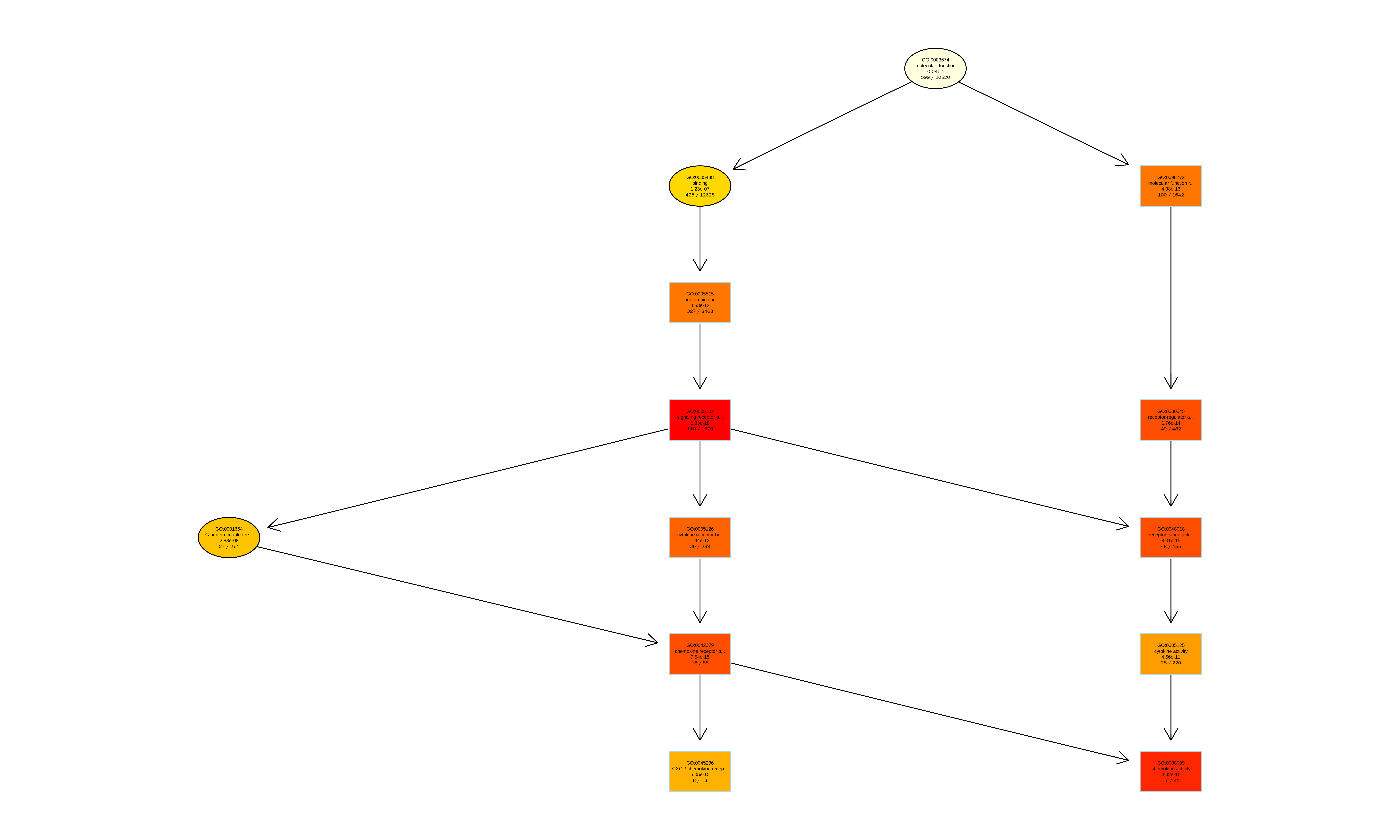

Supplement: Supplementary file 1 [file molecules-28-01606-s001.zip › raw data/GO/IL-1b_vs_N/DAG/IL-1b_vs_N.DEG_up_mf_DAG.png]

# Enriched GO Terms (Top 30)

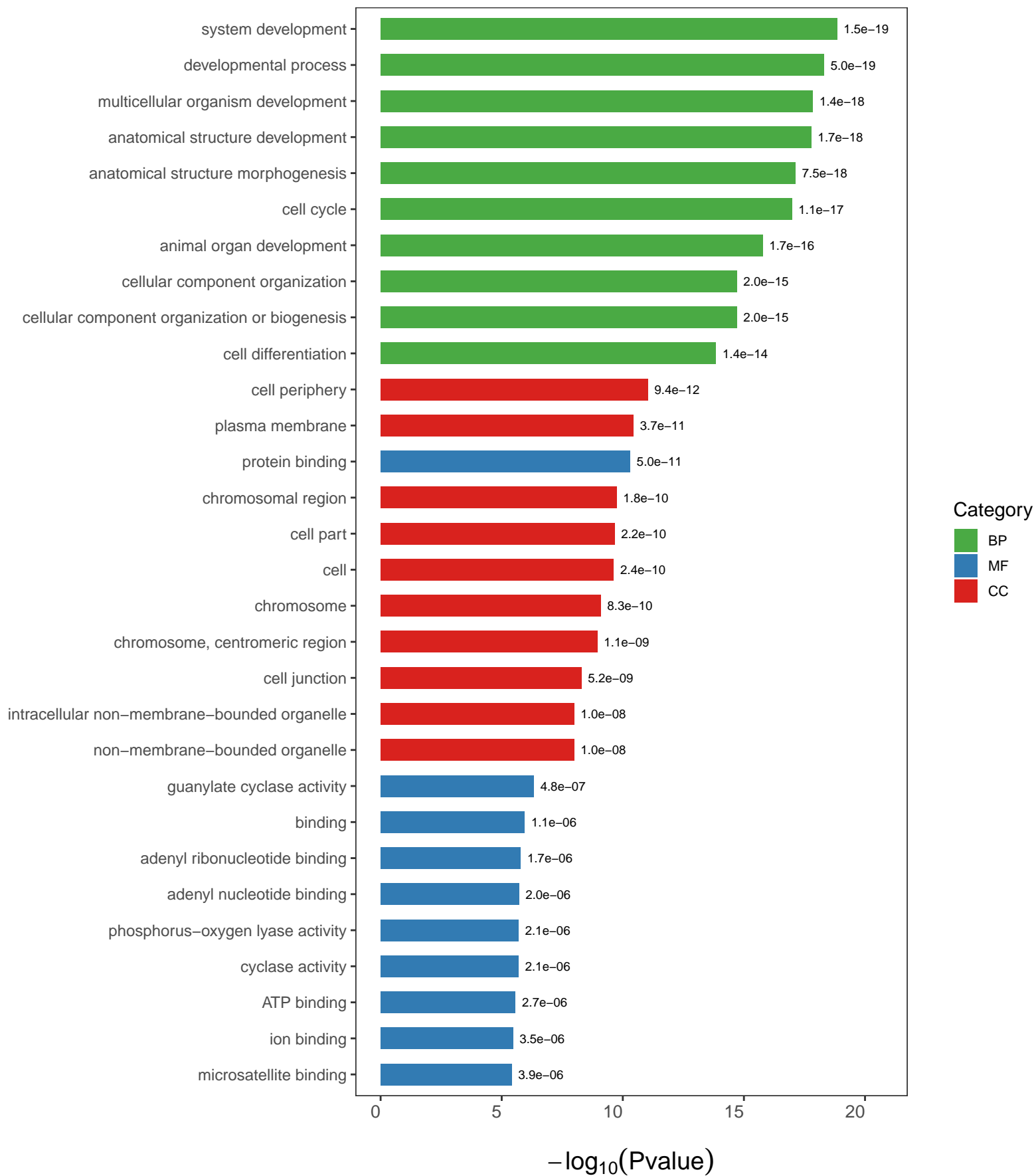

Supplement: Supplementary file 1 [file molecules-28-01606-s001.zip › raw data/GO/IL-1b_vs_N/Enrich_bar/IL-1b_vs_N.DEG_down_GO_Enriched_bar.pdf]

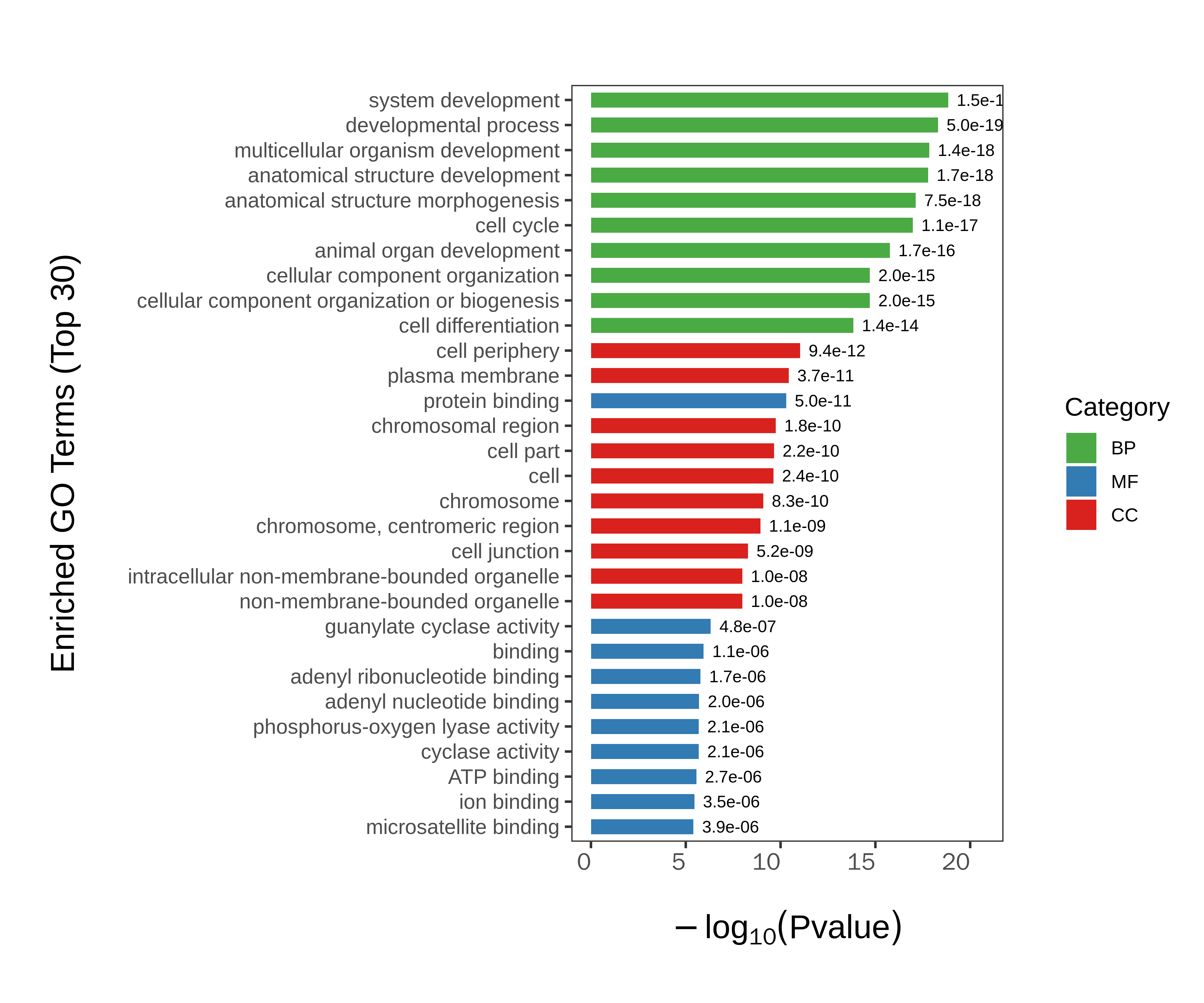

Supplement: Supplementary file 1 [file molecules-28-01606-s001.zip › raw data/GO/IL-1b_vs_N/Enrich_bar/IL-1b_vs_N.DEG_down_GO_Enriched_bar.png]

Enriched GO Terms (Top 30)

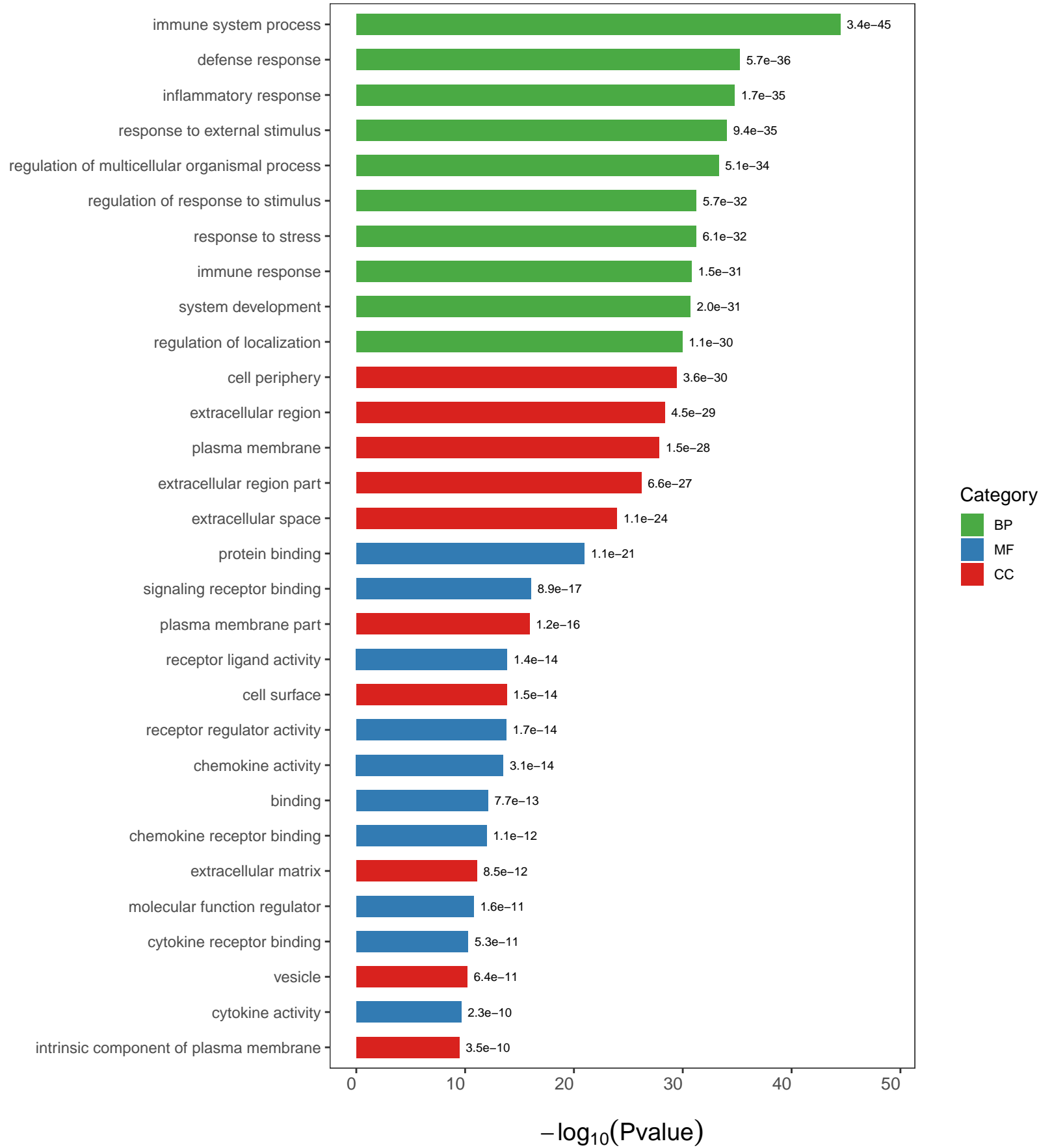

Supplement: Supplementary file 1 [file molecules-28-01606-s001.zip › raw data/GO/IL-1b_vs_N/Enrich_bar/IL-1b_vs_N.DEG_GO_Enriched_bar.pdf]

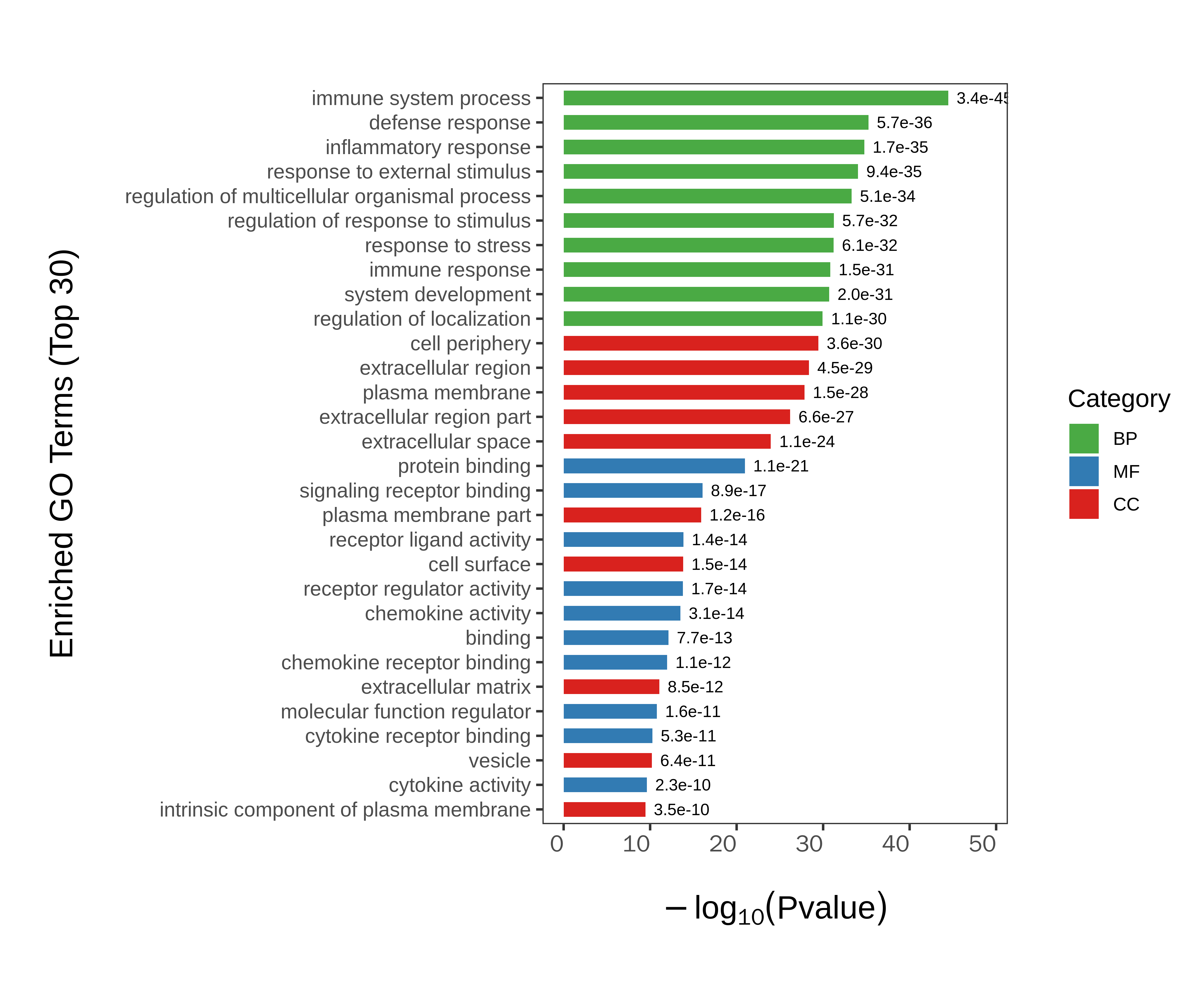

Supplement: Supplementary file 1 [file molecules-28-01606-s001.zip › raw data/GO/IL-1b_vs_N/Enrich_bar/IL-1b_vs_N.DEG_GO_Enriched_bar.png]

# Enriched GO Terms (Top 30)

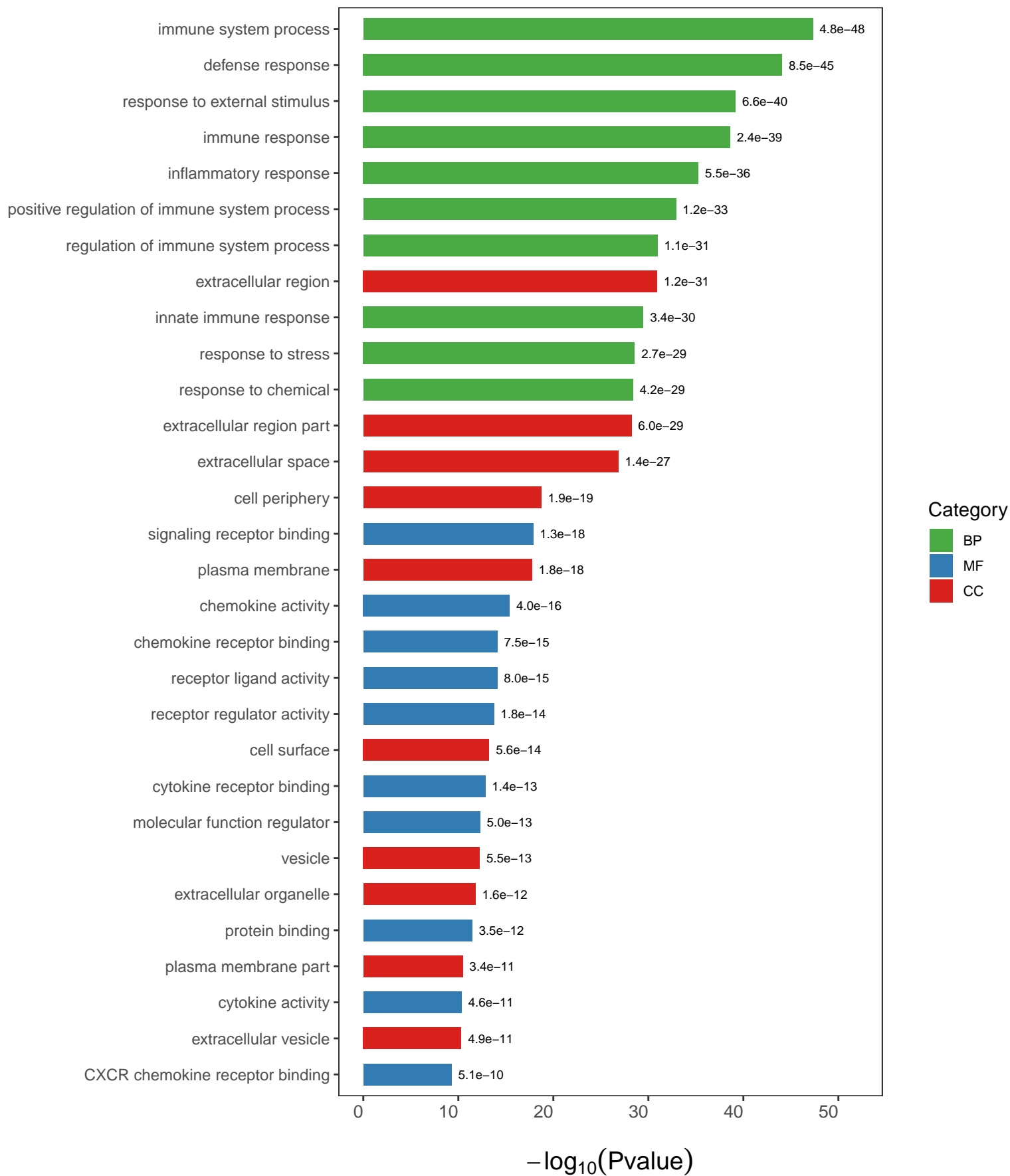

Supplement: Supplementary file 1 [file molecules-28-01606-s001.zip › raw data/GO/IL-1b_vs_N/Enrich_bar/IL-1b_vs_N.DEG_up_GO_Enriched_bar.pdf]

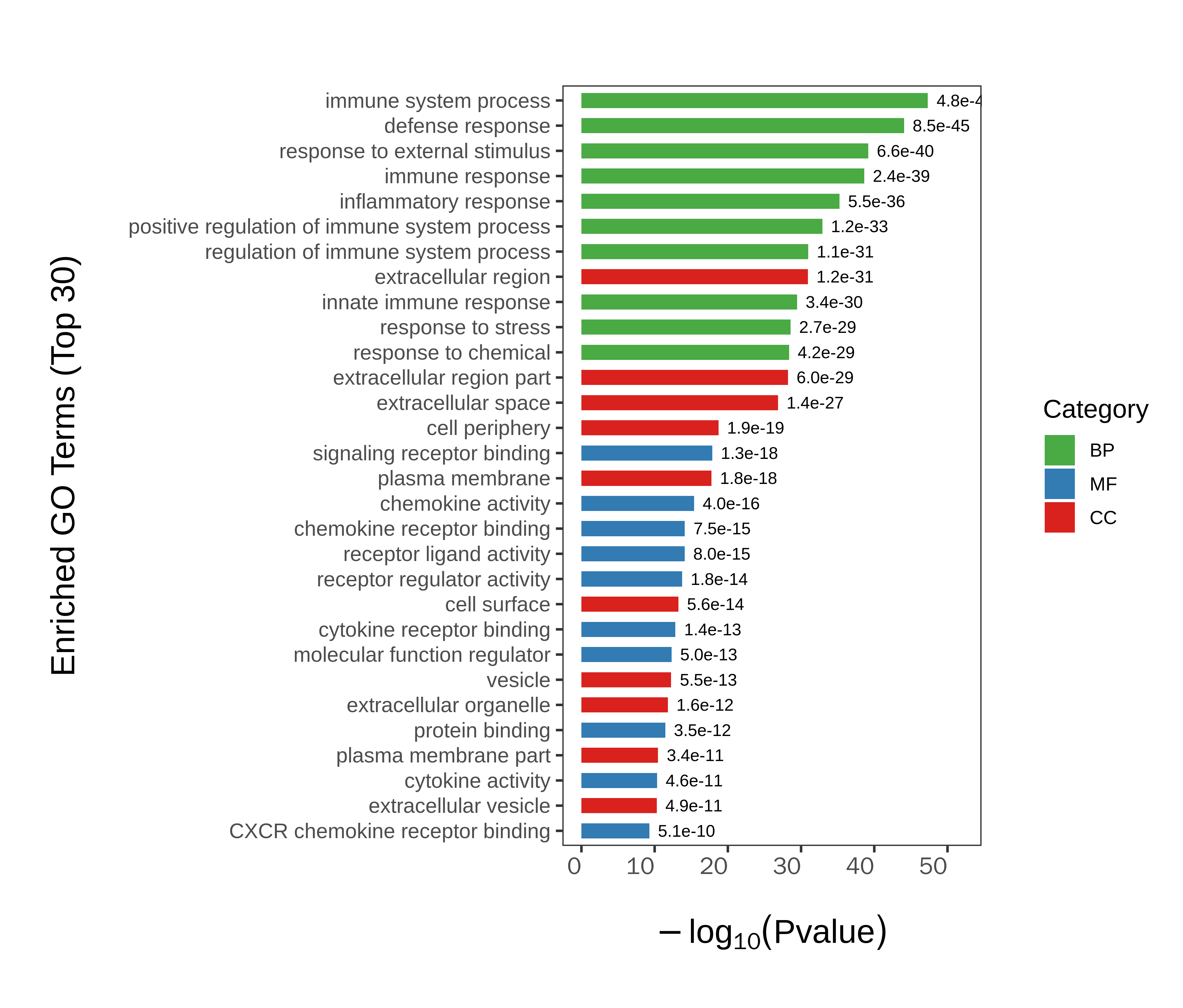

Supplement: Supplementary file 1 [file molecules-28-01606-s001.zip › raw data/GO/IL-1b_vs_N/Enrich_bar/IL-1b_vs_N.DEG_up_GO_Enriched_bar.png]

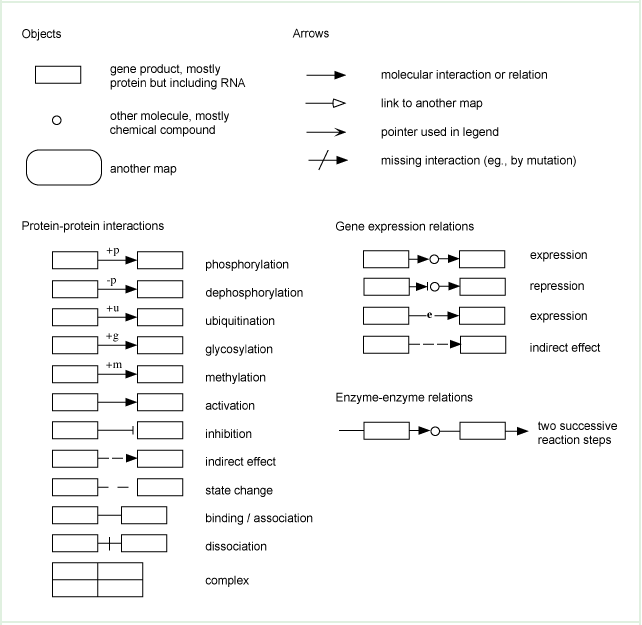

Supplement: Supplementary file 1 [file molecules-28-01606-s001.zip › raw data/KEGG/IL-1b_vs_N/DEG_pathway/legend.png]

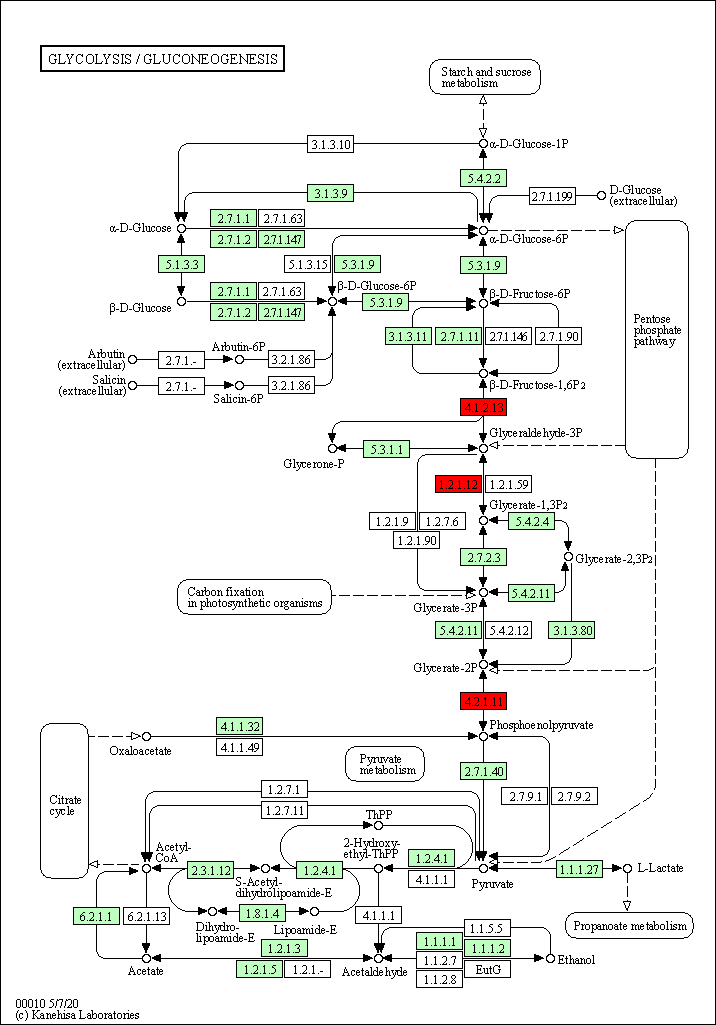

Supplement: Supplementary file 1 [file molecules-28-01606-s001.zip › raw data/KEGG/IL-1b_vs_N/DEG_pathway/mmu00010.png]

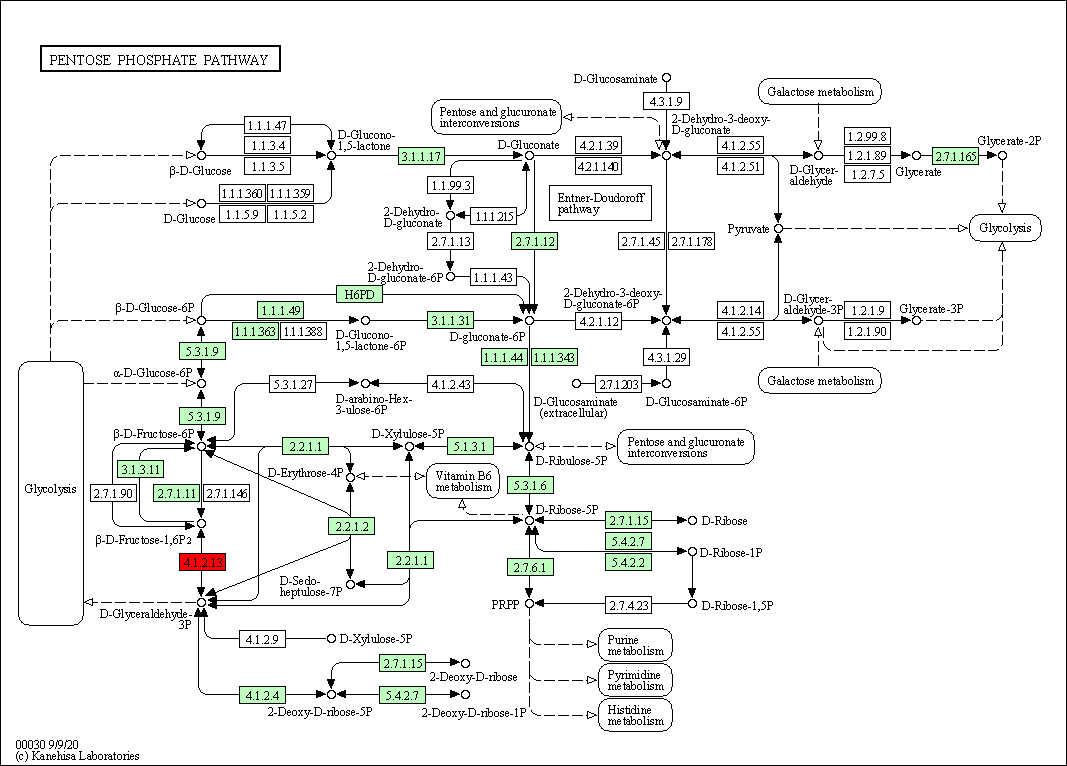

Supplement: Supplementary file 1 [file molecules-28-01606-s001.zip › raw data/KEGG/IL-1b_vs_N/DEG_pathway/mmu00030.png]

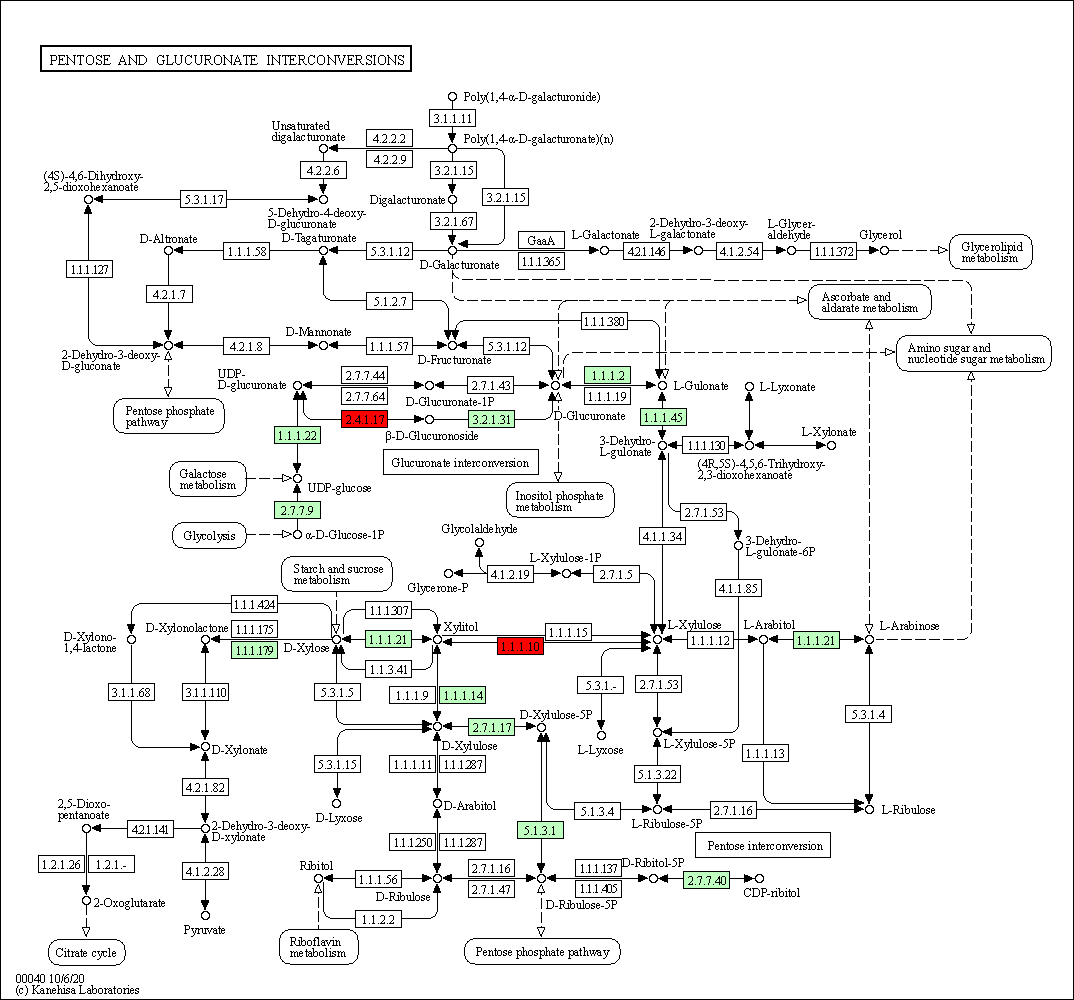

Supplement: Supplementary file 1 [file molecules-28-01606-s001.zip › raw data/KEGG/IL-1b_vs_N/DEG_pathway/mmu00040.png]

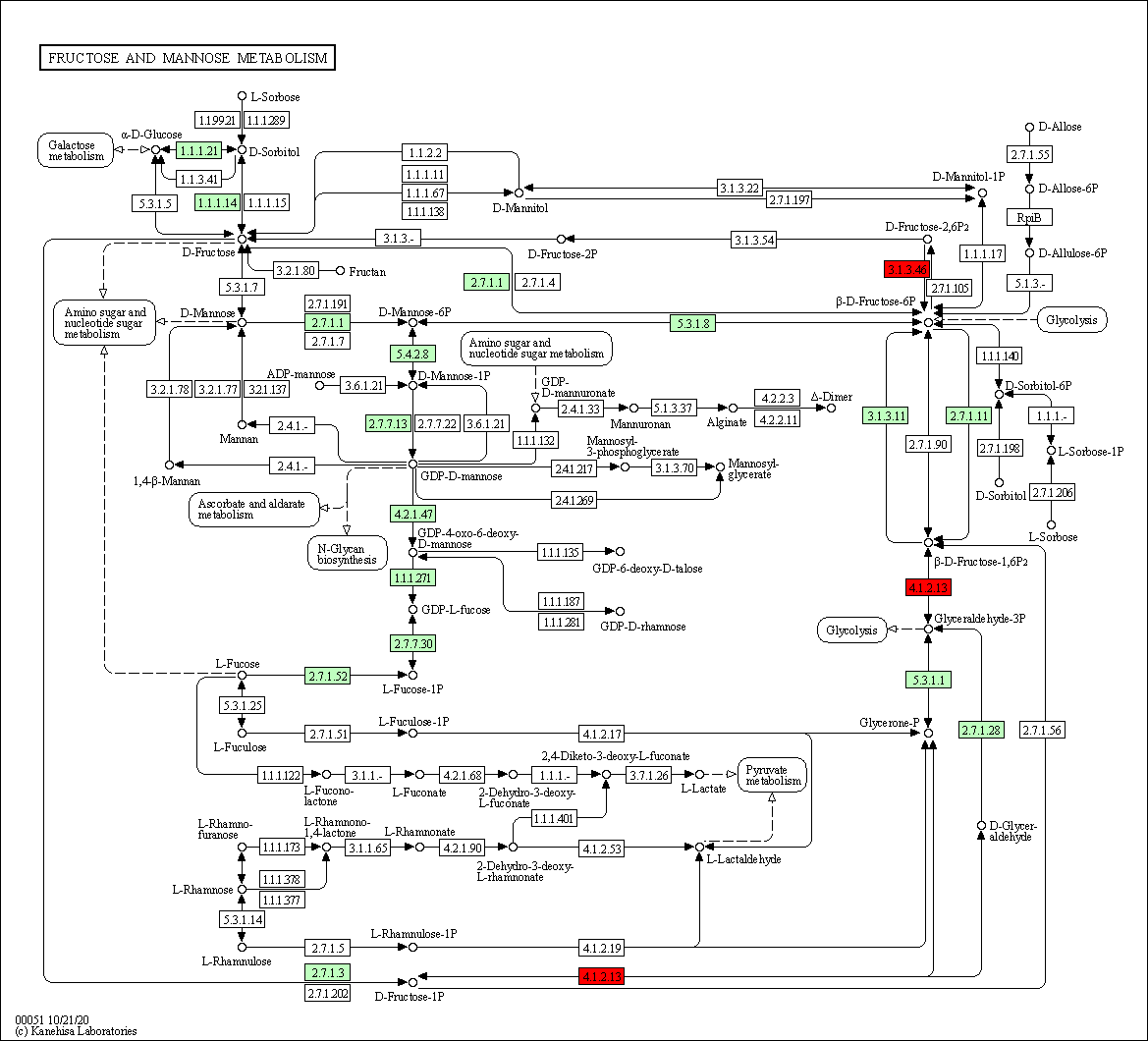

Supplement: Supplementary file 1 [file molecules-28-01606-s001.zip › raw data/KEGG/IL-1b_vs_N/DEG_pathway/mmu00051.png]

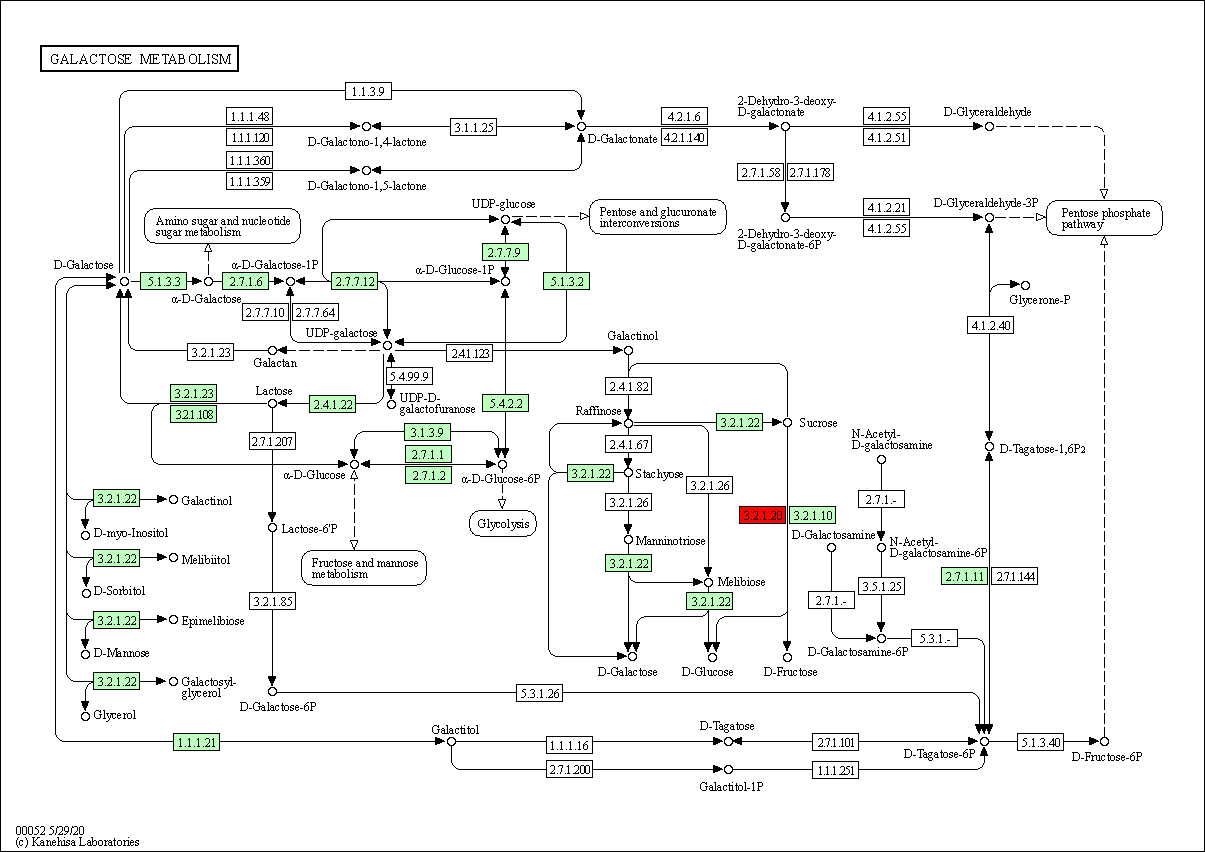

Supplement: Supplementary file 1 [file molecules-28-01606-s001.zip › raw data/KEGG/IL-1b_vs_N/DEG_pathway/mmu00052.png]

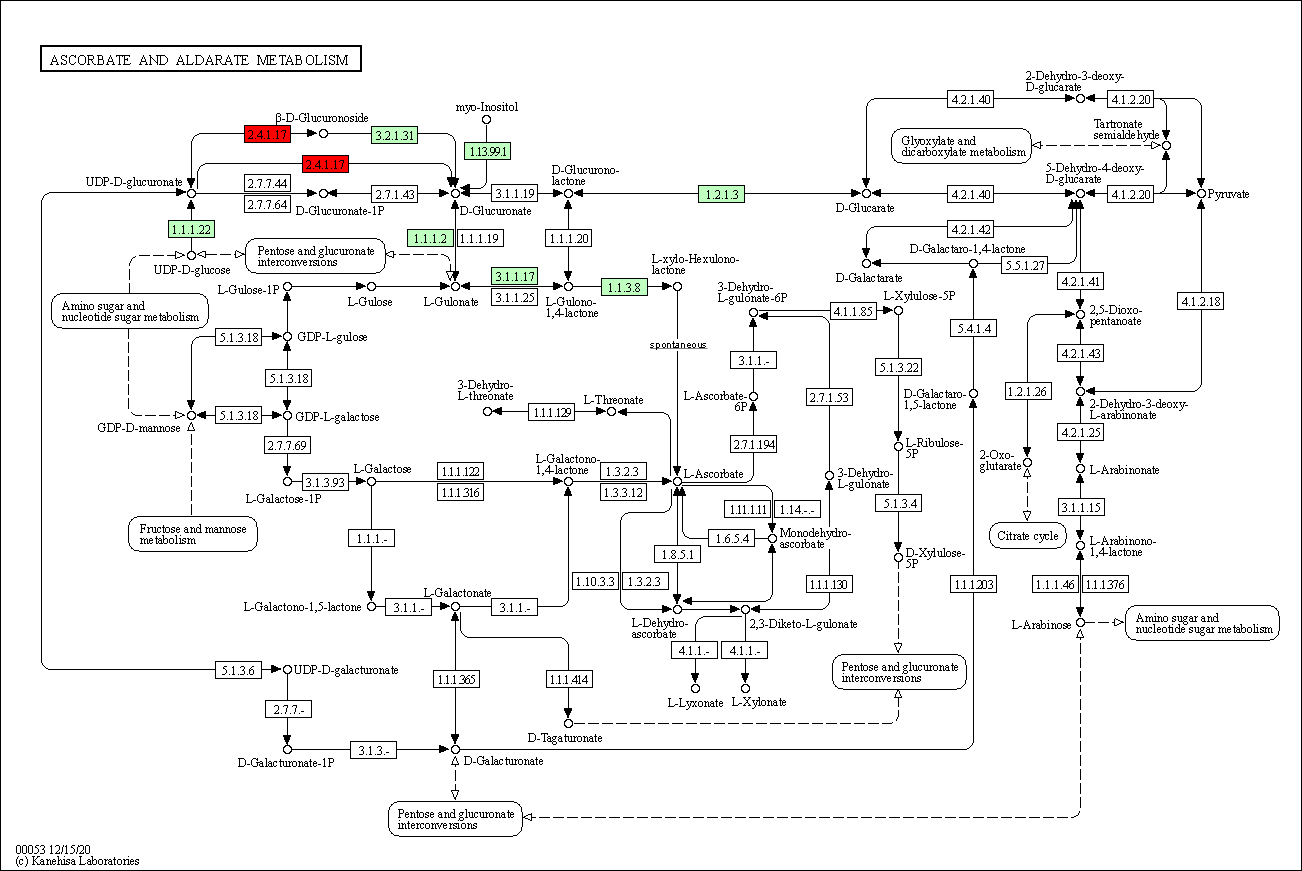

Supplement: Supplementary file 1 [file molecules-28-01606-s001.zip › raw data/KEGG/IL-1b_vs_N/DEG_pathway/mmu00053.png]

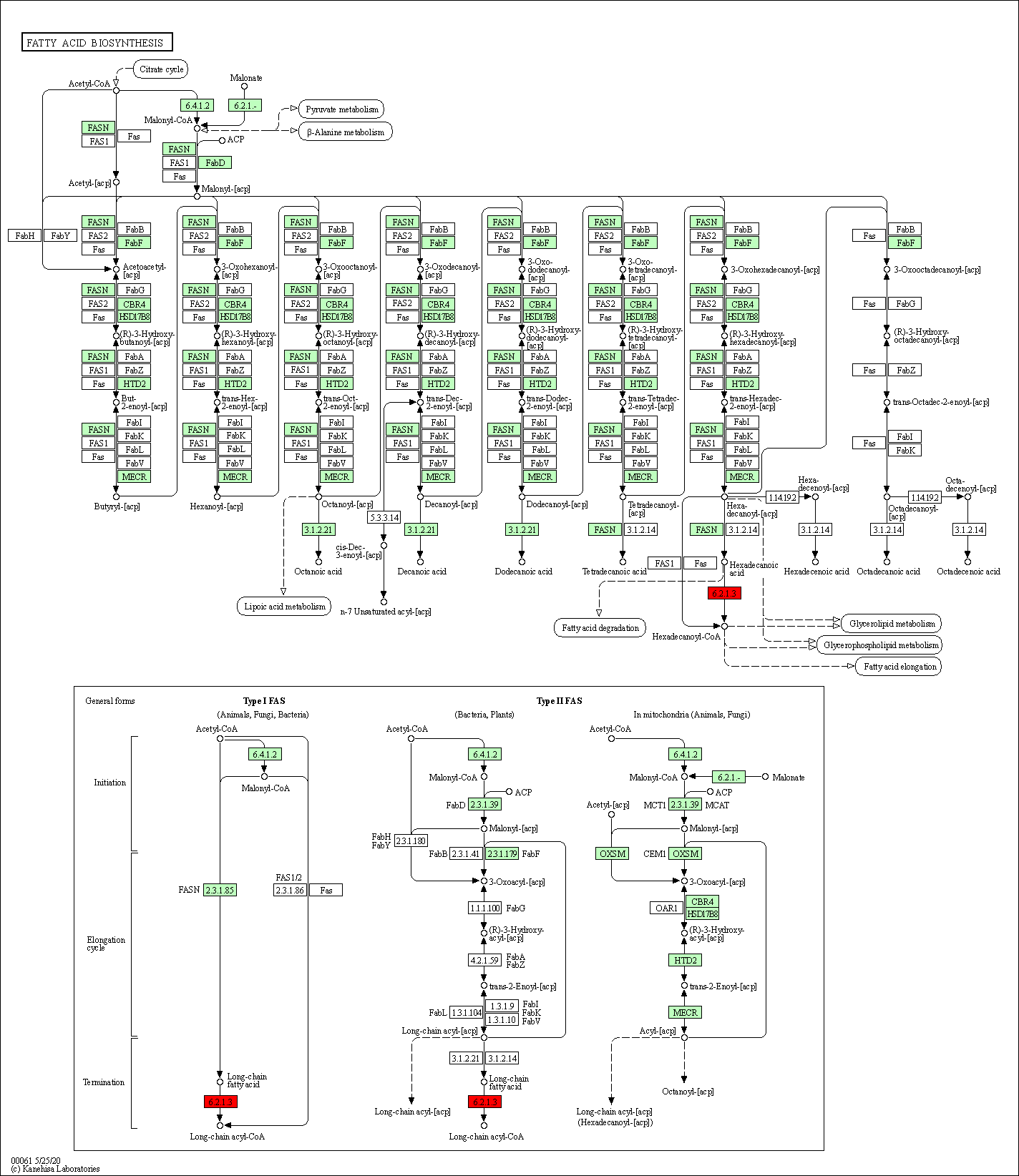

Supplement: Supplementary file 1 [file molecules-28-01606-s001.zip › raw data/KEGG/IL-1b_vs_N/DEG_pathway/mmu00061.png]

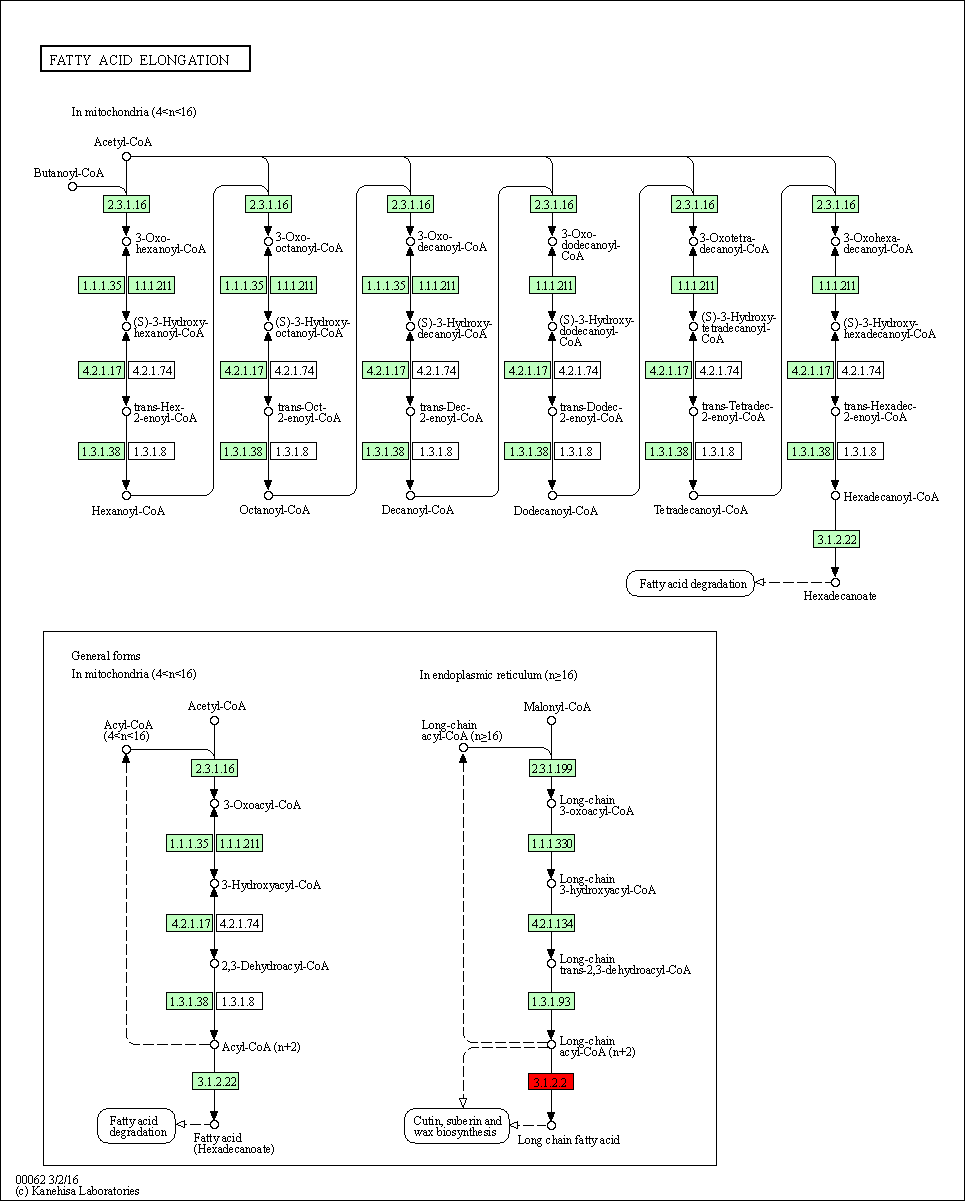

Supplement: Supplementary file 1 [file molecules-28-01606-s001.zip › raw data/KEGG/IL-1b_vs_N/DEG_pathway/mmu00062.png]

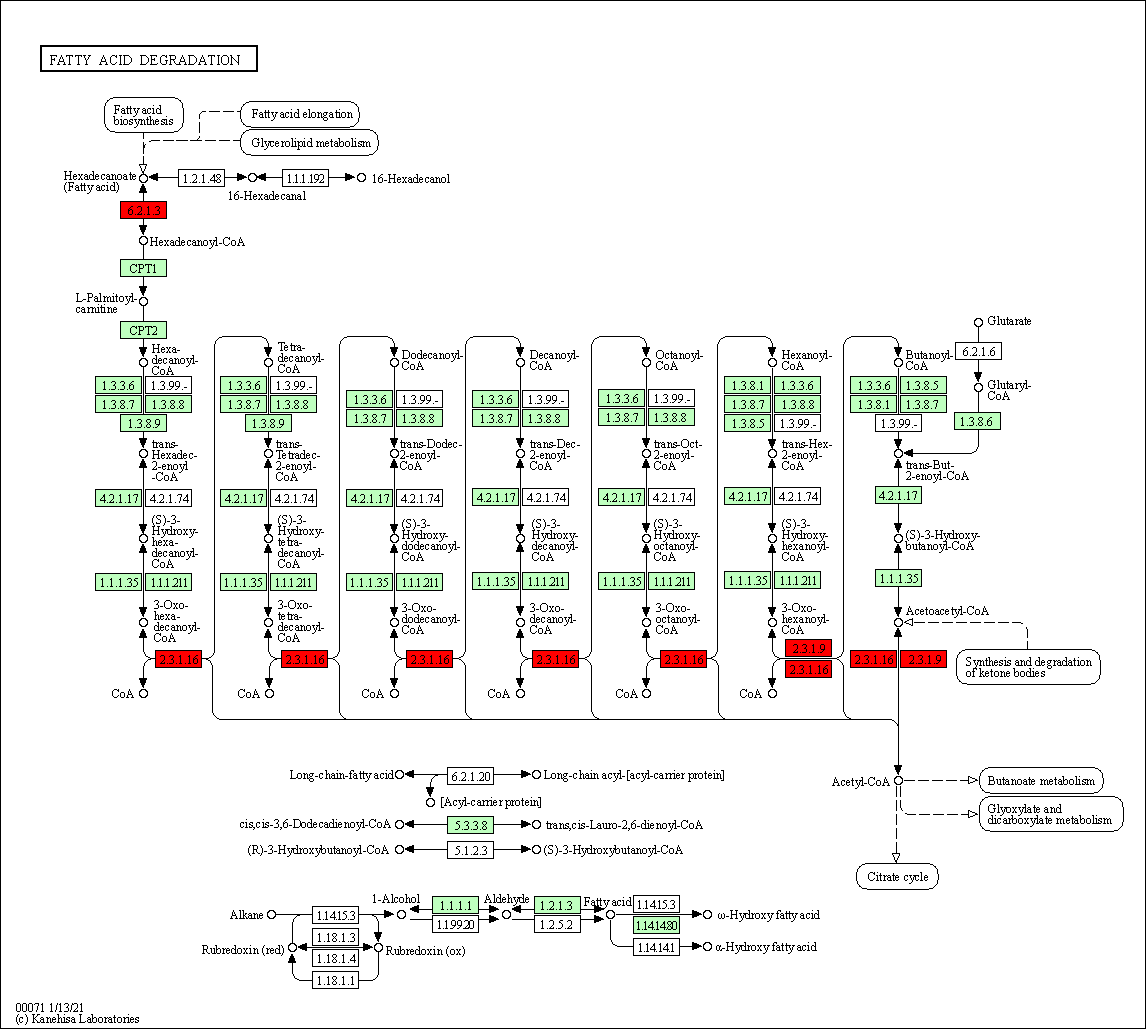

Supplement: Supplementary file 1 [file molecules-28-01606-s001.zip › raw data/KEGG/IL-1b_vs_N/DEG_pathway/mmu00071.png]

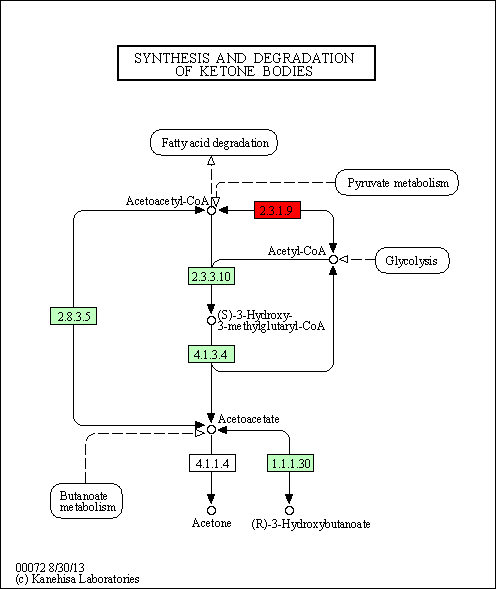

Supplement: Supplementary file 1 [file molecules-28-01606-s001.zip › raw data/KEGG/IL-1b_vs_N/DEG_pathway/mmu00072.png]

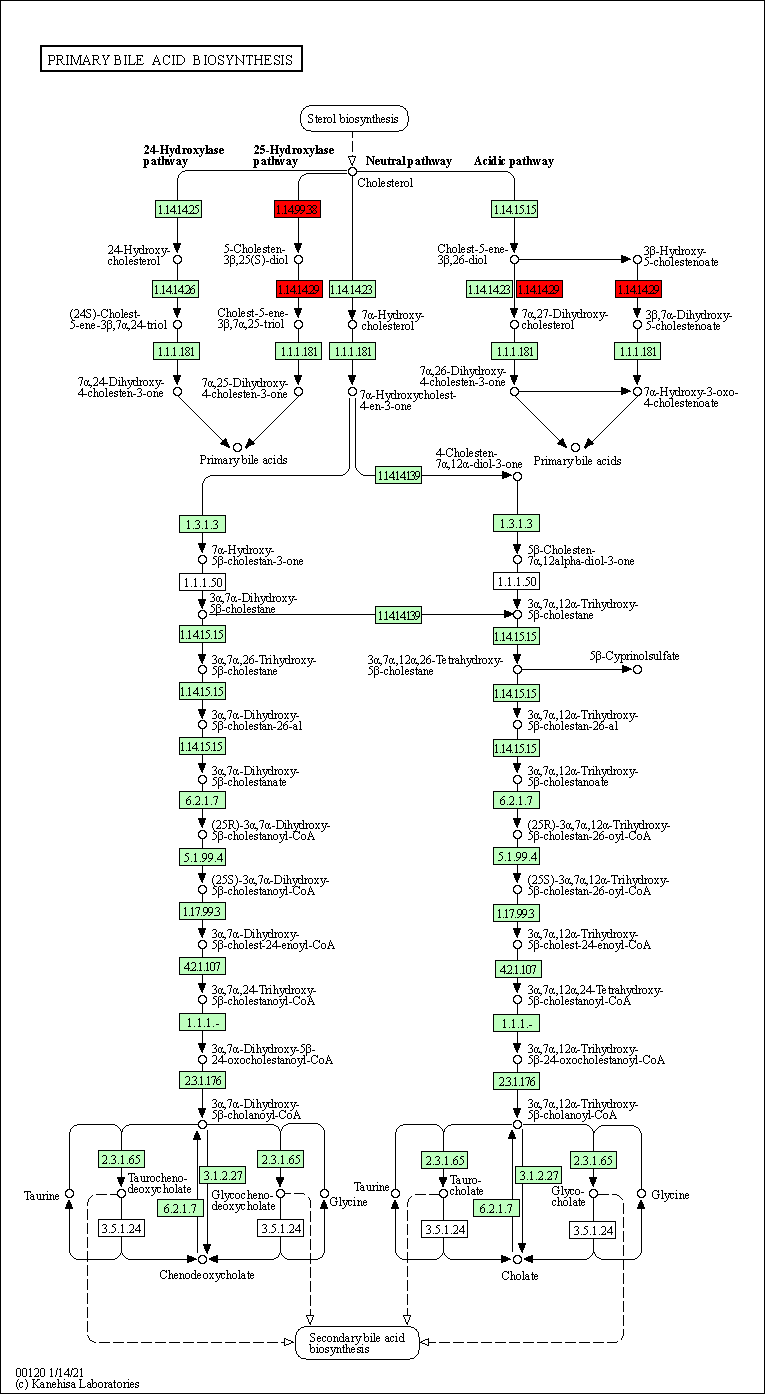

Supplement: Supplementary file 1 [file molecules-28-01606-s001.zip › raw data/KEGG/IL-1b_vs_N/DEG_pathway/mmu00120.png]

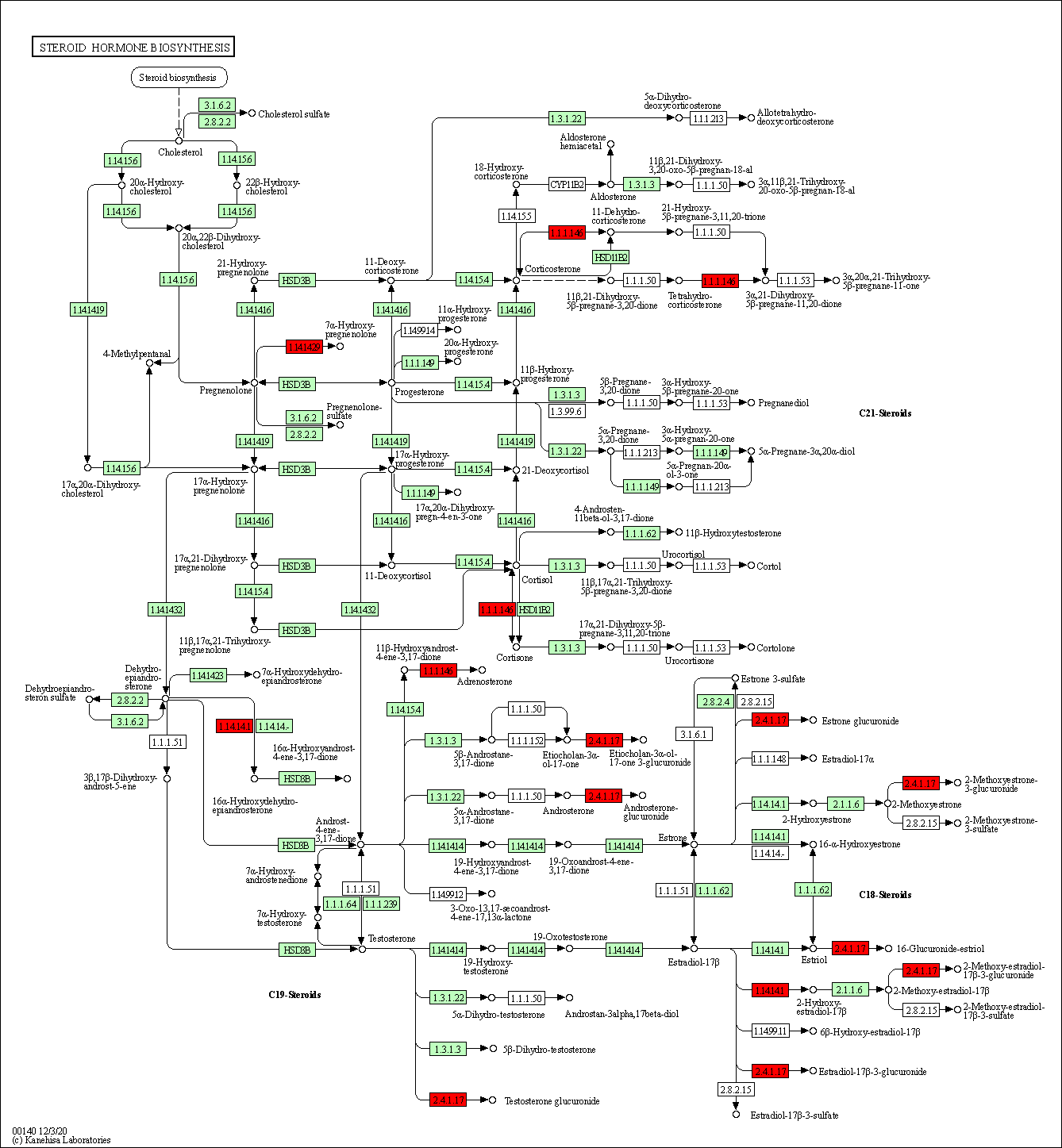

Supplement: Supplementary file 1 [file molecules-28-01606-s001.zip › raw data/KEGG/IL-1b_vs_N/DEG_pathway/mmu00140.png]

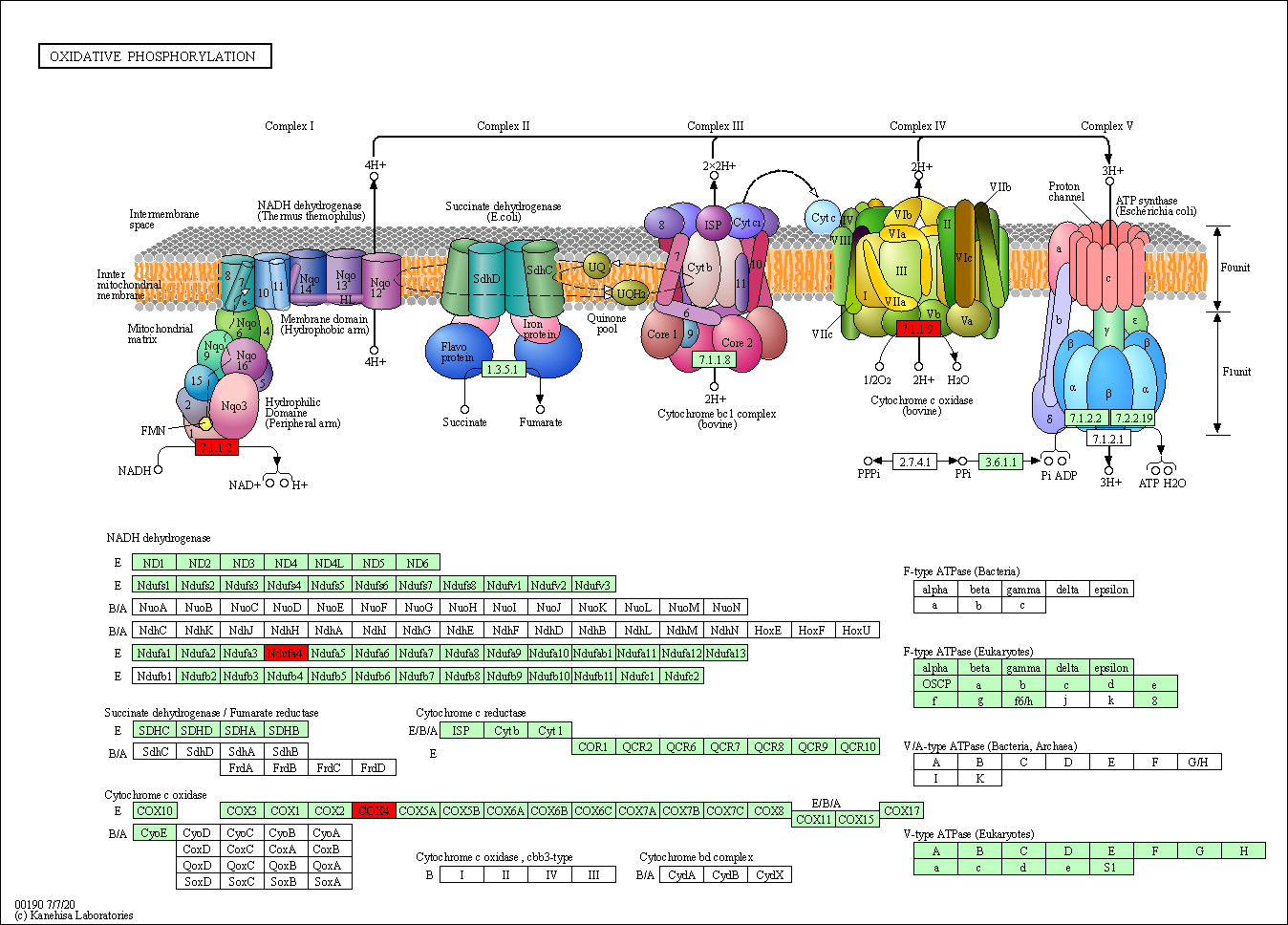

Supplement: Supplementary file 1 [file molecules-28-01606-s001.zip › raw data/KEGG/IL-1b_vs_N/DEG_pathway/mmu00190.png]

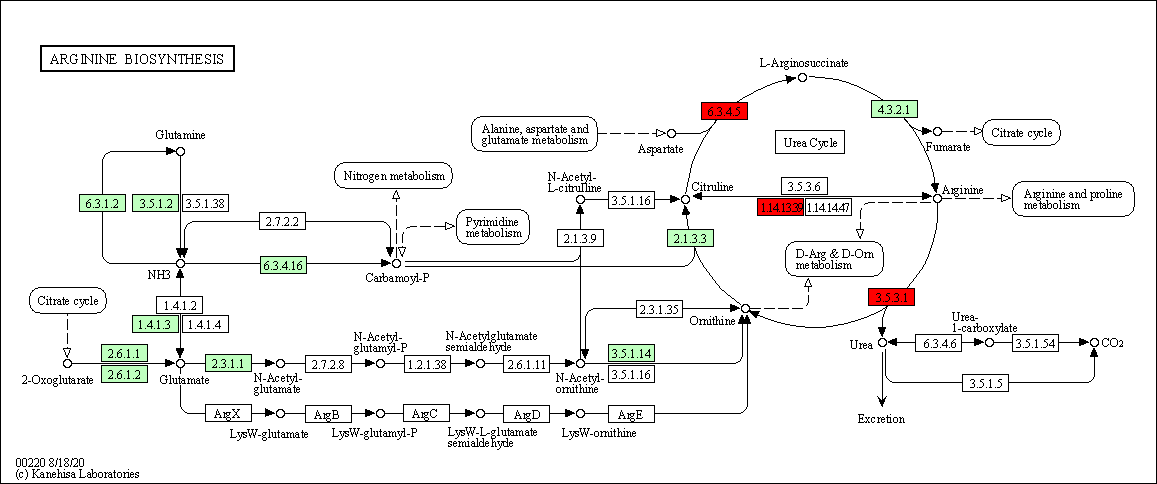

Supplement: Supplementary file 1 [file molecules-28-01606-s001.zip › raw data/KEGG/IL-1b_vs_N/DEG_pathway/mmu00220.png]

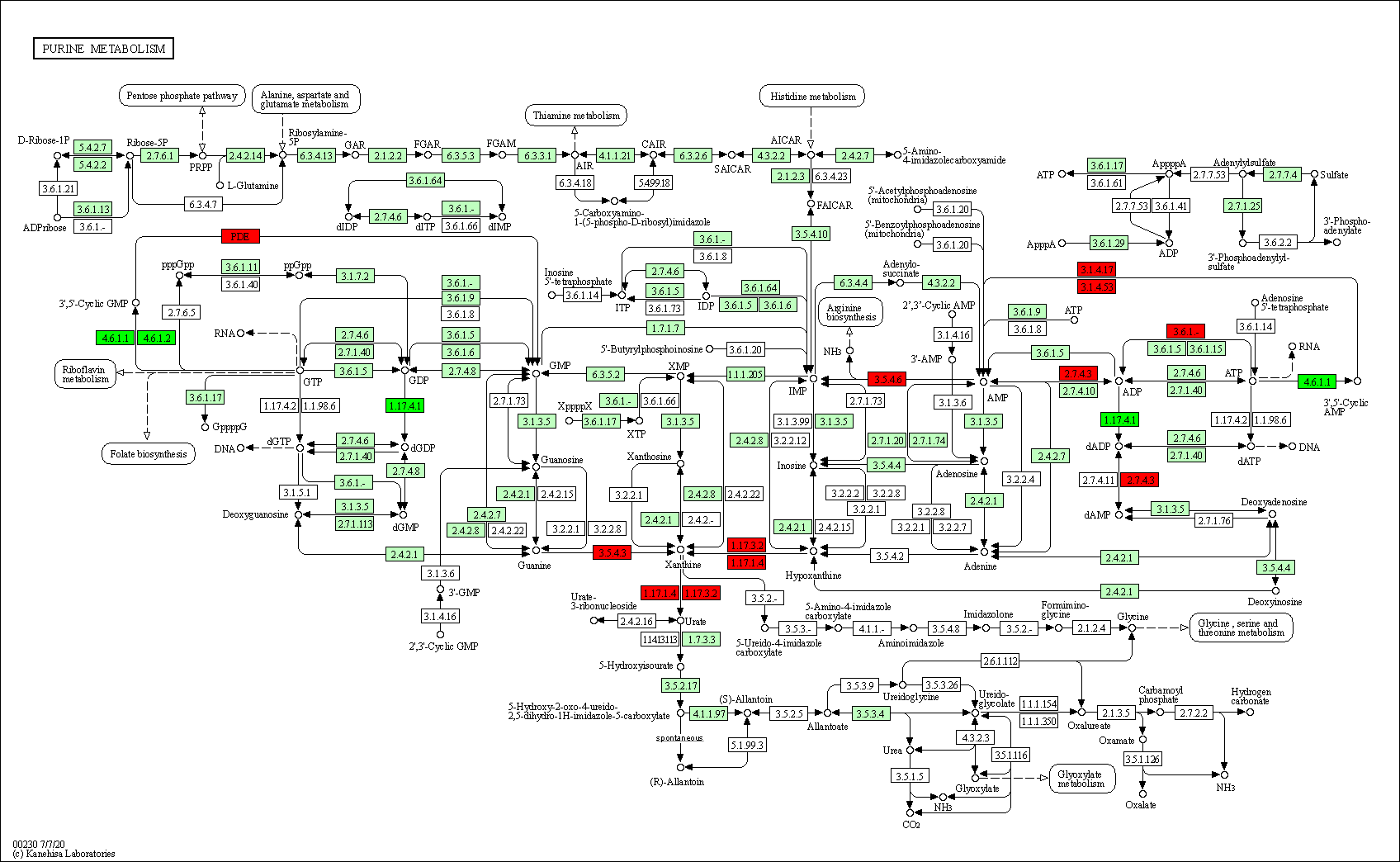

Supplement: Supplementary file 1 [file molecules-28-01606-s001.zip › raw data/KEGG/IL-1b_vs_N/DEG_pathway/mmu00230.png]

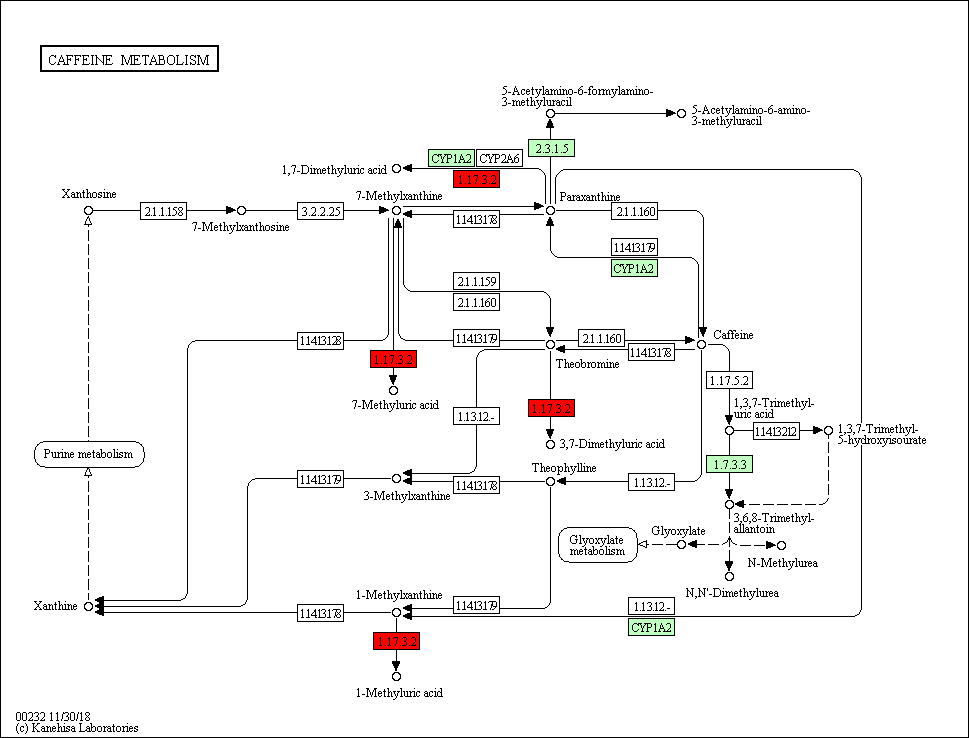

Supplement: Supplementary file 1 [file molecules-28-01606-s001.zip › raw data/KEGG/IL-1b_vs_N/DEG_pathway/mmu00232.png]

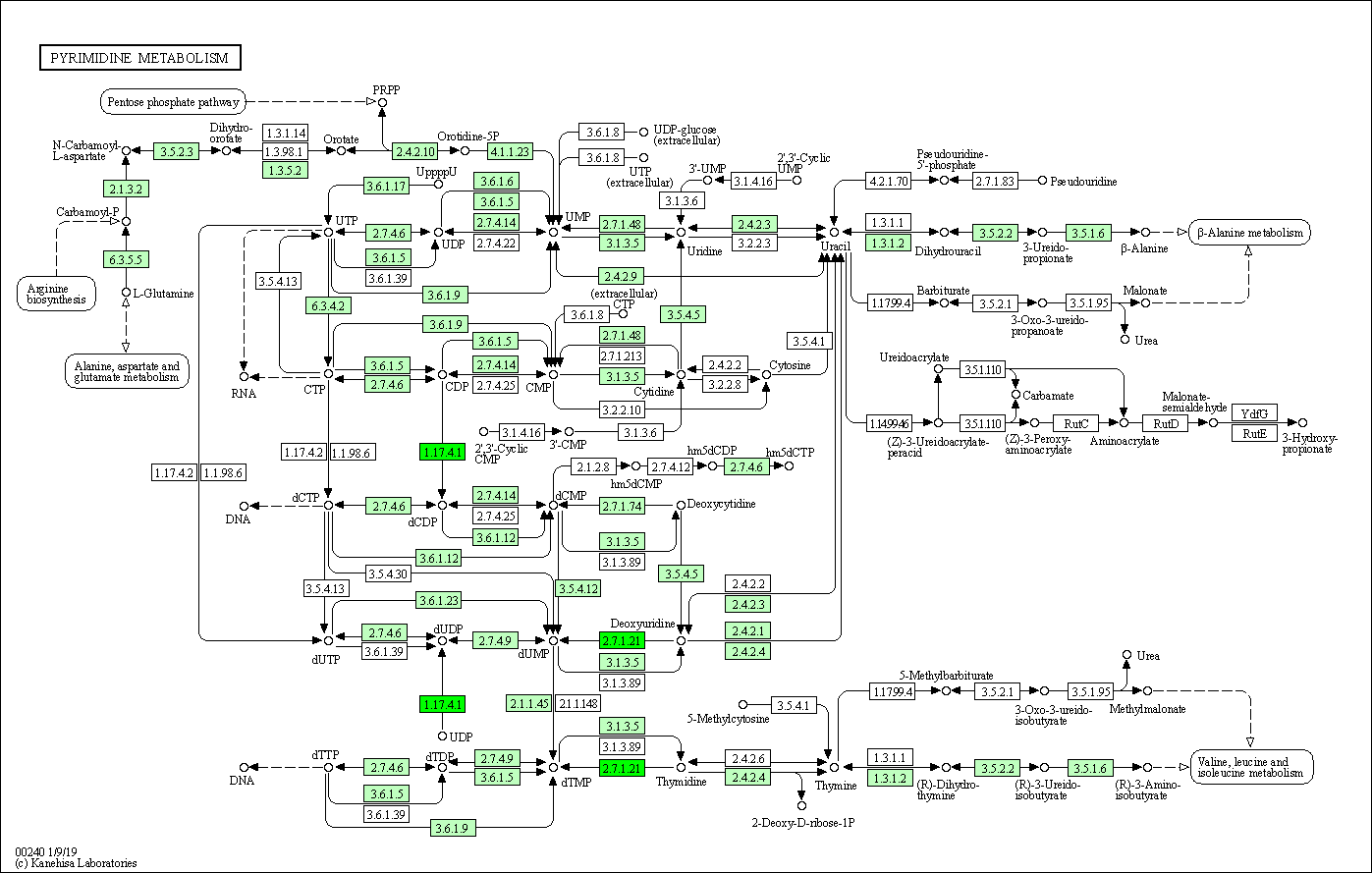

Supplement: Supplementary file 1 [file molecules-28-01606-s001.zip › raw data/KEGG/IL-1b_vs_N/DEG_pathway/mmu00240.png]

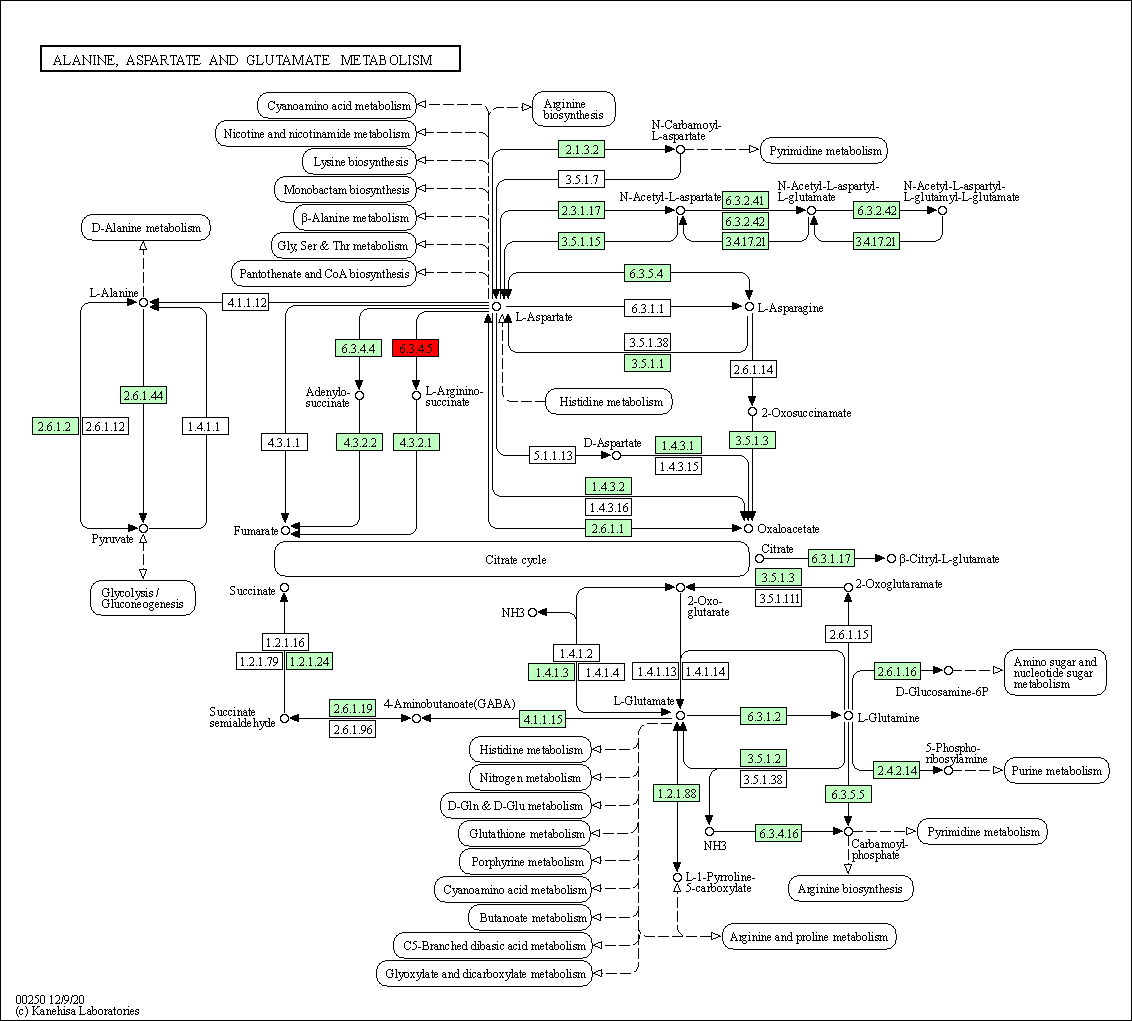

Supplement: Supplementary file 1 [file molecules-28-01606-s001.zip › raw data/KEGG/IL-1b_vs_N/DEG_pathway/mmu00250.png]

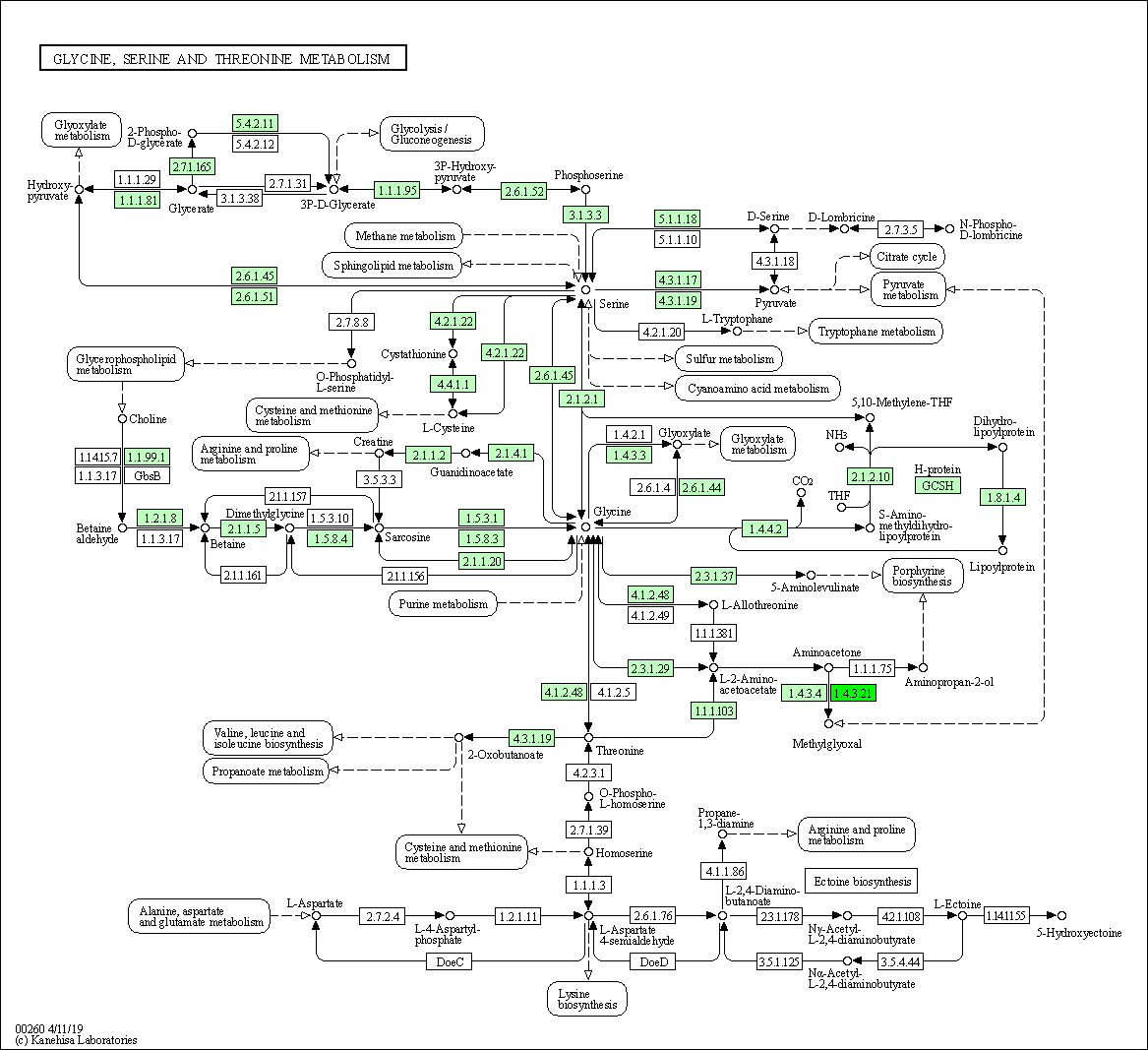

Supplement: Supplementary file 1 [file molecules-28-01606-s001.zip › raw data/KEGG/IL-1b_vs_N/DEG_pathway/mmu00260.png]

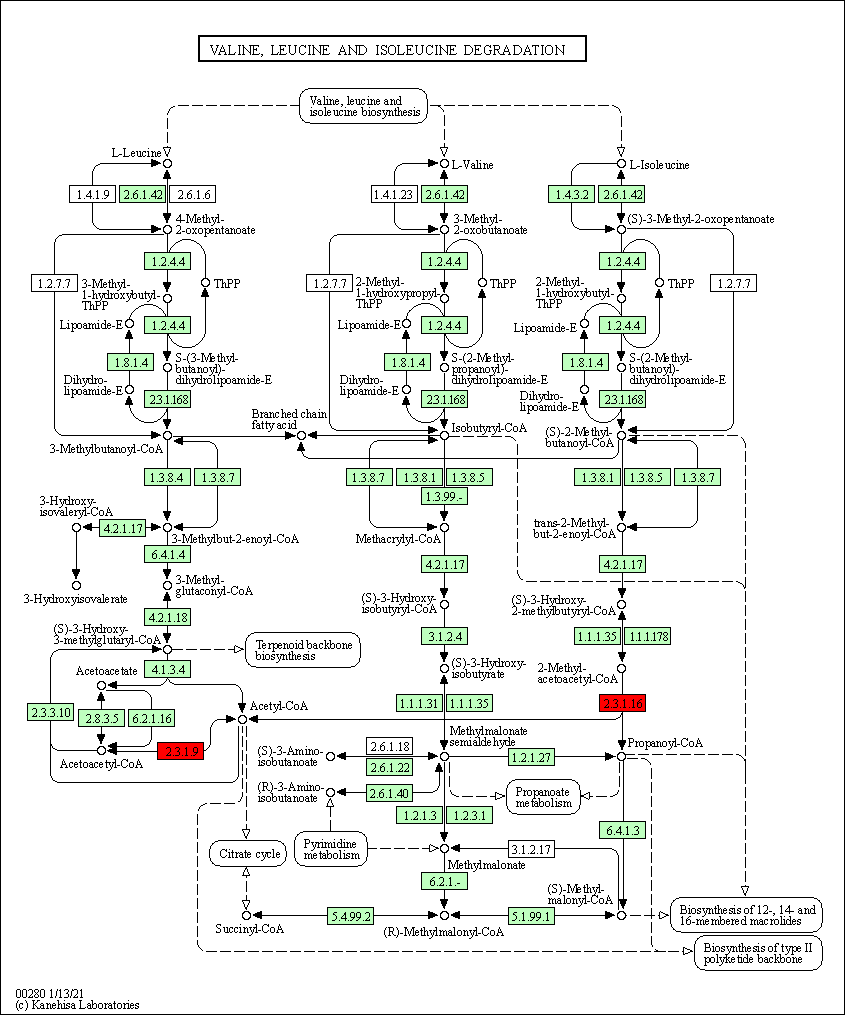

Supplement: Supplementary file 1 [file molecules-28-01606-s001.zip › raw data/KEGG/IL-1b_vs_N/DEG_pathway/mmu00280.png]

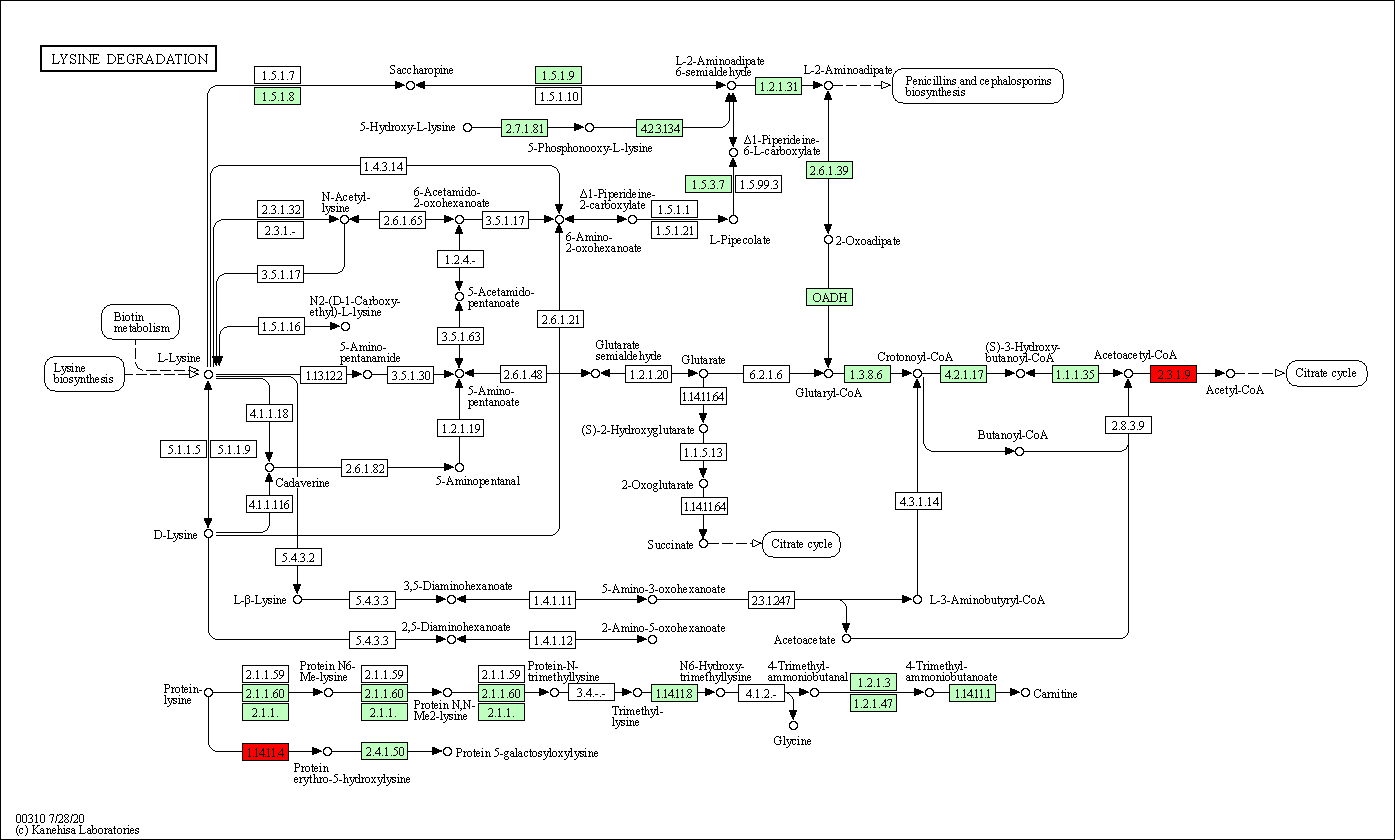

Supplement: Supplementary file 1 [file molecules-28-01606-s001.zip › raw data/KEGG/IL-1b_vs_N/DEG_pathway/mmu00310.png]

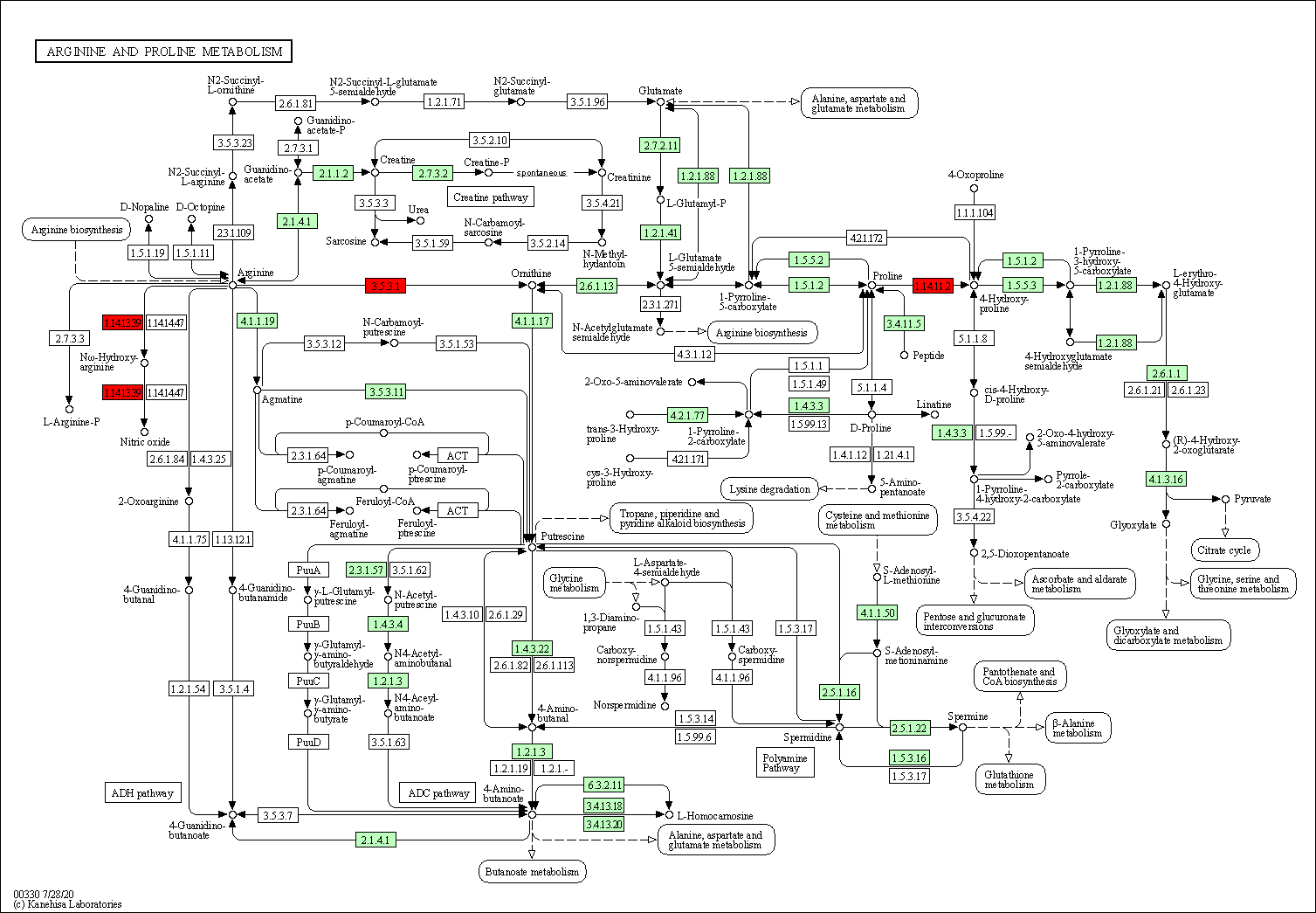

Supplement: Supplementary file 1 [file molecules-28-01606-s001.zip › raw data/KEGG/IL-1b_vs_N/DEG_pathway/mmu00330.png]

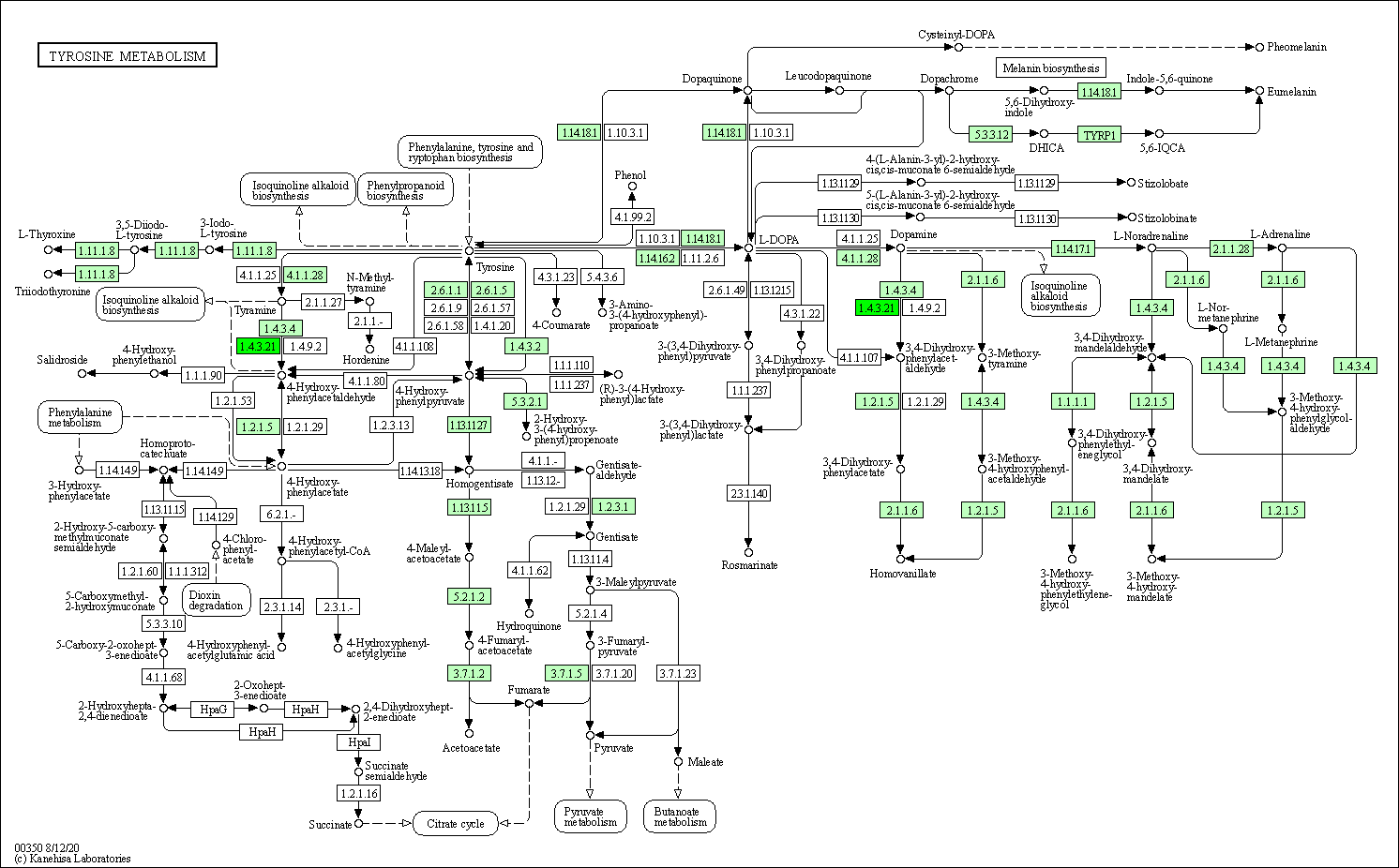

Supplement: Supplementary file 1 [file molecules-28-01606-s001.zip › raw data/KEGG/IL-1b_vs_N/DEG_pathway/mmu00350.png]

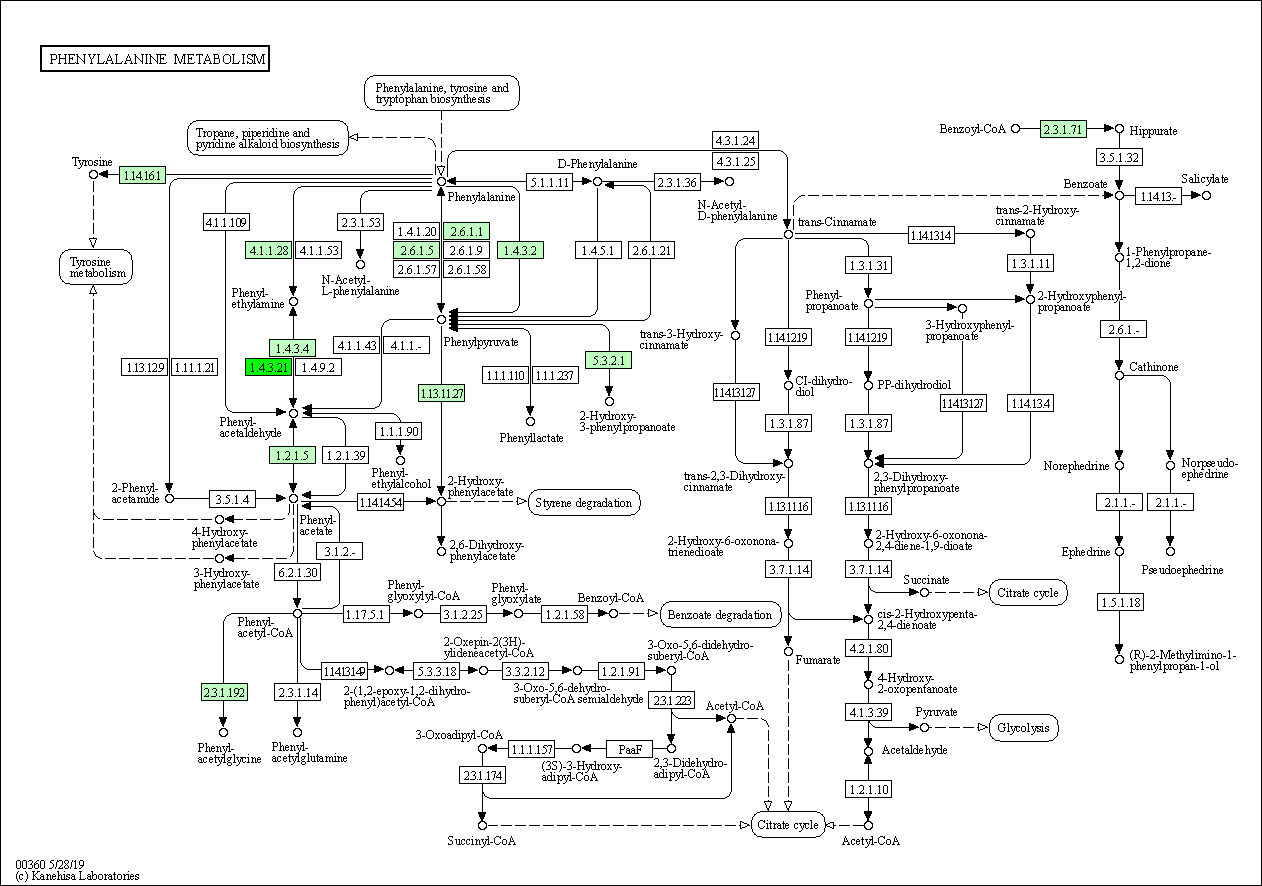

Supplement: Supplementary file 1 [file molecules-28-01606-s001.zip › raw data/KEGG/IL-1b_vs_N/DEG_pathway/mmu00360.png]

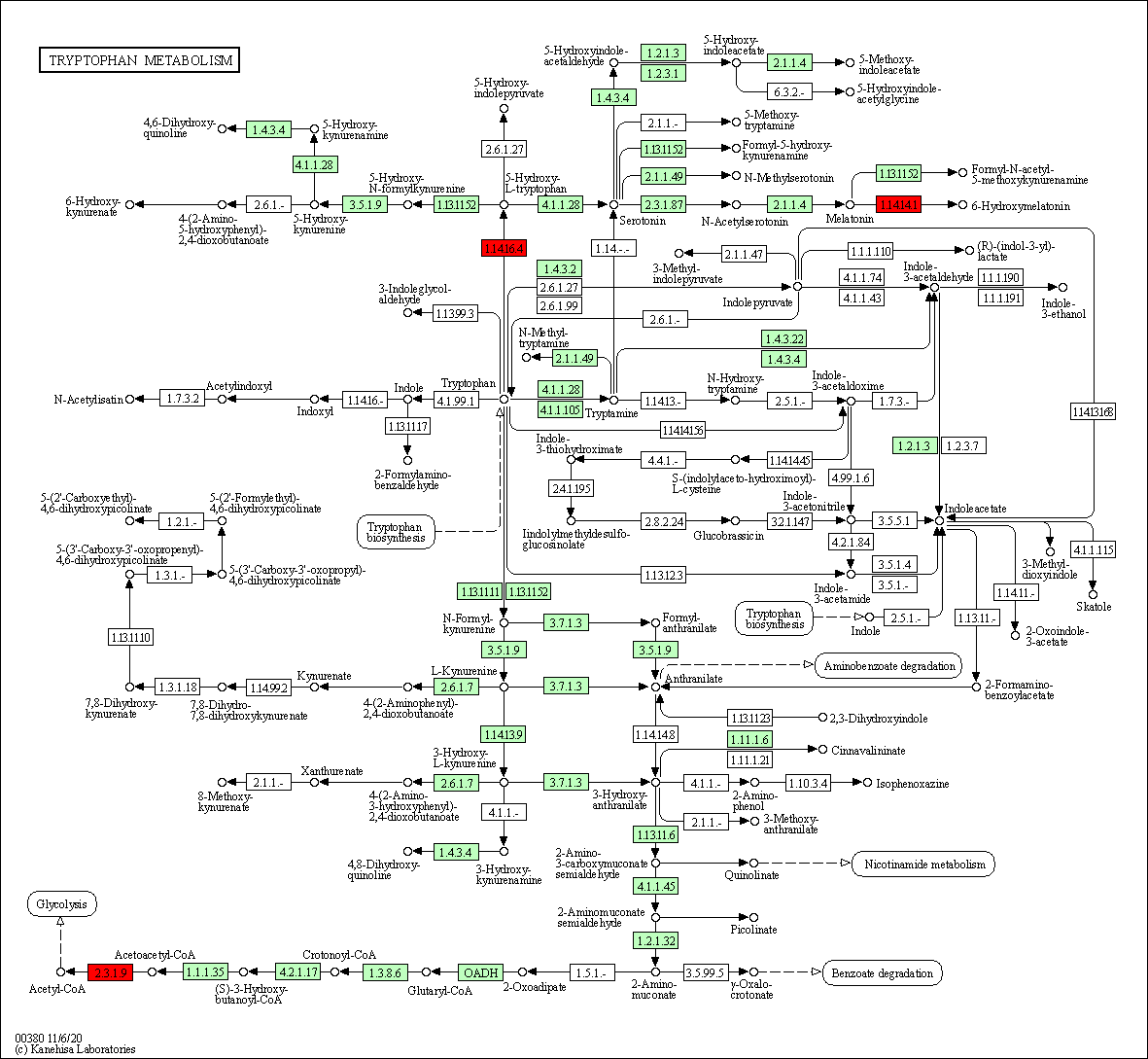

Supplement: Supplementary file 1 [file molecules-28-01606-s001.zip › raw data/KEGG/IL-1b_vs_N/DEG_pathway/mmu00380.png]

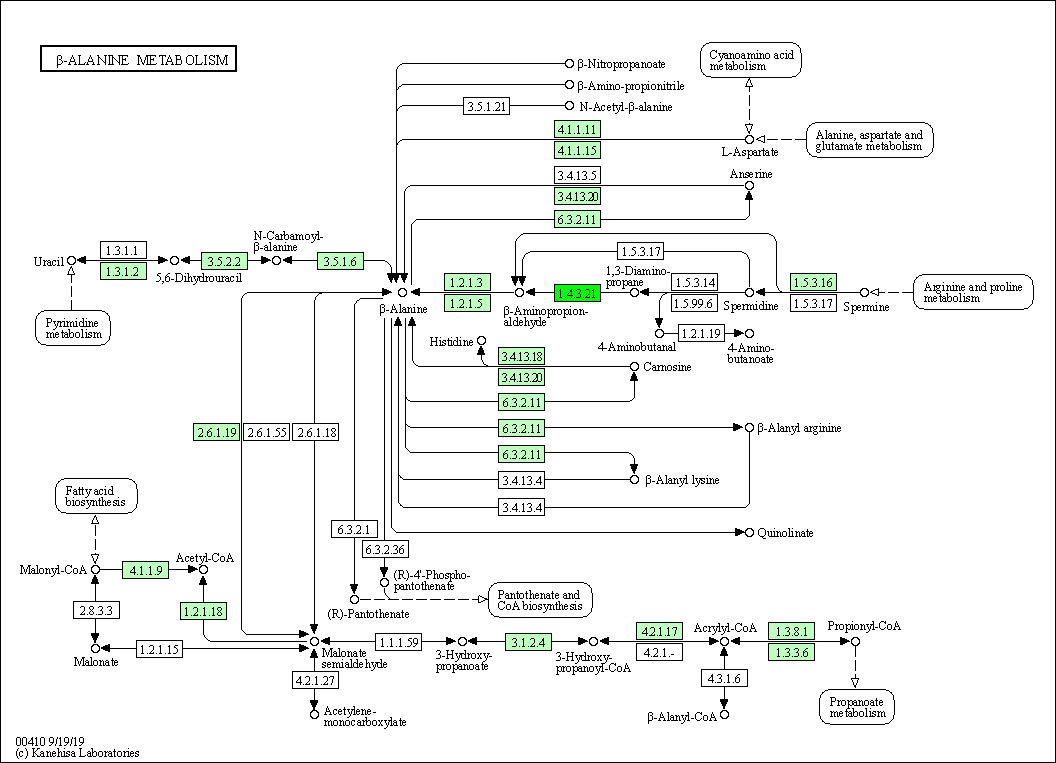

Supplement: Supplementary file 1 [file molecules-28-01606-s001.zip › raw data/KEGG/IL-1b_vs_N/DEG_pathway/mmu00410.png]

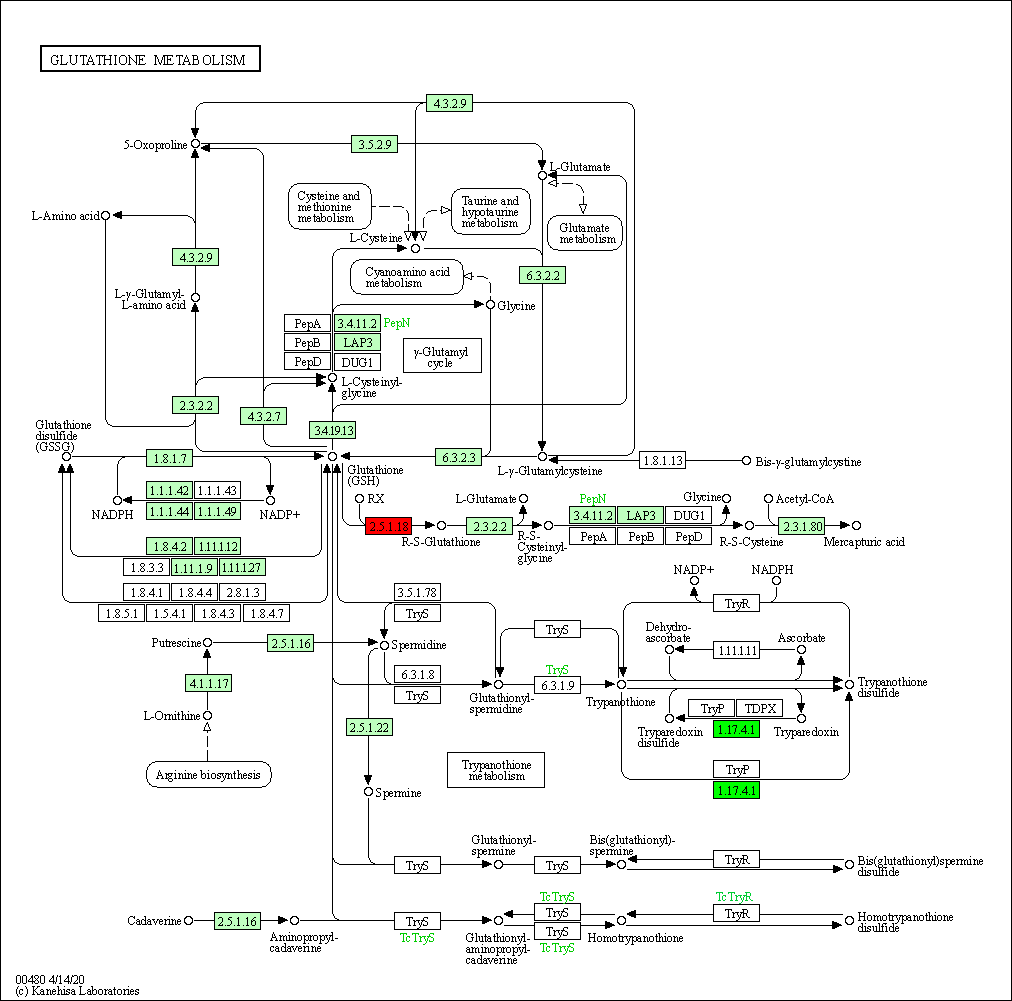

Supplement: Supplementary file 1 [file molecules-28-01606-s001.zip › raw data/KEGG/IL-1b_vs_N/DEG_pathway/mmu00480.png]

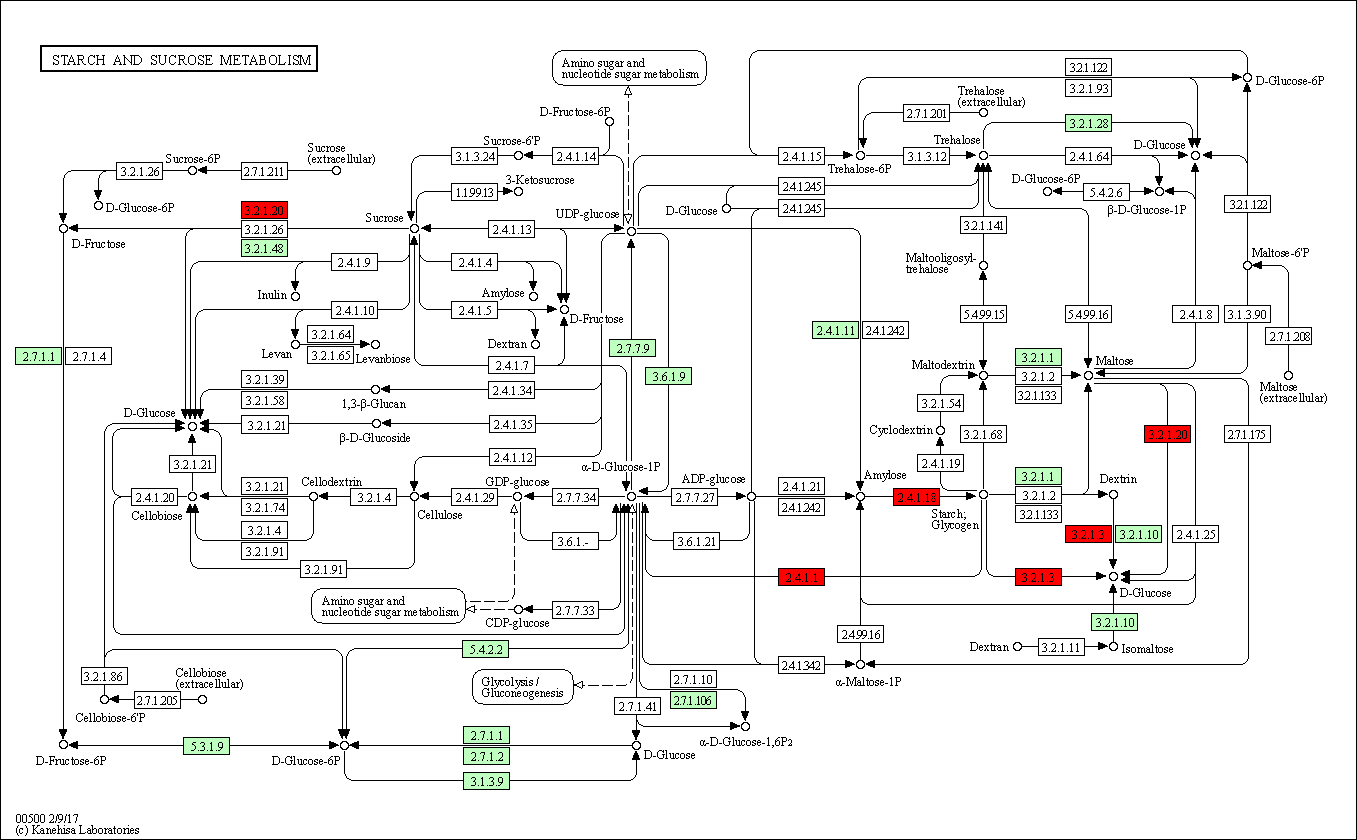

Supplement: Supplementary file 1 [file molecules-28-01606-s001.zip › raw data/KEGG/IL-1b_vs_N/DEG_pathway/mmu00500.png]

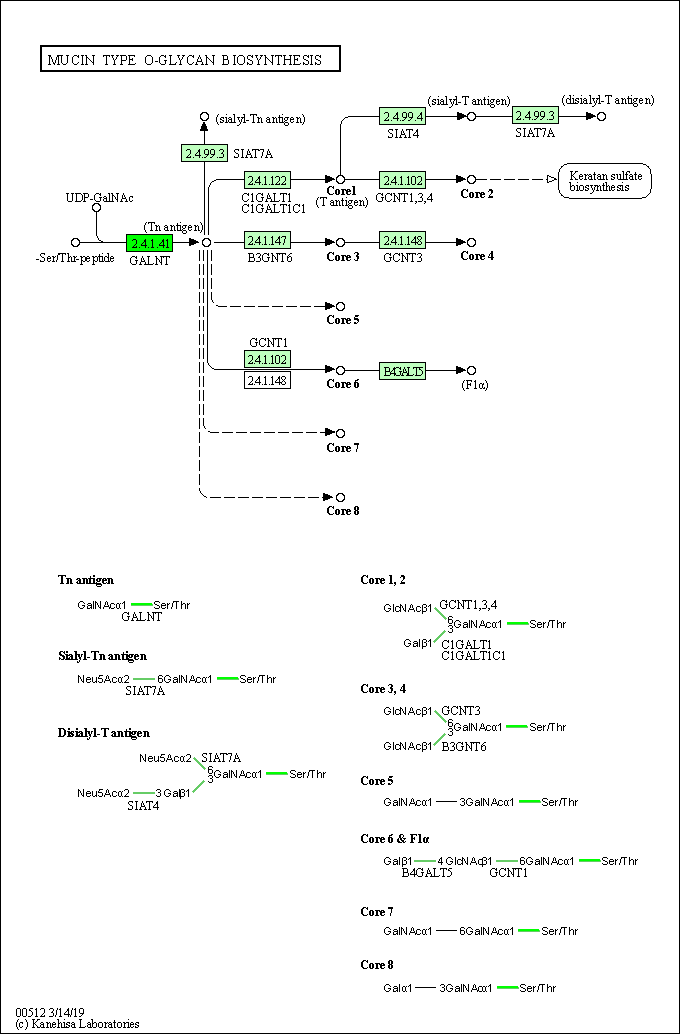

Supplement: Supplementary file 1 [file molecules-28-01606-s001.zip › raw data/KEGG/IL-1b_vs_N/DEG_pathway/mmu00512.png]

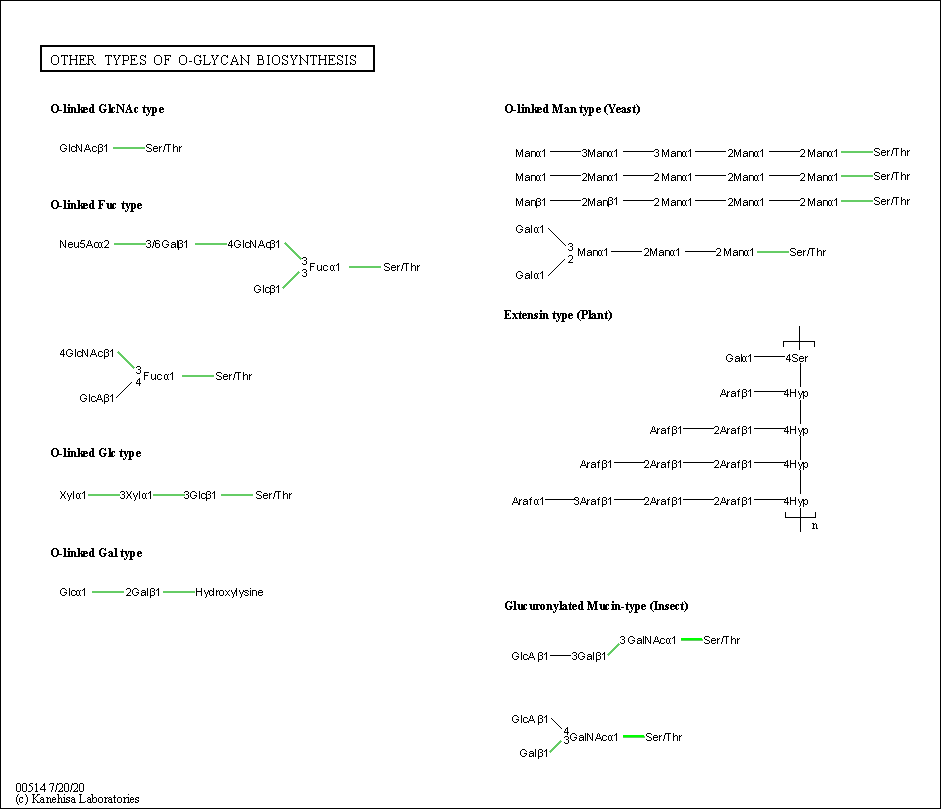

Supplement: Supplementary file 1 [file molecules-28-01606-s001.zip › raw data/KEGG/IL-1b_vs_N/DEG_pathway/mmu00514.png]

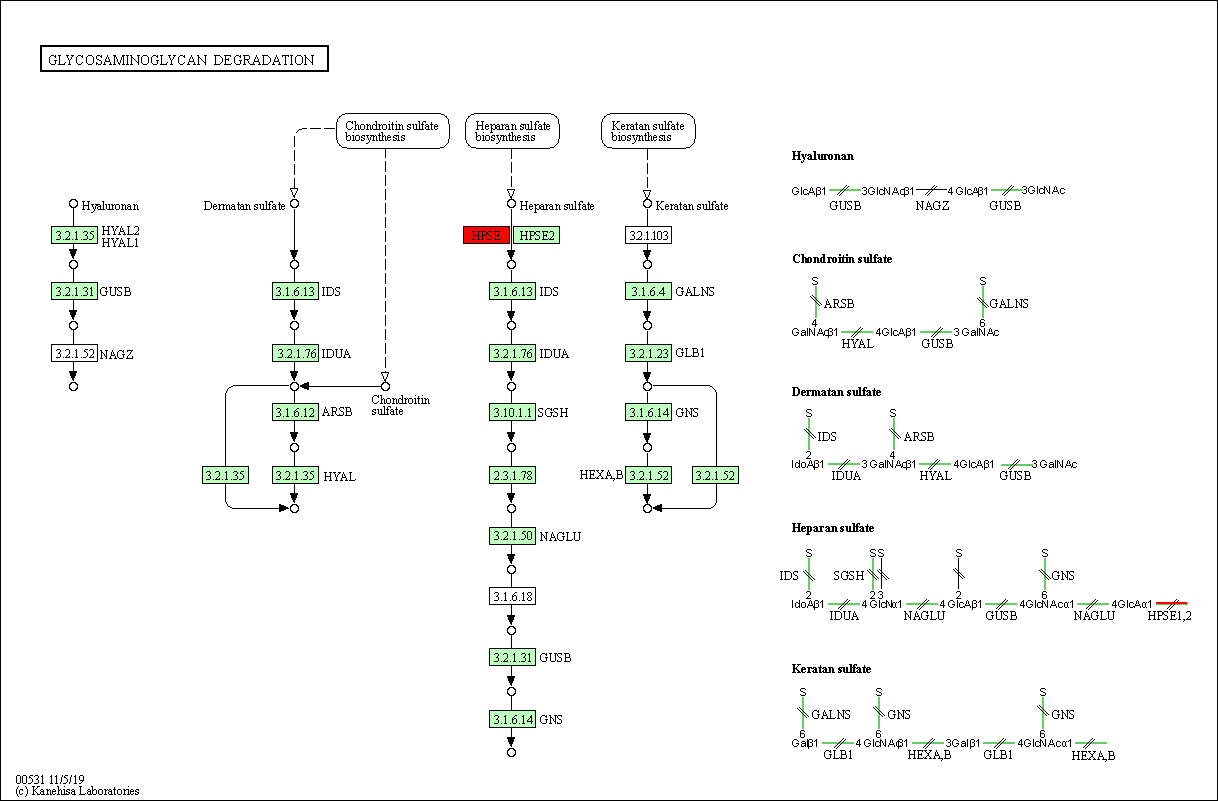

Supplement: Supplementary file 1 [file molecules-28-01606-s001.zip › raw data/KEGG/IL-1b_vs_N/DEG_pathway/mmu00531.png]

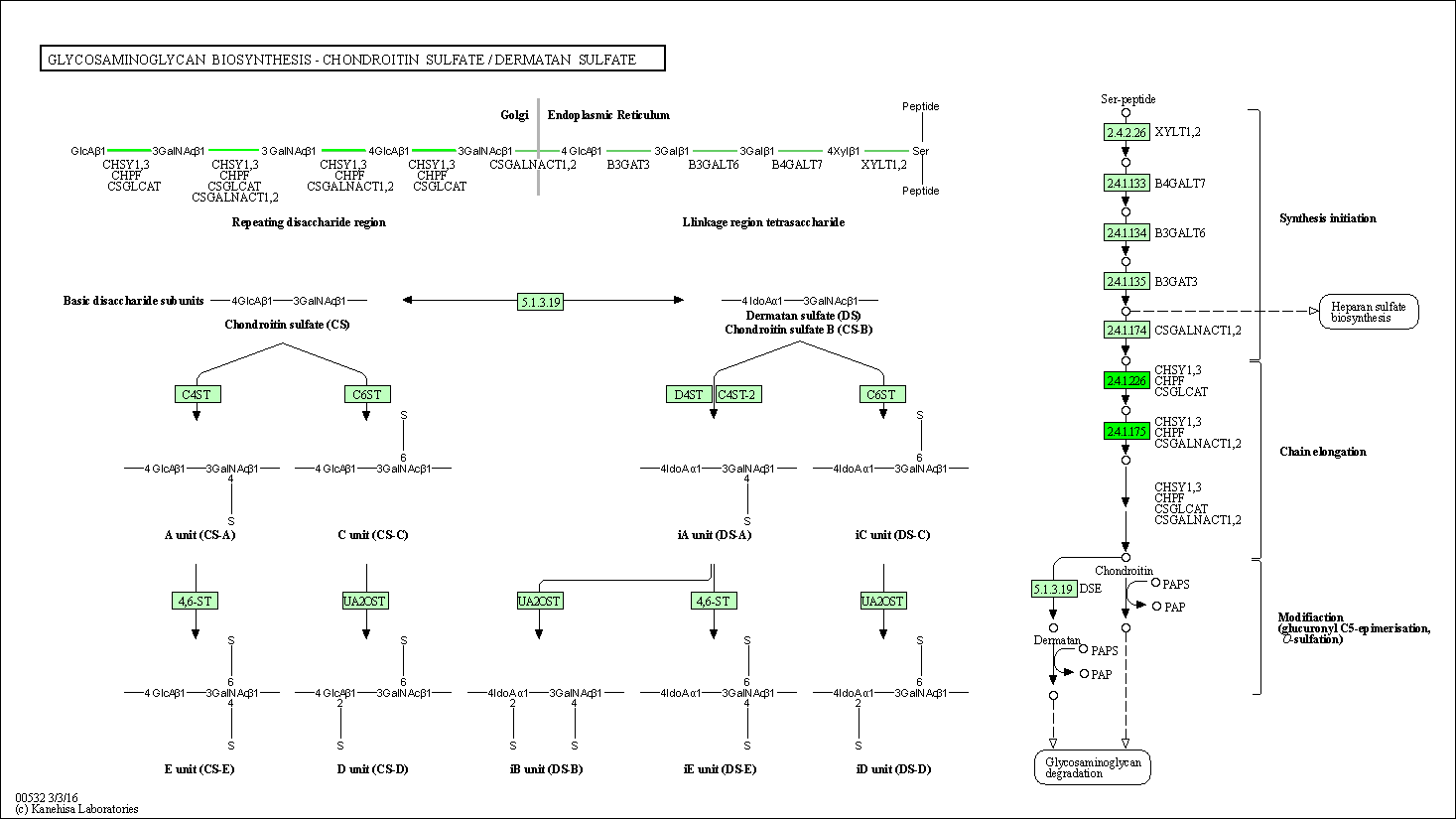

Supplement: Supplementary file 1 [file molecules-28-01606-s001.zip › raw data/KEGG/IL-1b_vs_N/DEG_pathway/mmu00532.png]

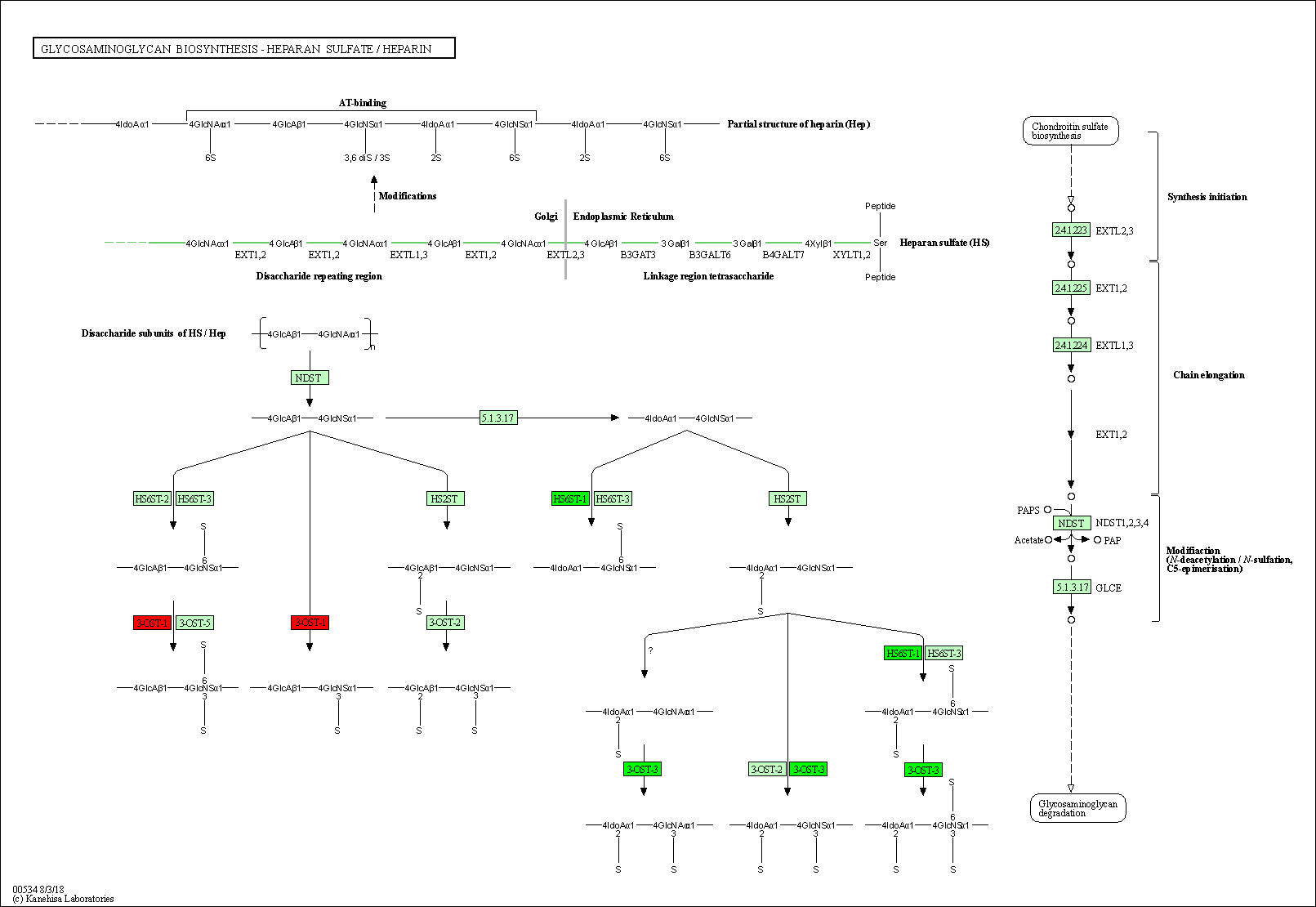

Supplement: Supplementary file 1 [file molecules-28-01606-s001.zip › raw data/KEGG/IL-1b_vs_N/DEG_pathway/mmu00534.png]

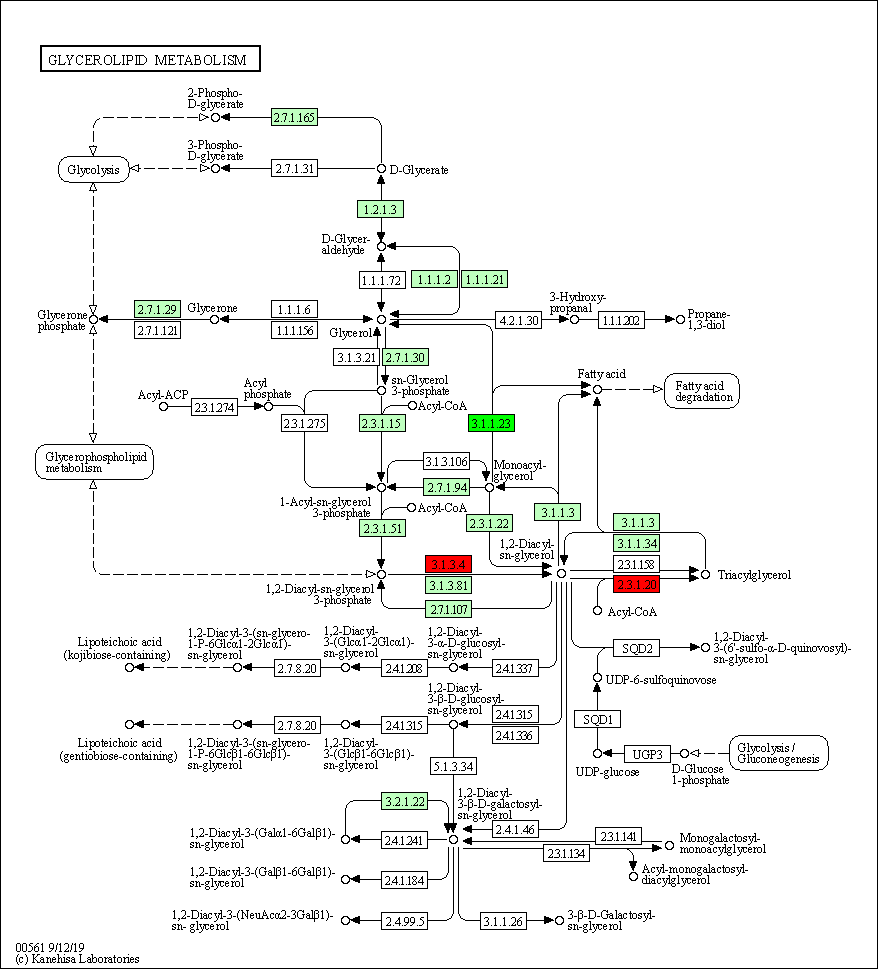

Supplement: Supplementary file 1 [file molecules-28-01606-s001.zip › raw data/KEGG/IL-1b_vs_N/DEG_pathway/mmu00561.png]

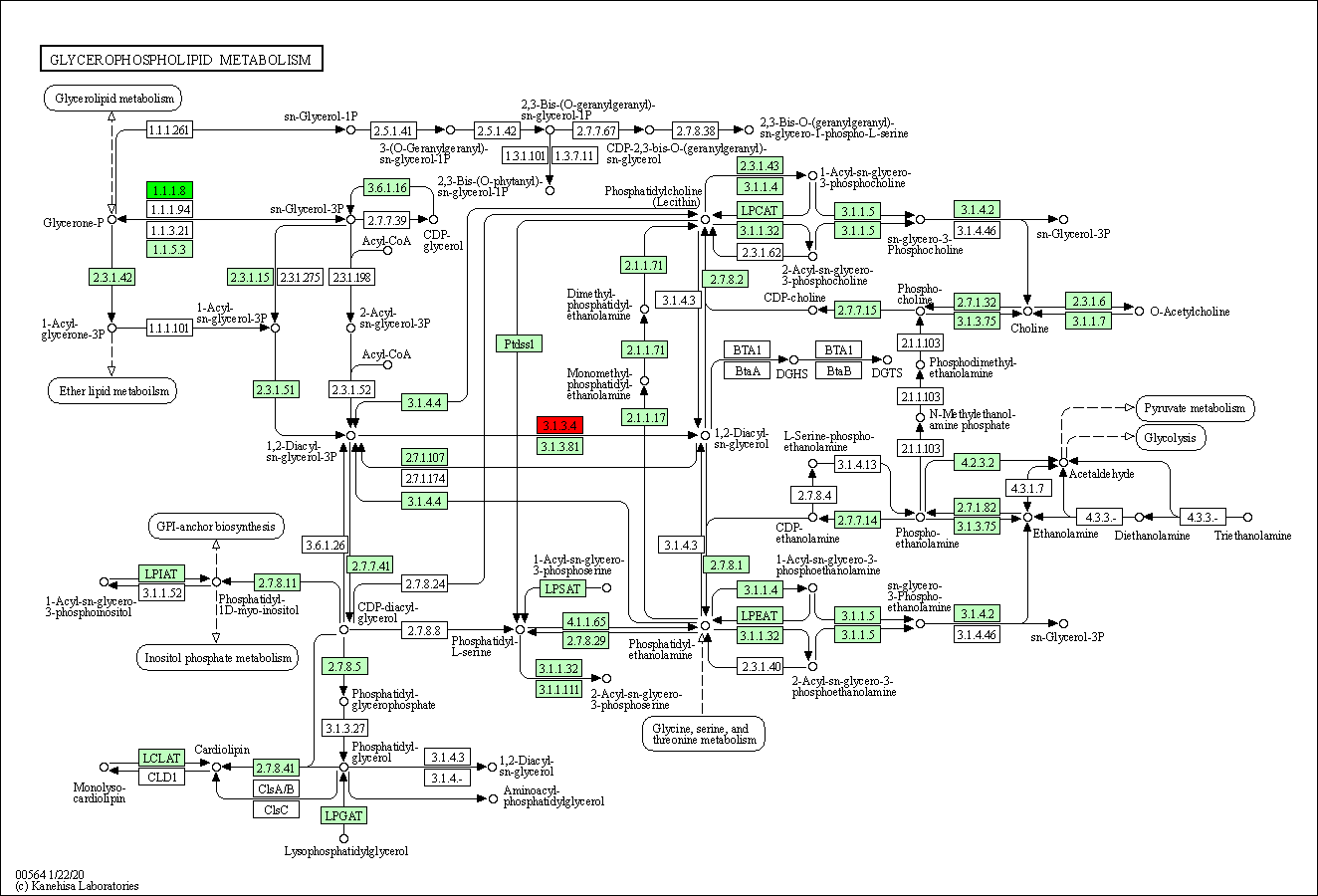

Supplement: Supplementary file 1 [file molecules-28-01606-s001.zip › raw data/KEGG/IL-1b_vs_N/DEG_pathway/mmu00564.png]

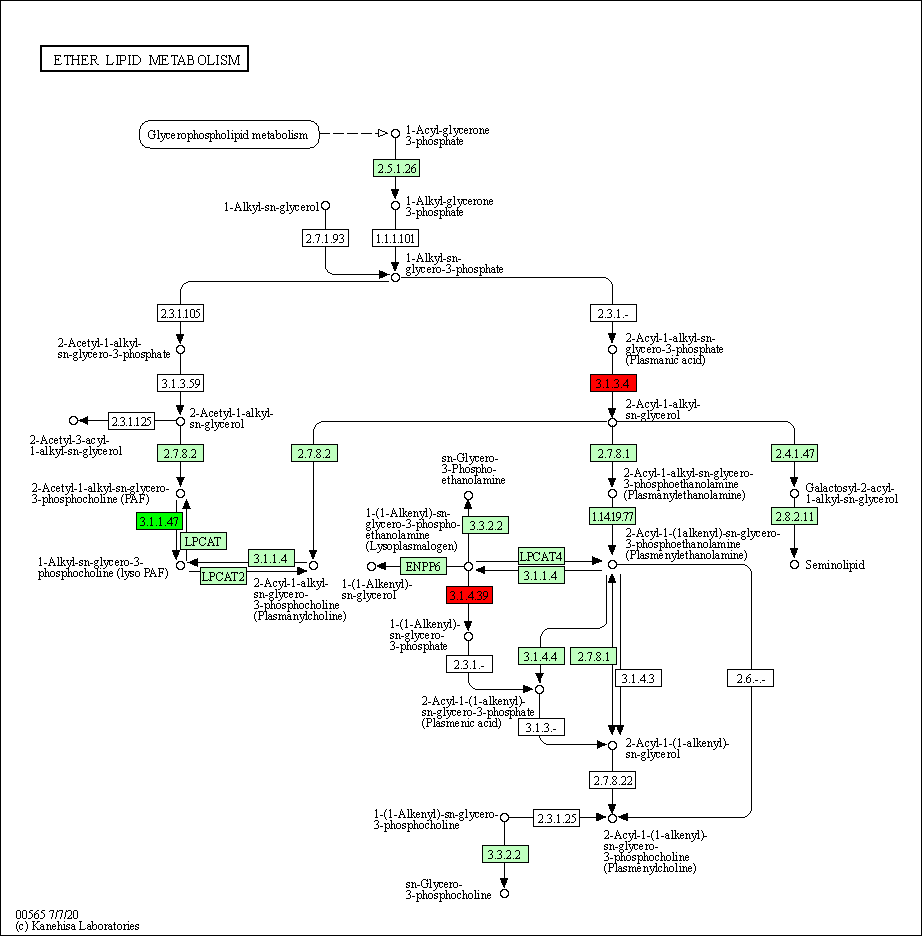

Supplement: Supplementary file 1 [file molecules-28-01606-s001.zip › raw data/KEGG/IL-1b_vs_N/DEG_pathway/mmu00565.png]

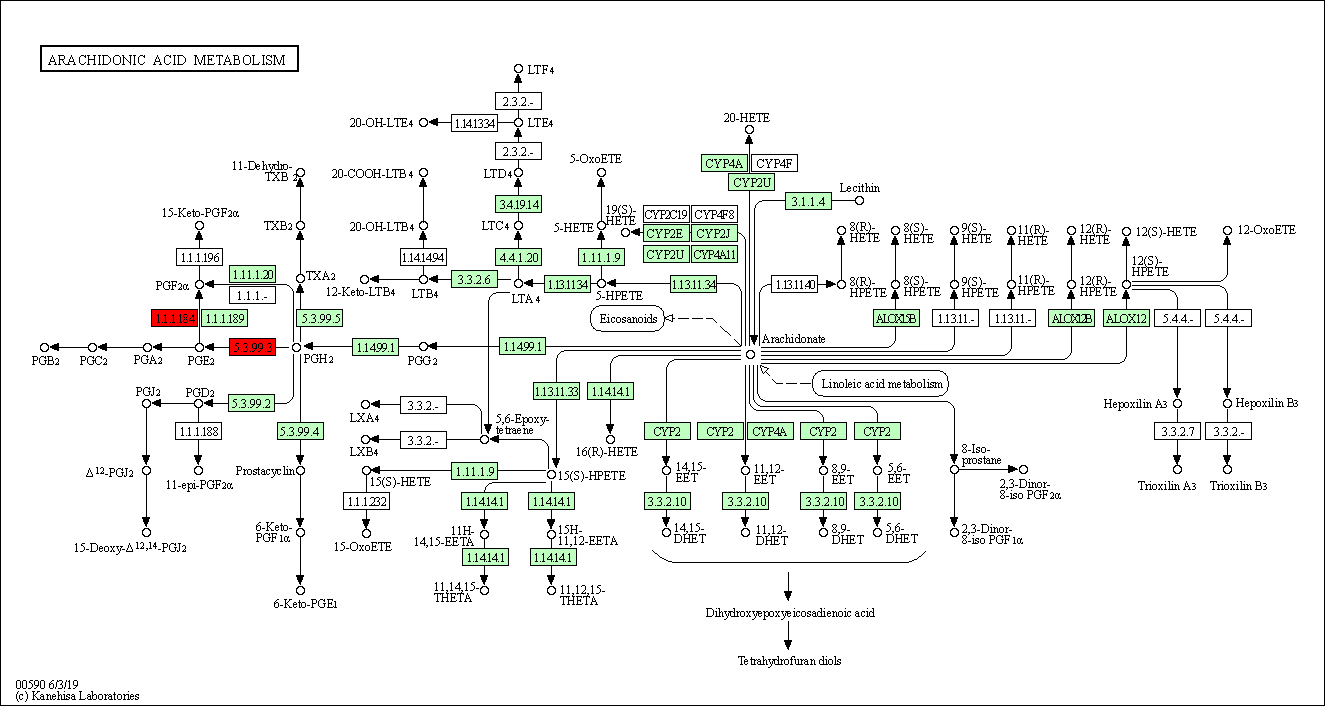

Supplement: Supplementary file 1 [file molecules-28-01606-s001.zip › raw data/KEGG/IL-1b_vs_N/DEG_pathway/mmu00590.png]

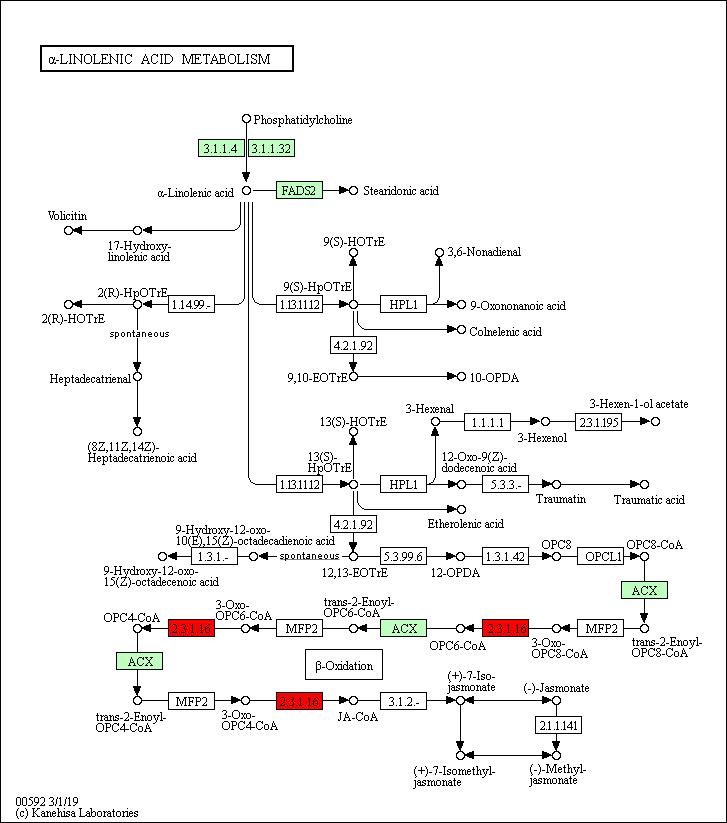

Supplement: Supplementary file 1 [file molecules-28-01606-s001.zip › raw data/KEGG/IL-1b_vs_N/DEG_pathway/mmu00592.png]

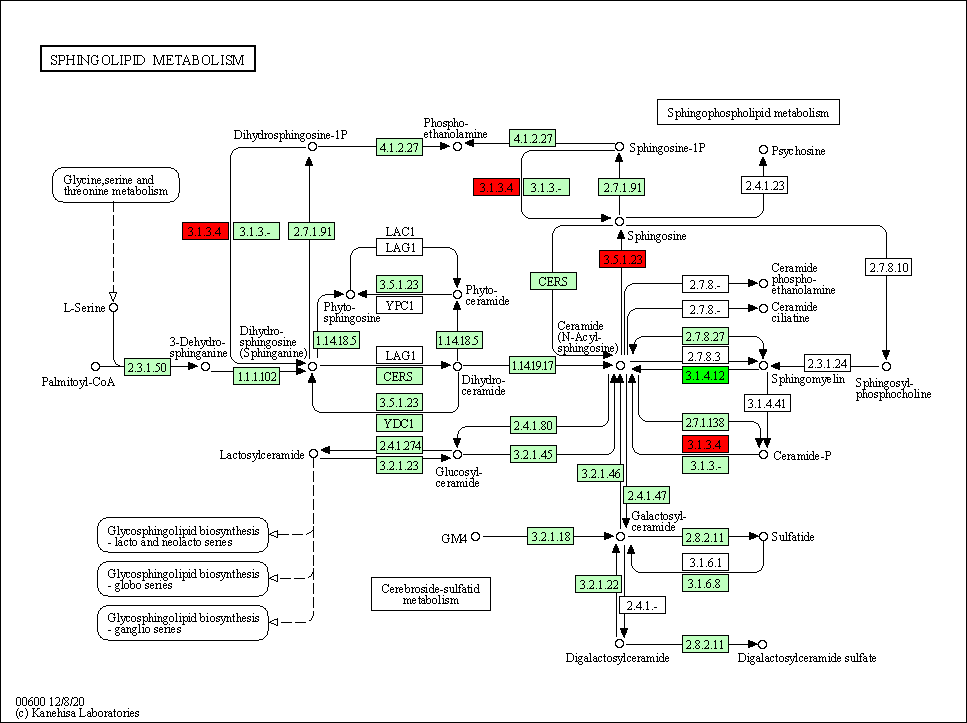

Supplement: Supplementary file 1 [file molecules-28-01606-s001.zip › raw data/KEGG/IL-1b_vs_N/DEG_pathway/mmu00600.png]

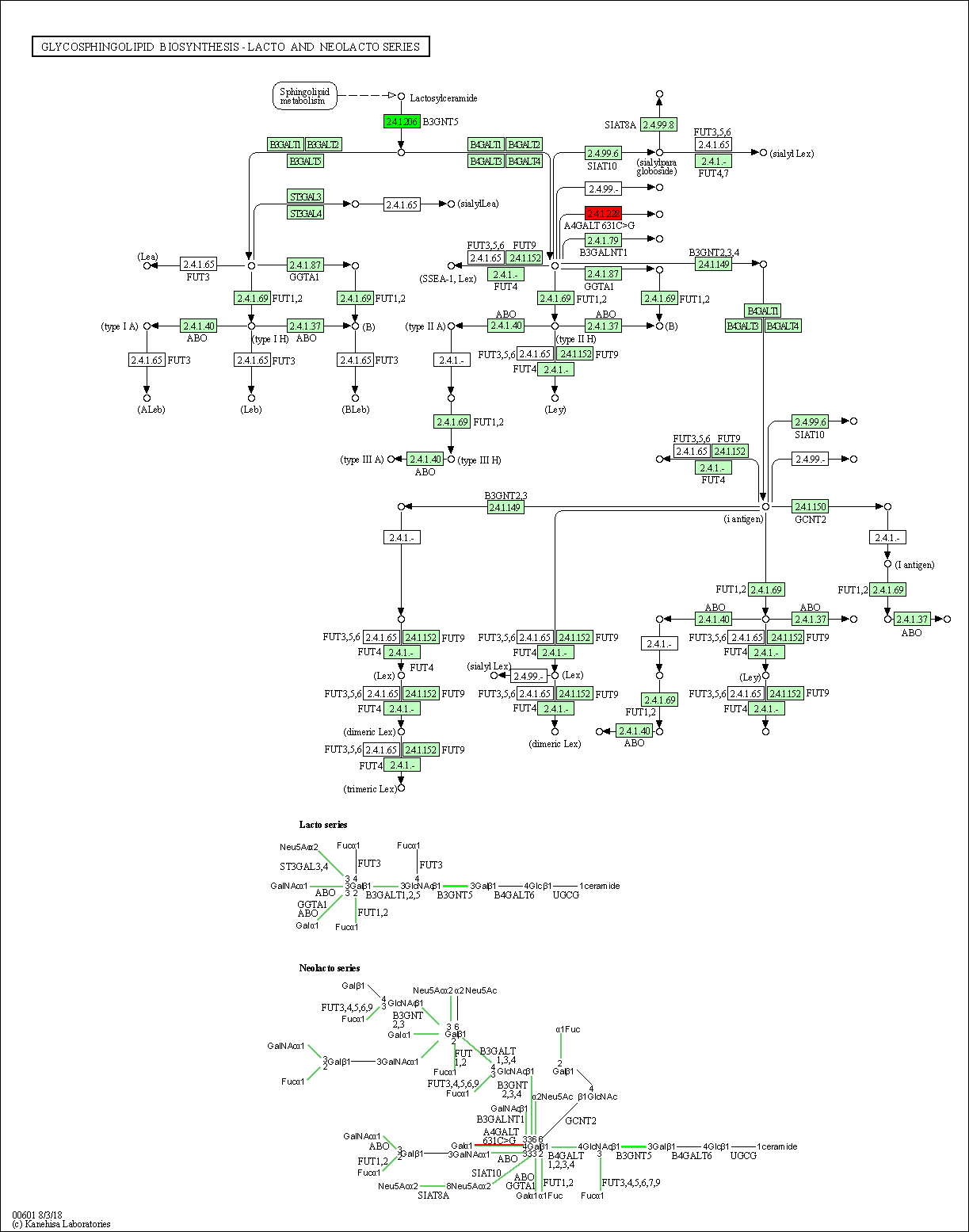

Supplement: Supplementary file 1 [file molecules-28-01606-s001.zip › raw data/KEGG/IL-1b_vs_N/DEG_pathway/mmu00601.png]

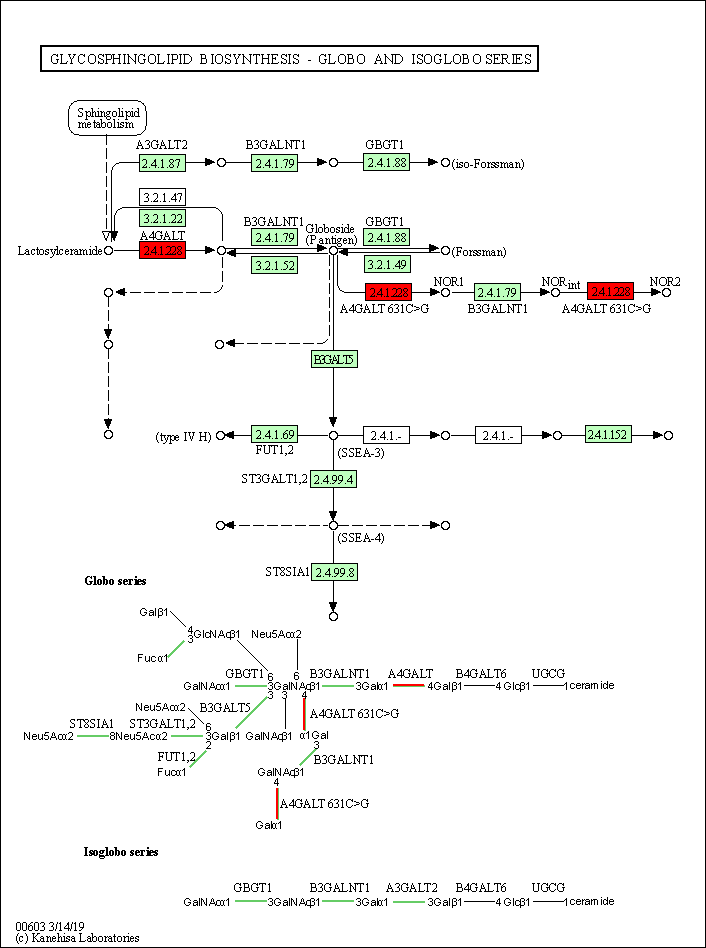

Supplement: Supplementary file 1 [file molecules-28-01606-s001.zip › raw data/KEGG/IL-1b_vs_N/DEG_pathway/mmu00603.png]

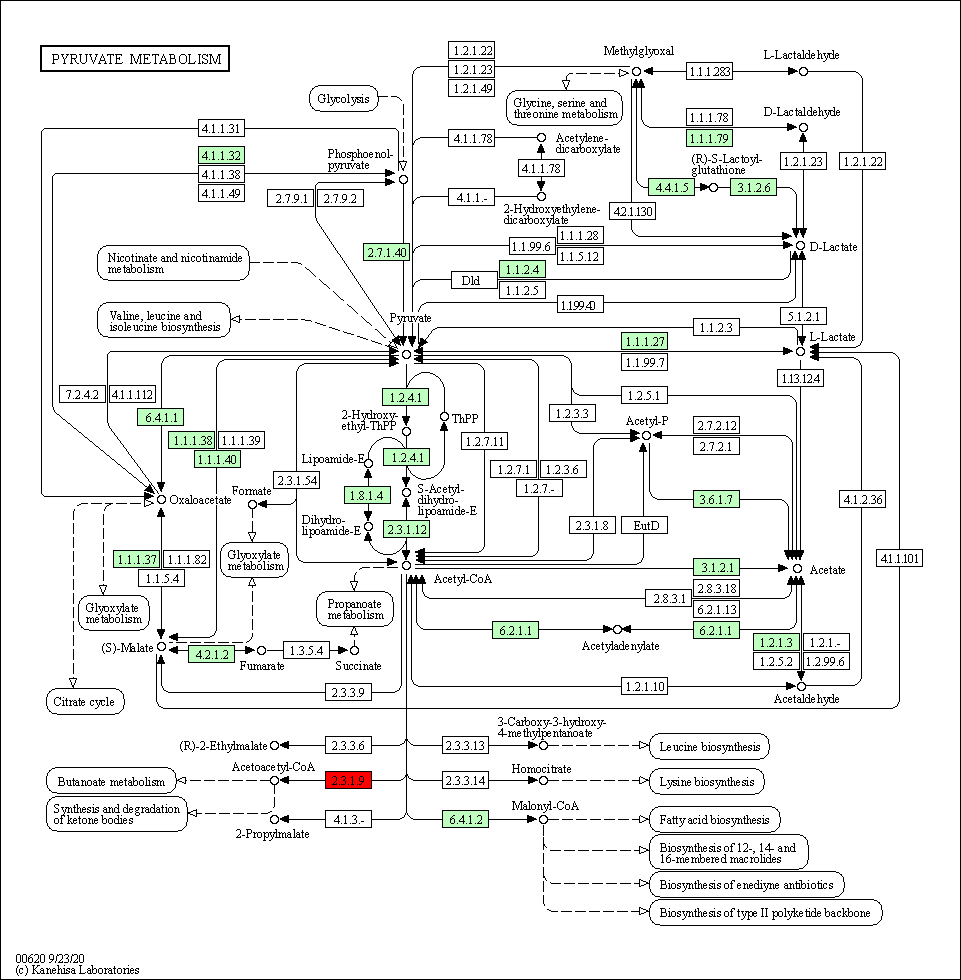

Supplement: Supplementary file 1 [file molecules-28-01606-s001.zip › raw data/KEGG/IL-1b_vs_N/DEG_pathway/mmu00620.png]

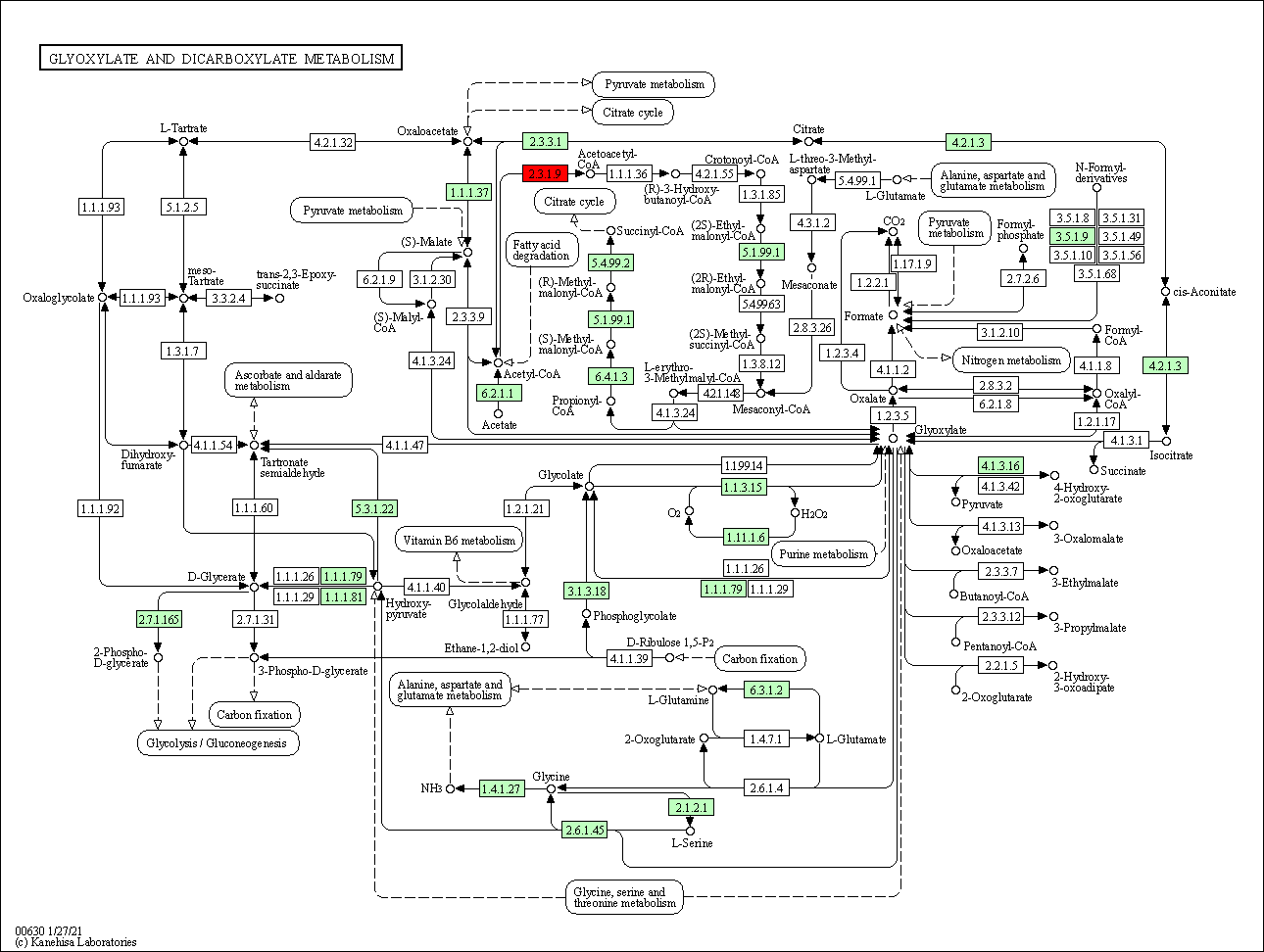

Supplement: Supplementary file 1 [file molecules-28-01606-s001.zip › raw data/KEGG/IL-1b_vs_N/DEG_pathway/mmu00630.png]

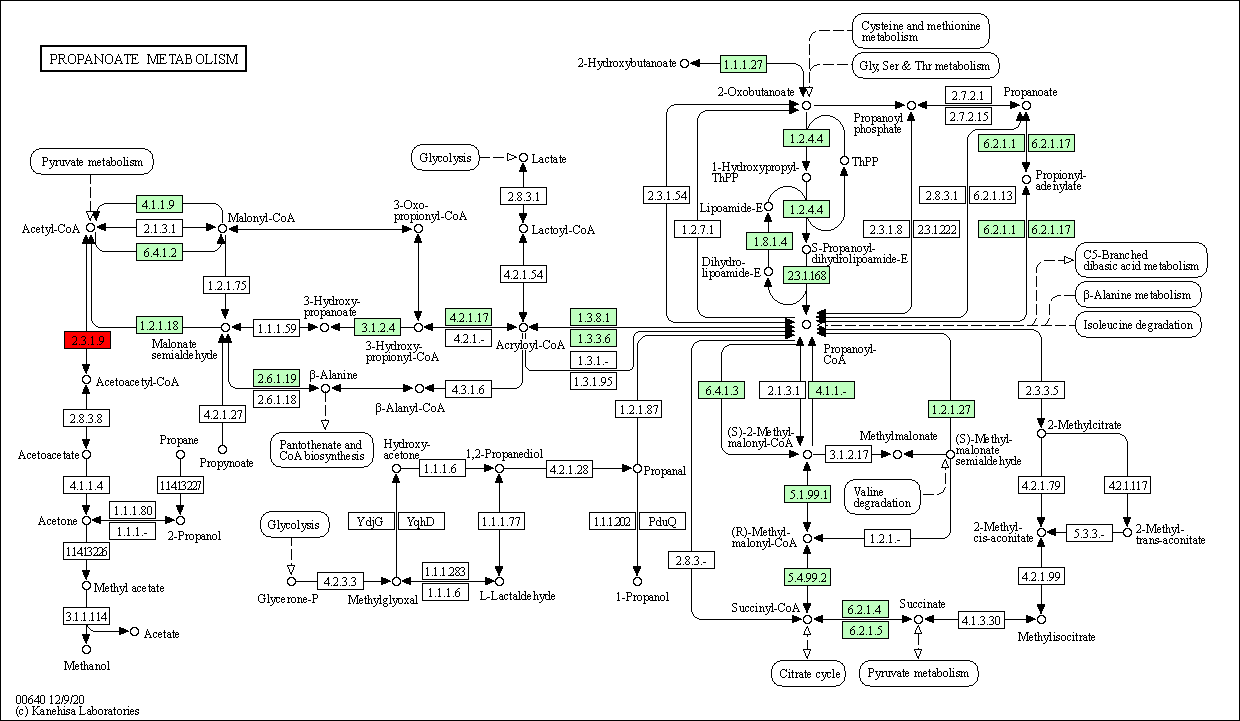

Supplement: Supplementary file 1 [file molecules-28-01606-s001.zip › raw data/KEGG/IL-1b_vs_N/DEG_pathway/mmu00640.png]

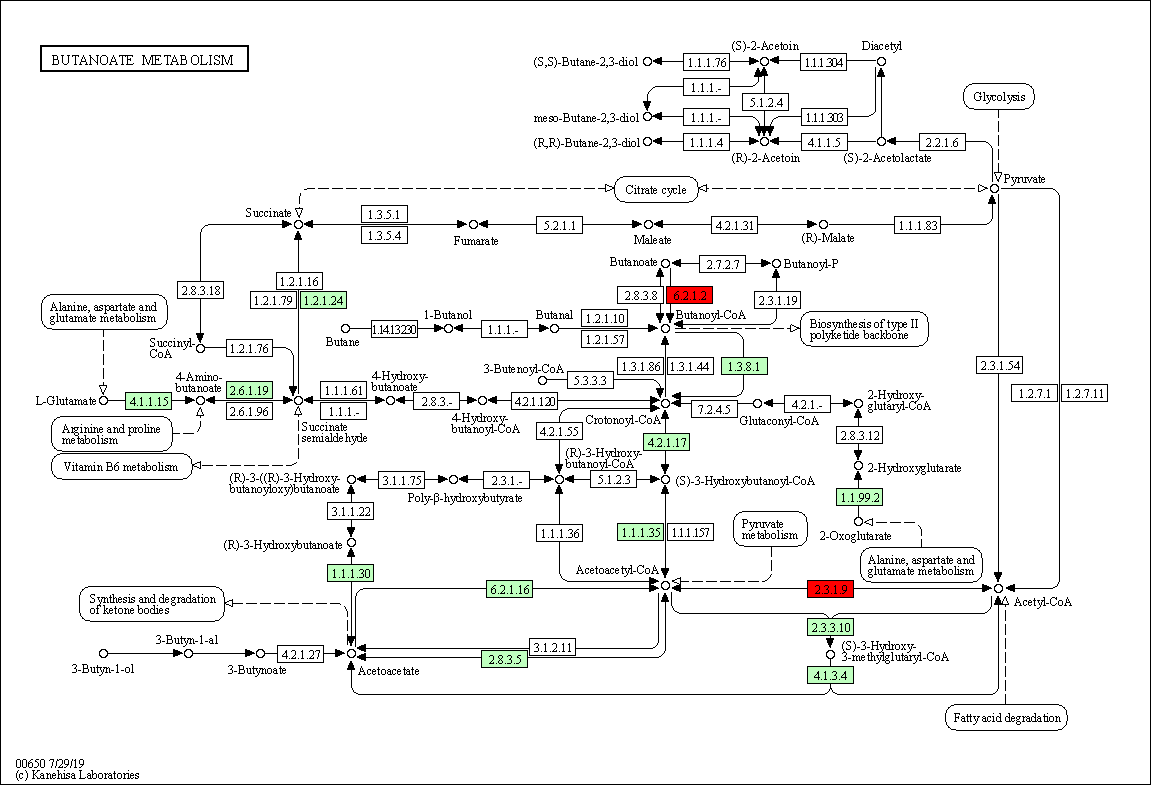

Supplement: Supplementary file 1 [file molecules-28-01606-s001.zip › raw data/KEGG/IL-1b_vs_N/DEG_pathway/mmu00650.png]

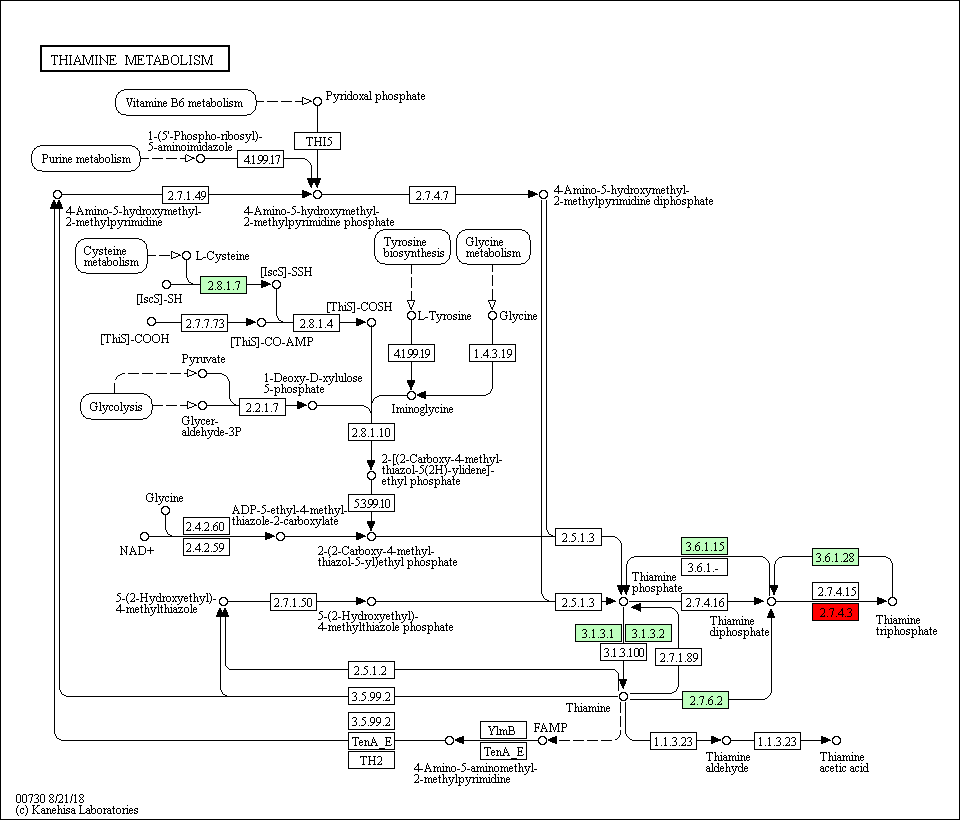

Supplement: Supplementary file 1 [file molecules-28-01606-s001.zip › raw data/KEGG/IL-1b_vs_N/DEG_pathway/mmu00730.png]

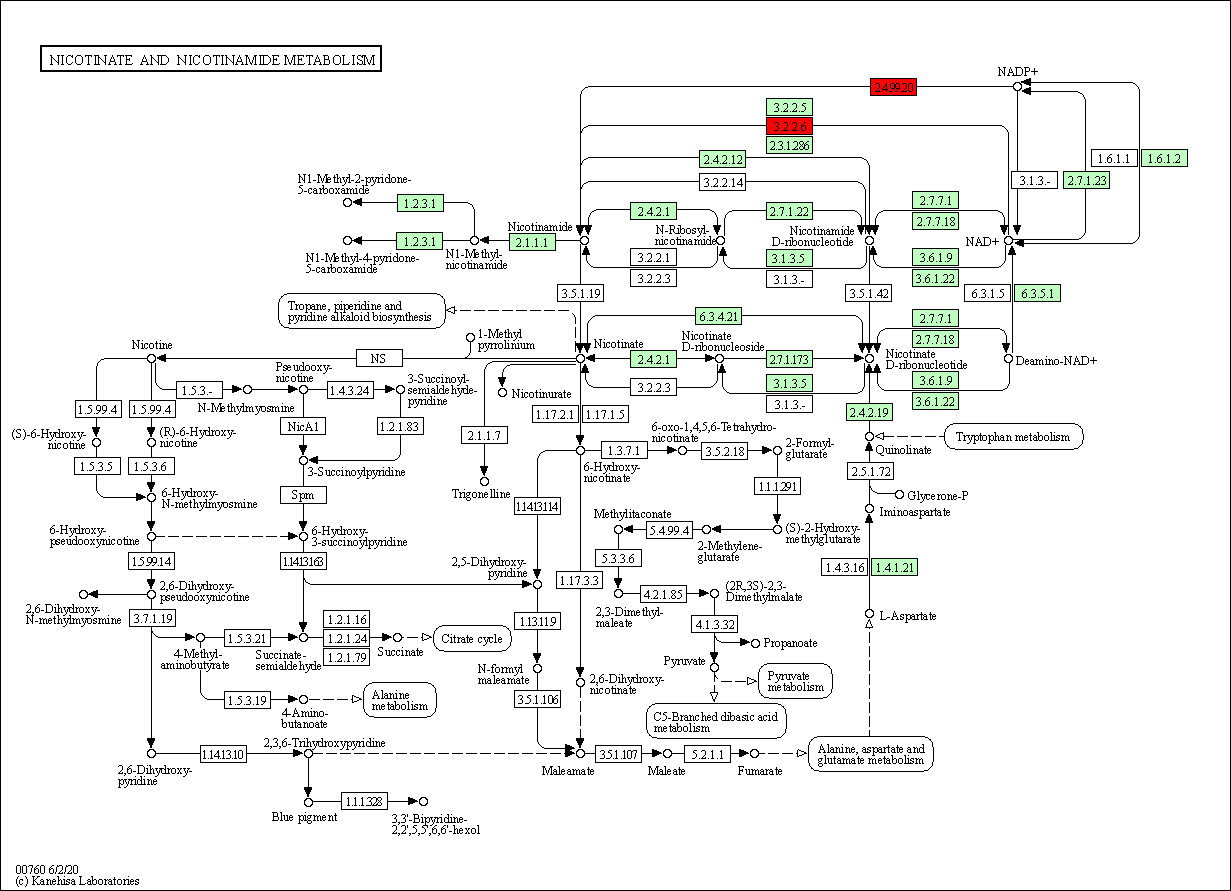

Supplement: Supplementary file 1 [file molecules-28-01606-s001.zip › raw data/KEGG/IL-1b_vs_N/DEG_pathway/mmu00760.png]

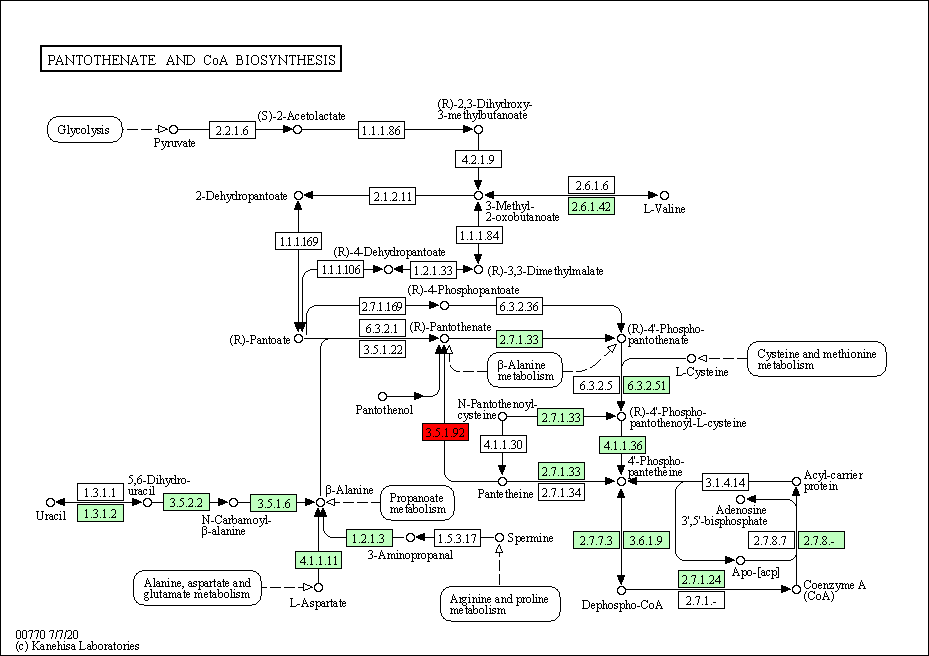

Supplement: Supplementary file 1 [file molecules-28-01606-s001.zip › raw data/KEGG/IL-1b_vs_N/DEG_pathway/mmu00770.png]

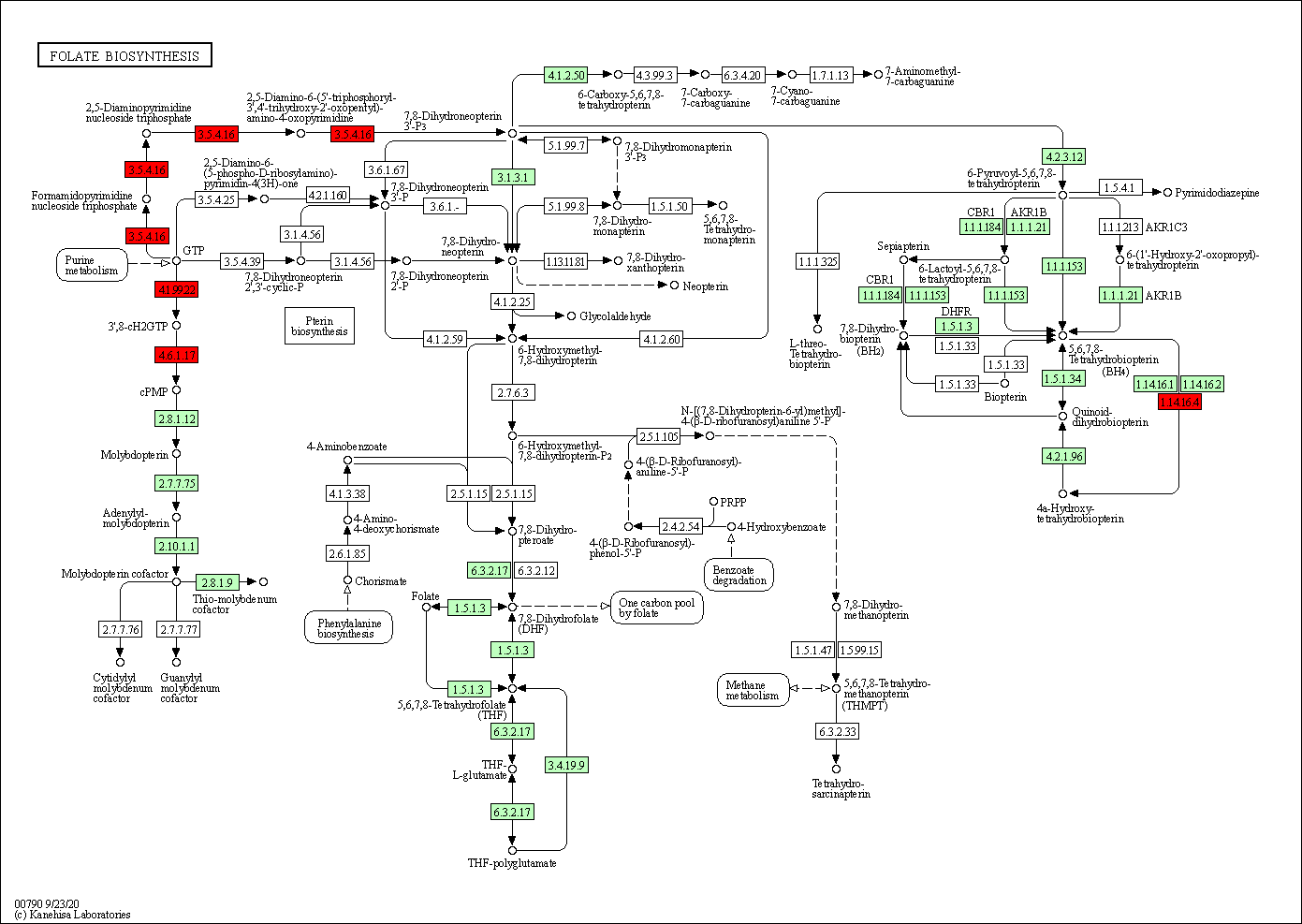

Supplement: Supplementary file 1 [file molecules-28-01606-s001.zip › raw data/KEGG/IL-1b_vs_N/DEG_pathway/mmu00790.png]

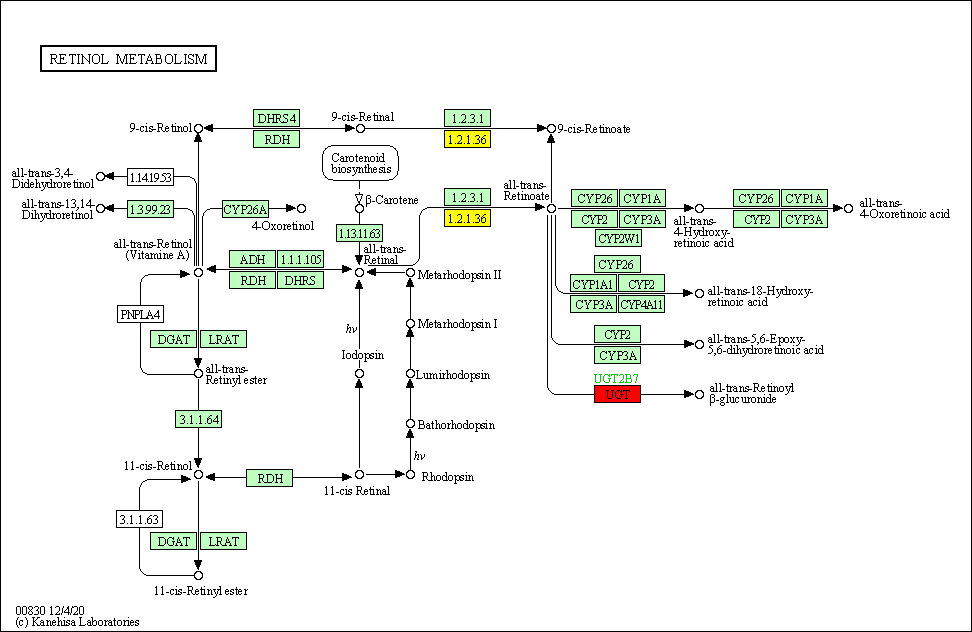

Supplement: Supplementary file 1 [file molecules-28-01606-s001.zip › raw data/KEGG/IL-1b_vs_N/DEG_pathway/mmu00830.png]

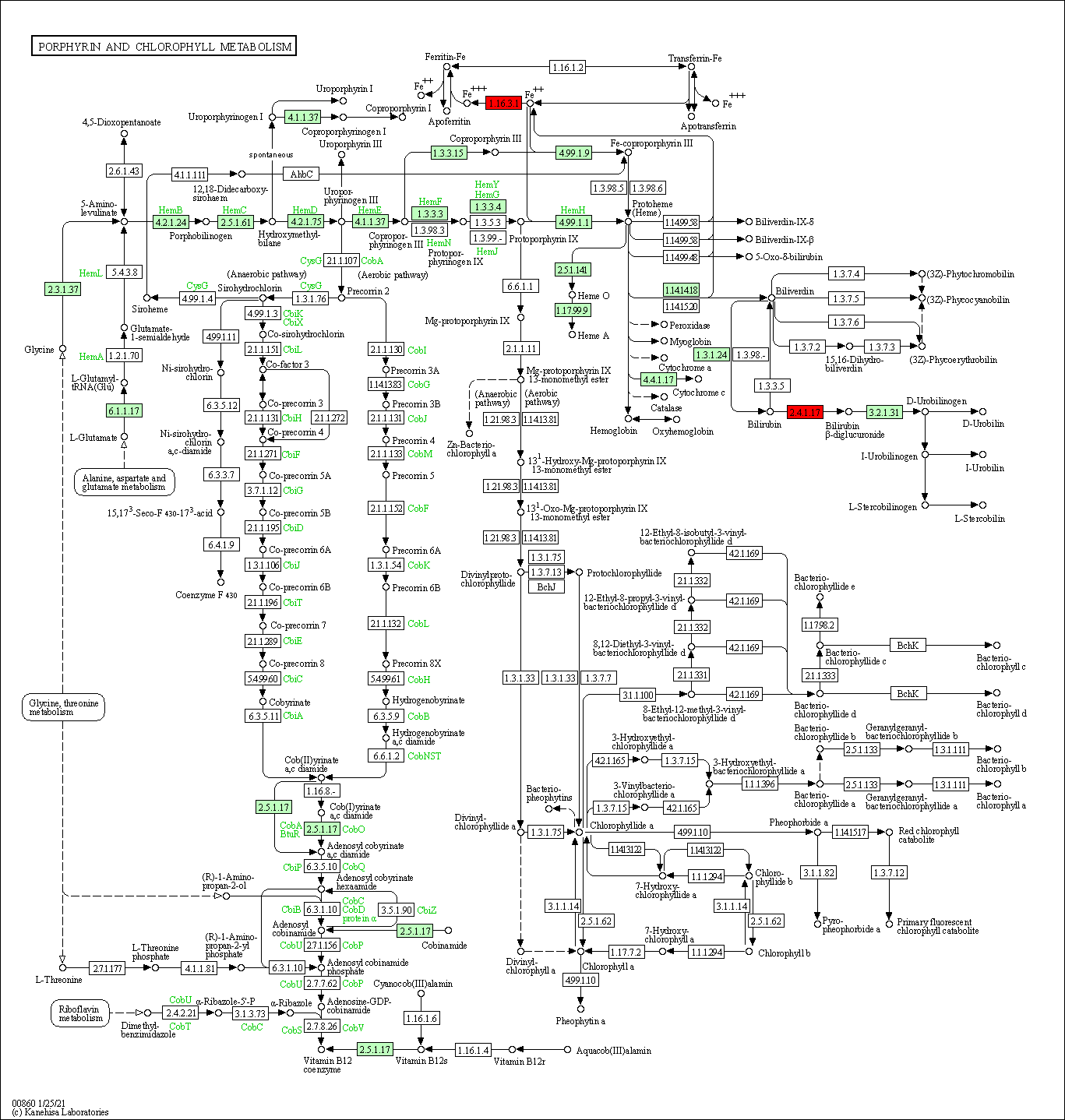

Supplement: Supplementary file 1 [file molecules-28-01606-s001.zip › raw data/KEGG/IL-1b_vs_N/DEG_pathway/mmu00860.png]

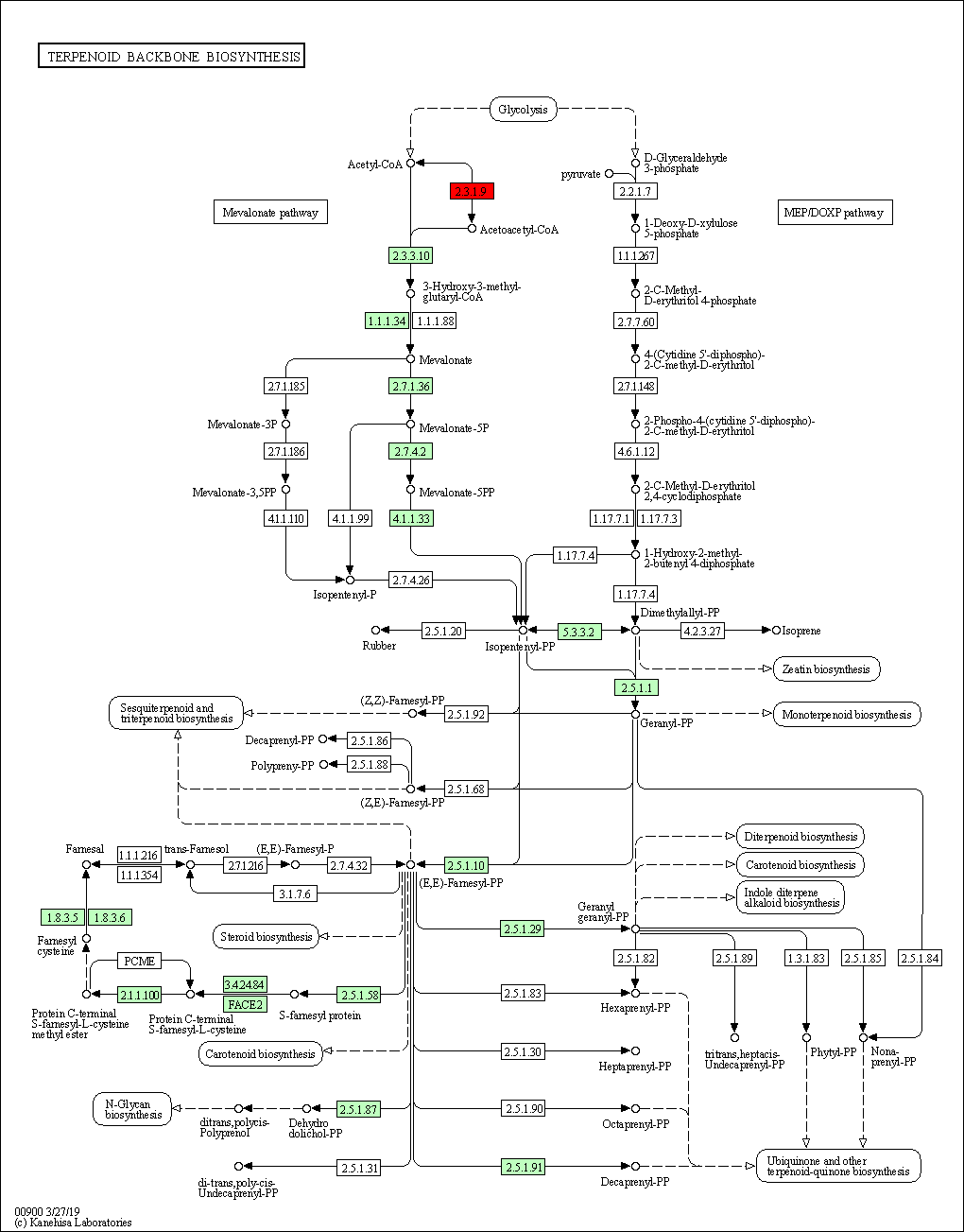

Supplement: Supplementary file 1 [file molecules-28-01606-s001.zip › raw data/KEGG/IL-1b_vs_N/DEG_pathway/mmu00900.png]

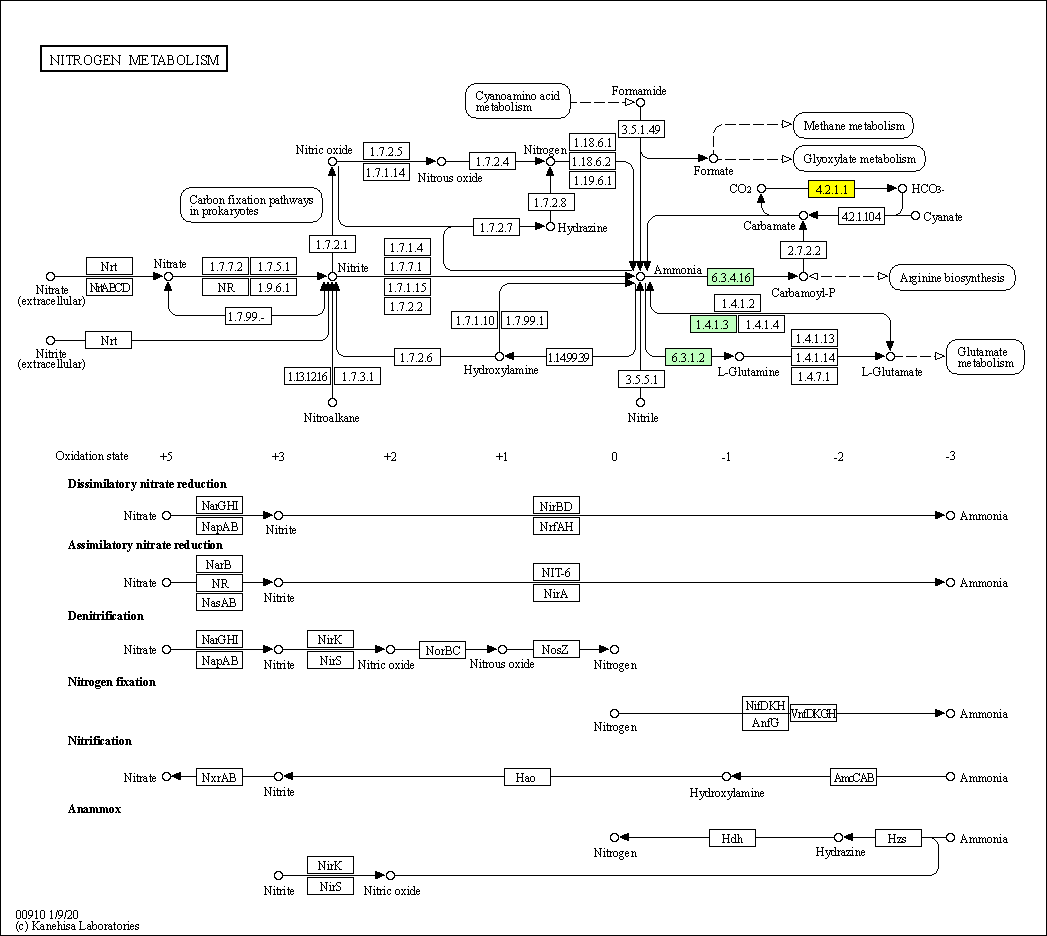

Supplement: Supplementary file 1 [file molecules-28-01606-s001.zip › raw data/KEGG/IL-1b_vs_N/DEG_pathway/mmu00910.png]

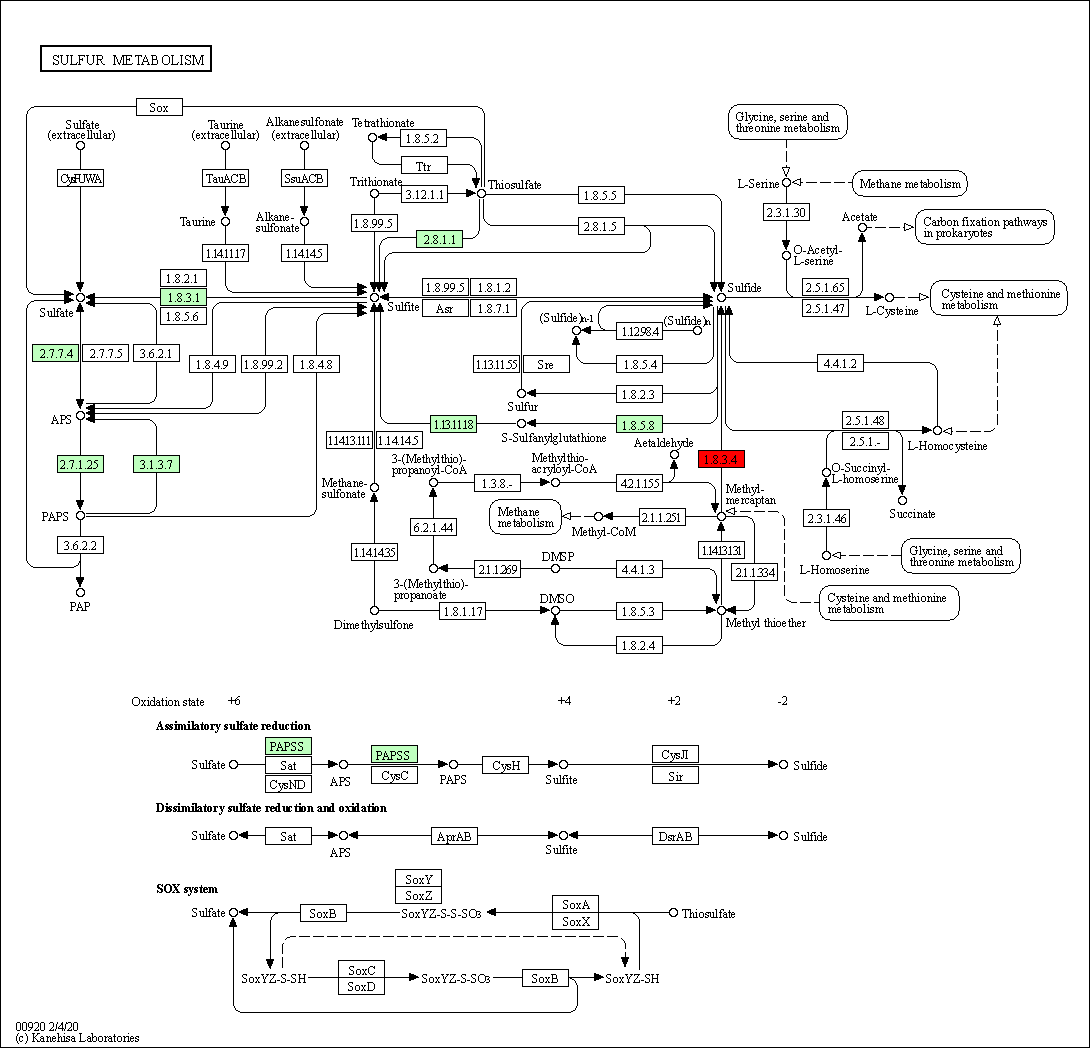

Supplement: Supplementary file 1 [file molecules-28-01606-s001.zip › raw data/KEGG/IL-1b_vs_N/DEG_pathway/mmu00920.png]

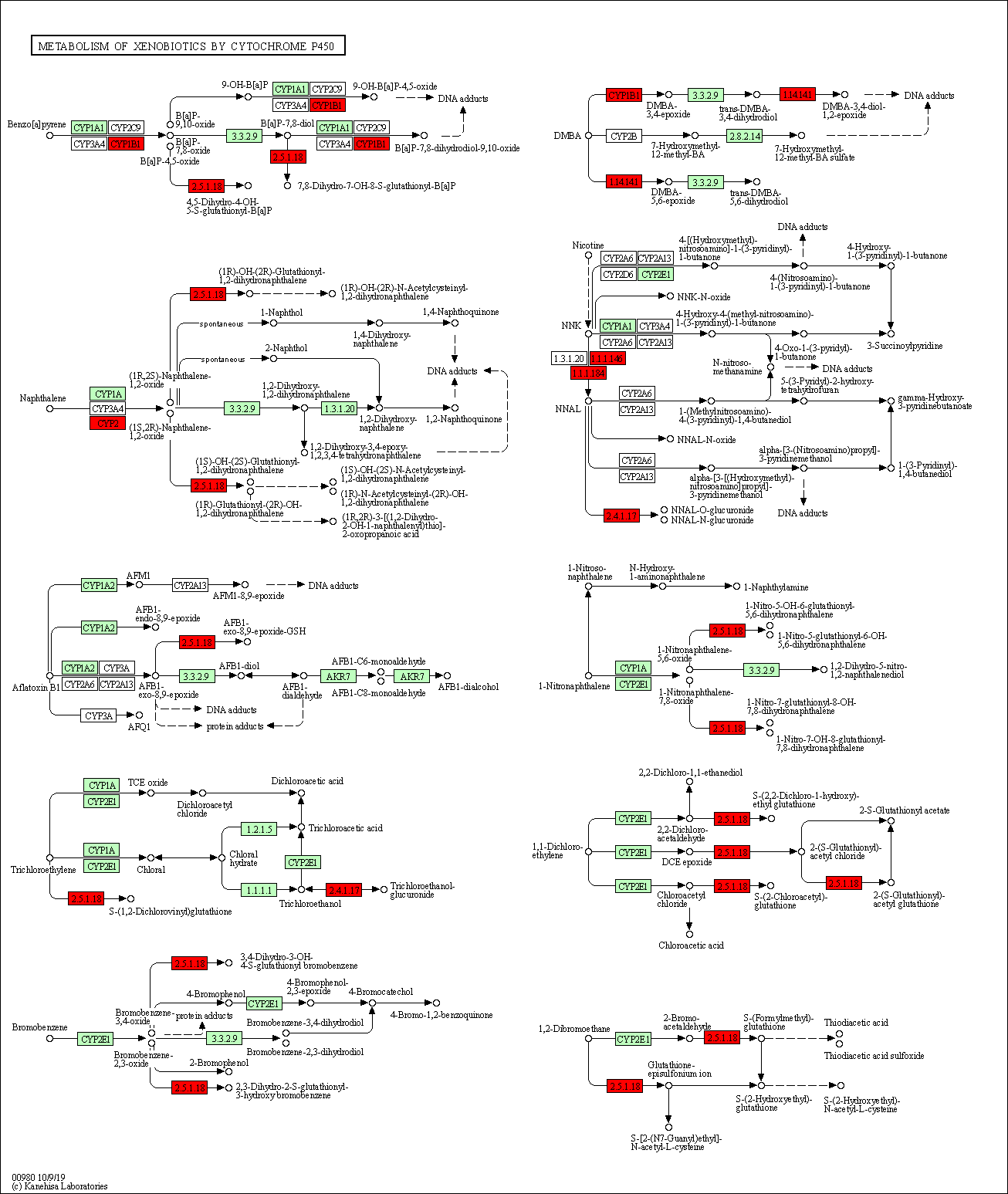

Supplement: Supplementary file 1 [file molecules-28-01606-s001.zip › raw data/KEGG/IL-1b_vs_N/DEG_pathway/mmu00980.png]

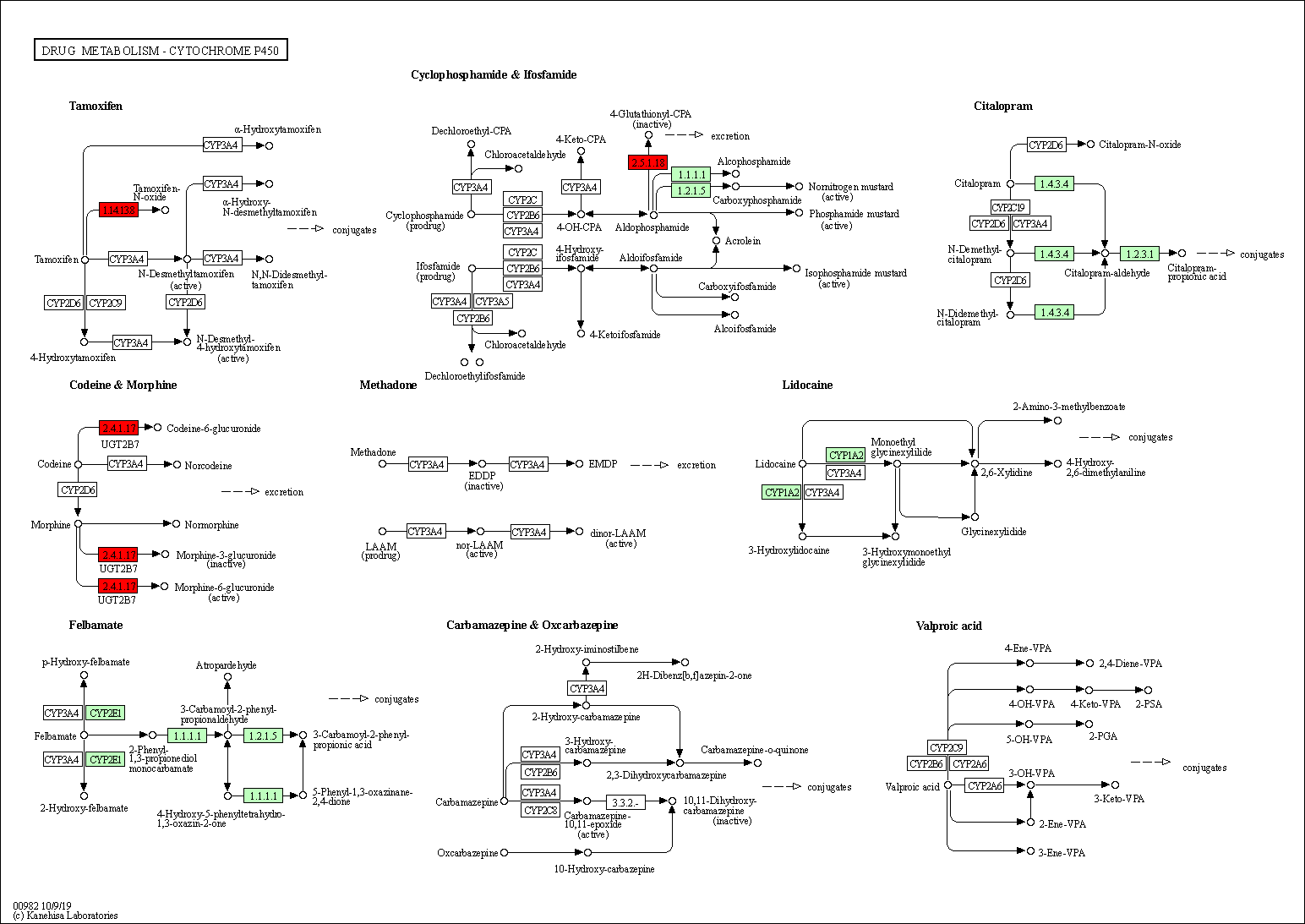

Supplement: Supplementary file 1 [file molecules-28-01606-s001.zip › raw data/KEGG/IL-1b_vs_N/DEG_pathway/mmu00982.png]

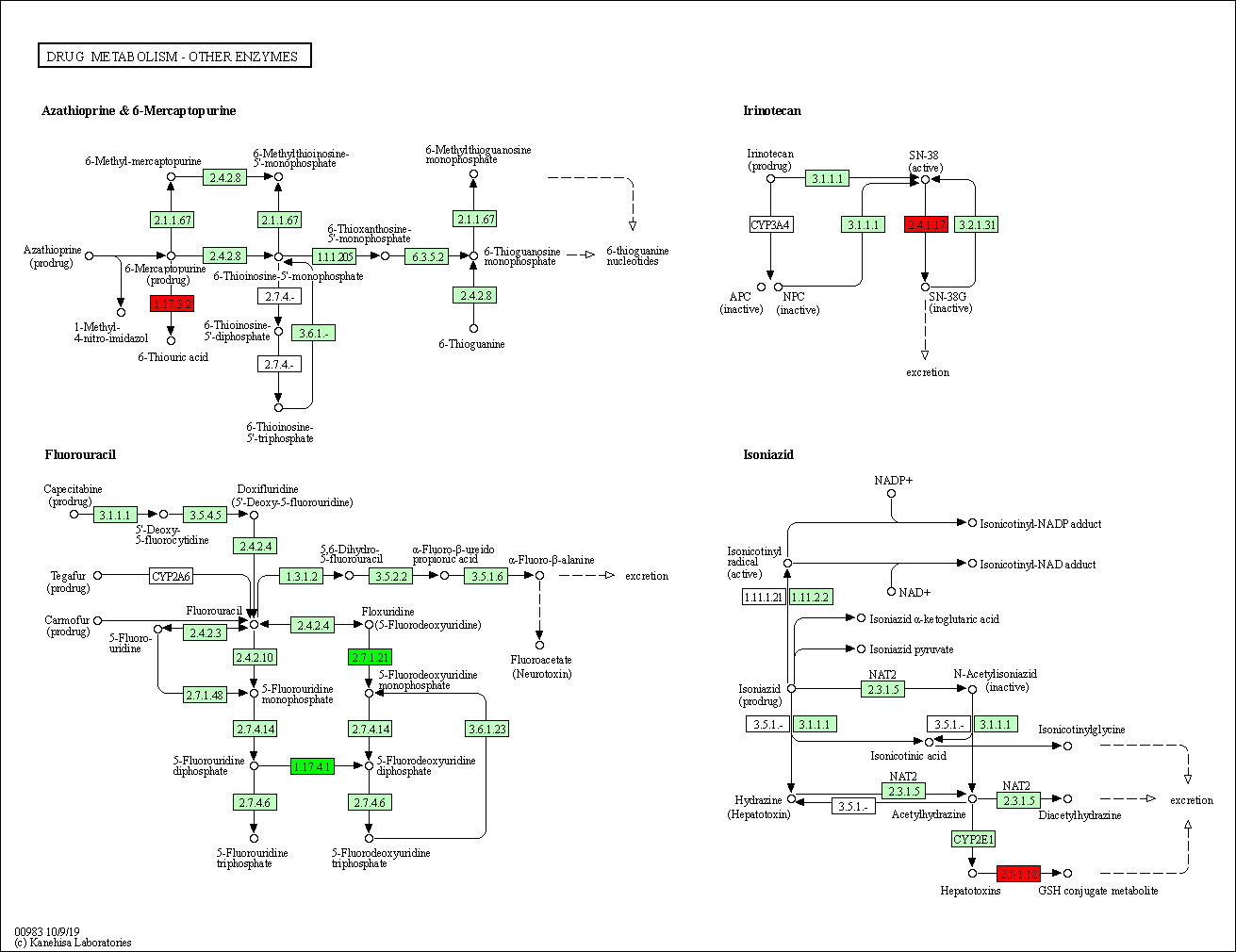

Supplement: Supplementary file 1 [file molecules-28-01606-s001.zip › raw data/KEGG/IL-1b_vs_N/DEG_pathway/mmu00983.png]

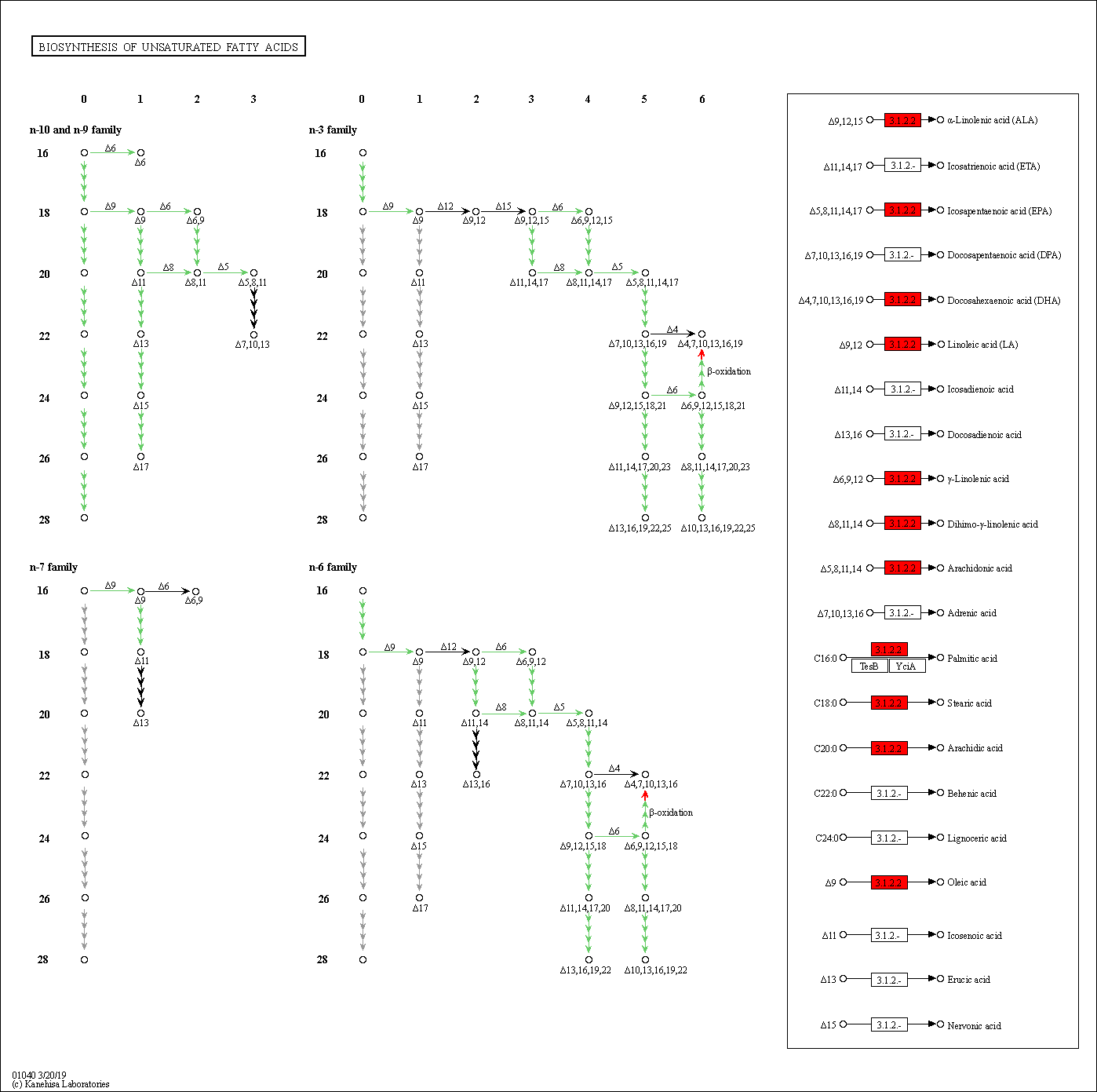

Supplement: Supplementary file 1 [file molecules-28-01606-s001.zip › raw data/KEGG/IL-1b_vs_N/DEG_pathway/mmu01040.png]

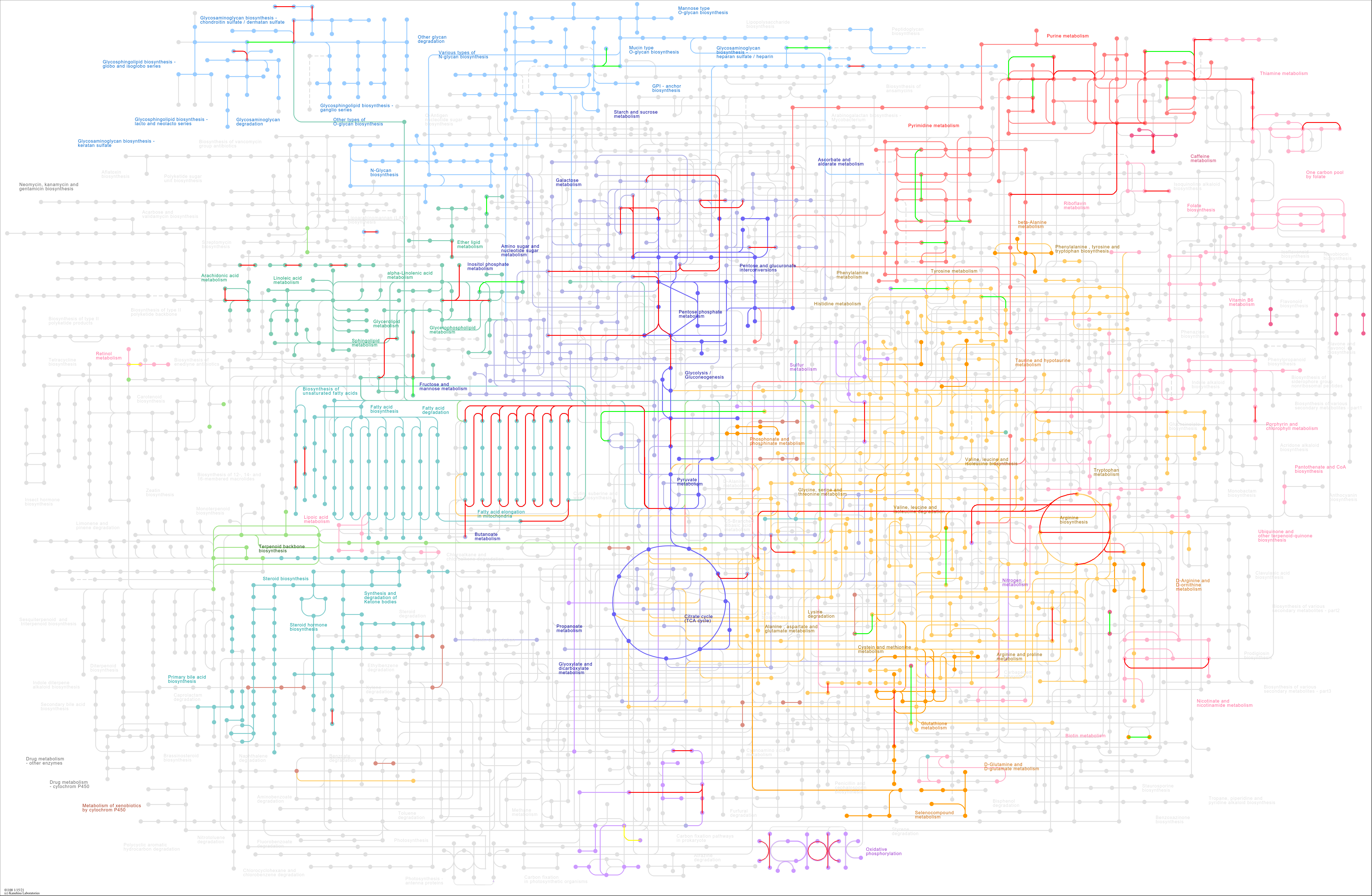

Supplement: Supplementary file 1 [file molecules-28-01606-s001.zip › raw data/KEGG/IL-1b_vs_N/DEG_pathway/mmu01100.png]

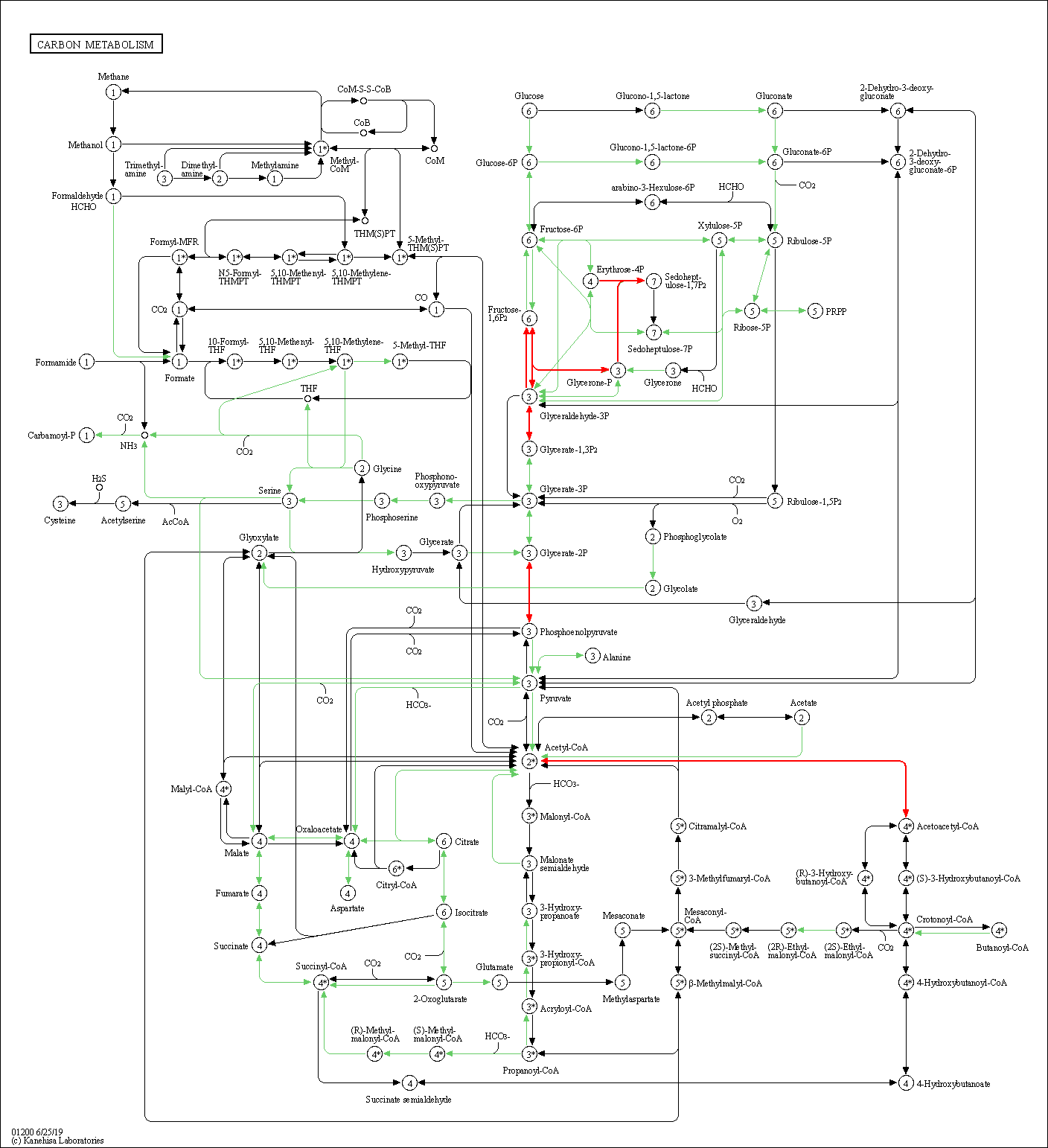

Supplement: Supplementary file 1 [file molecules-28-01606-s001.zip › raw data/KEGG/IL-1b_vs_N/DEG_pathway/mmu01200.png]

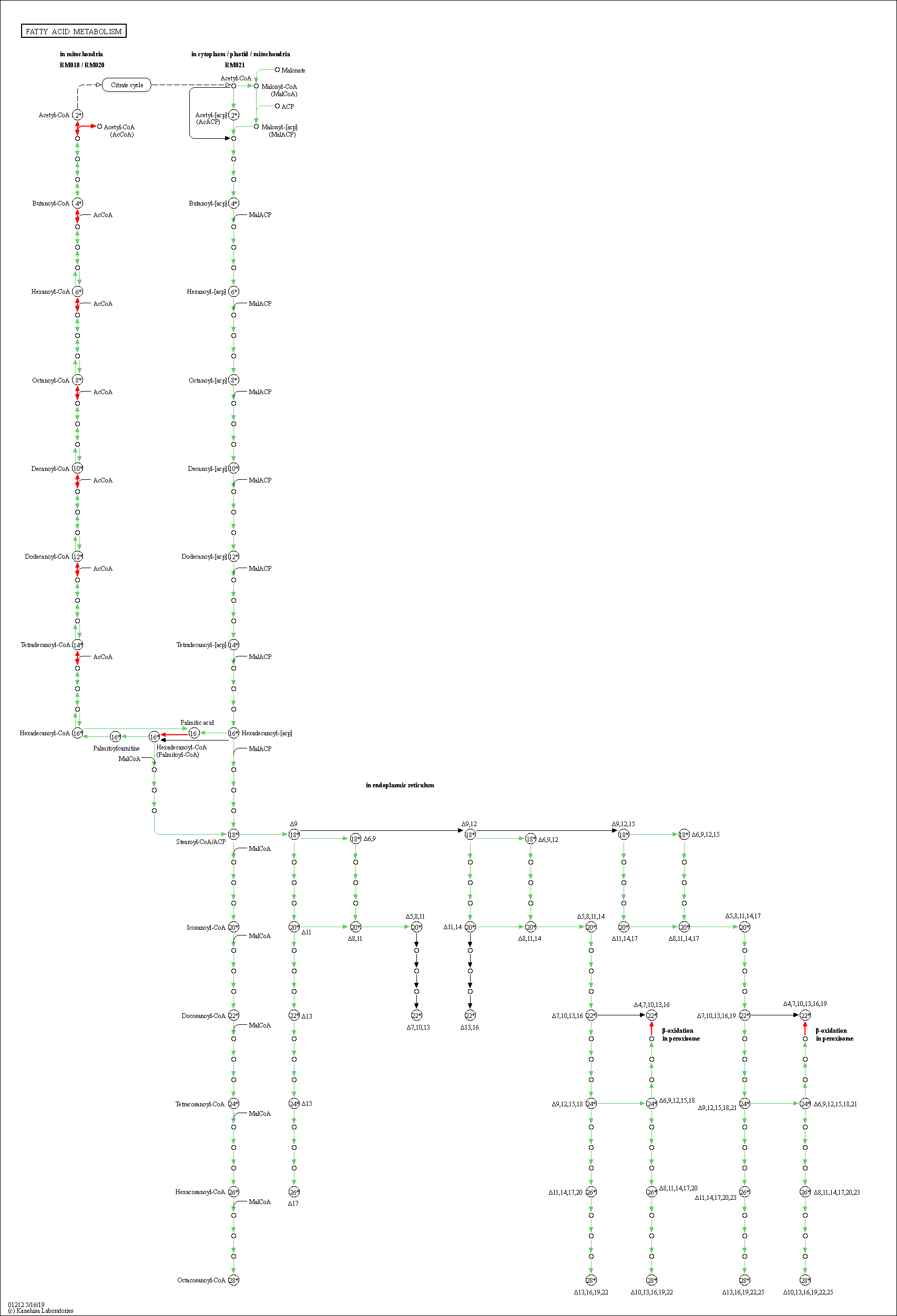

Supplement: Supplementary file 1 [file molecules-28-01606-s001.zip › raw data/KEGG/IL-1b_vs_N/DEG_pathway/mmu01212.png]

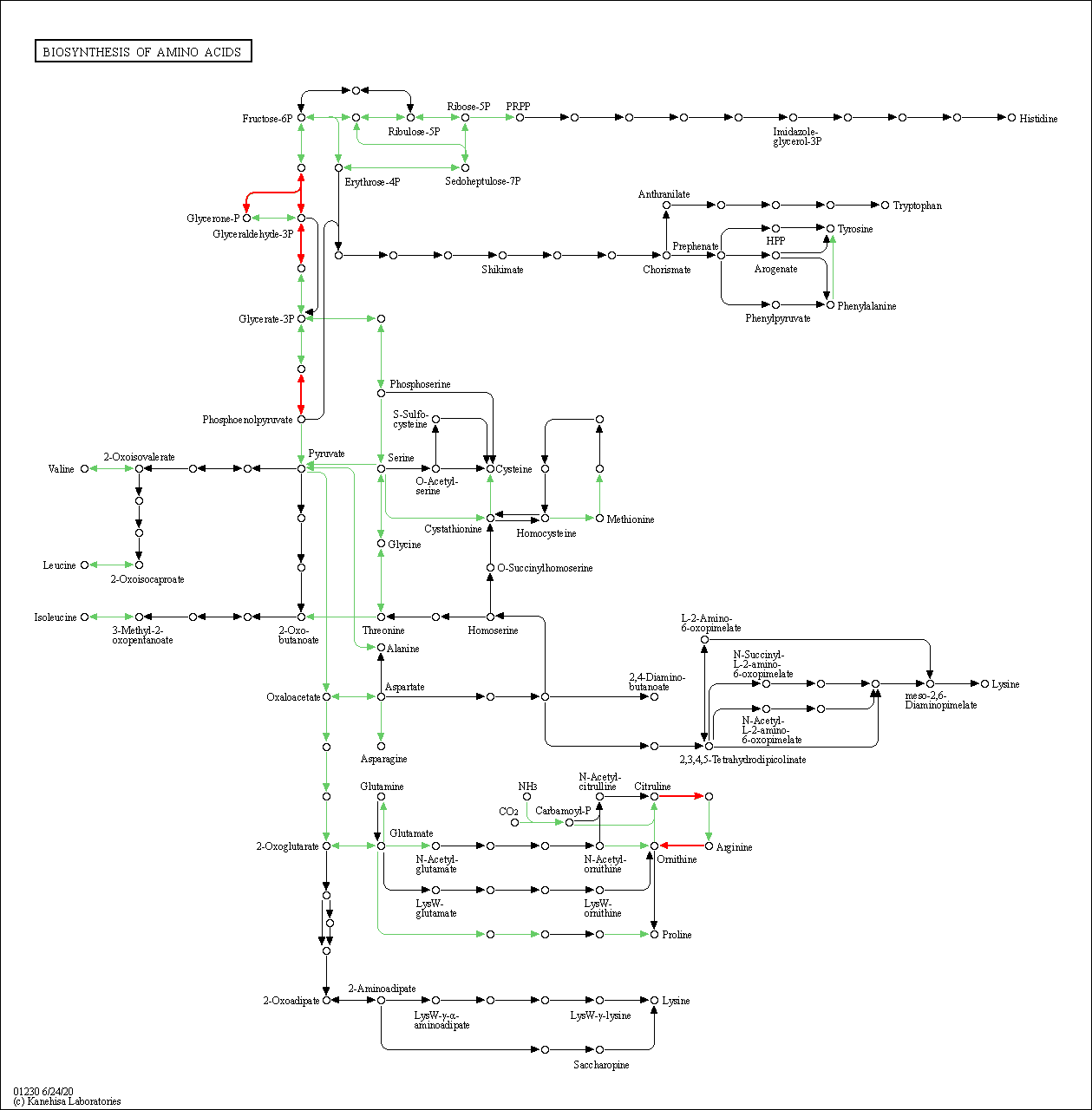

Supplement: Supplementary file 1 [file molecules-28-01606-s001.zip › raw data/KEGG/IL-1b_vs_N/DEG_pathway/mmu01230.png]

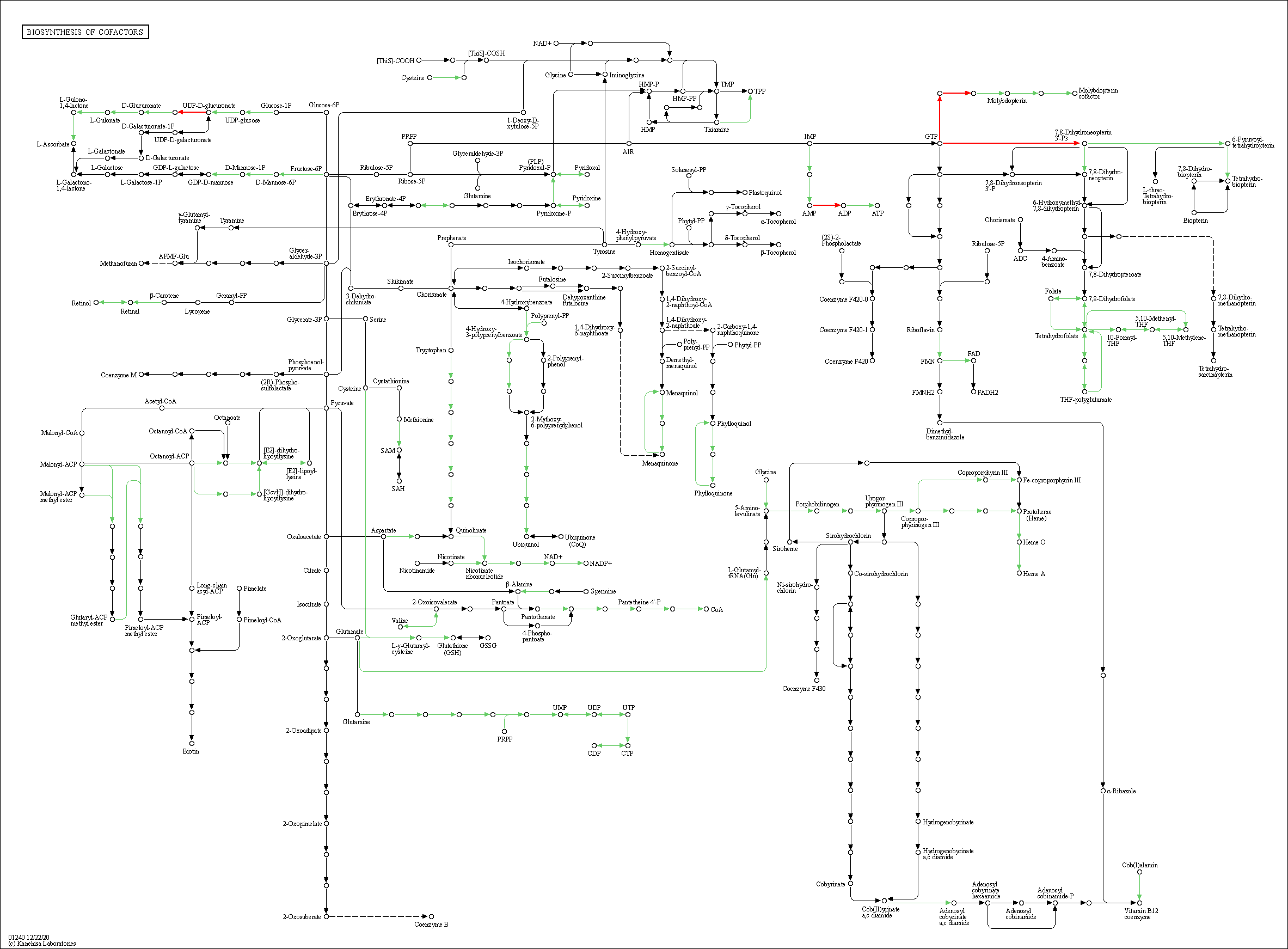

Supplement: Supplementary file 1 [file molecules-28-01606-s001.zip › raw data/KEGG/IL-1b_vs_N/DEG_pathway/mmu01240.png]

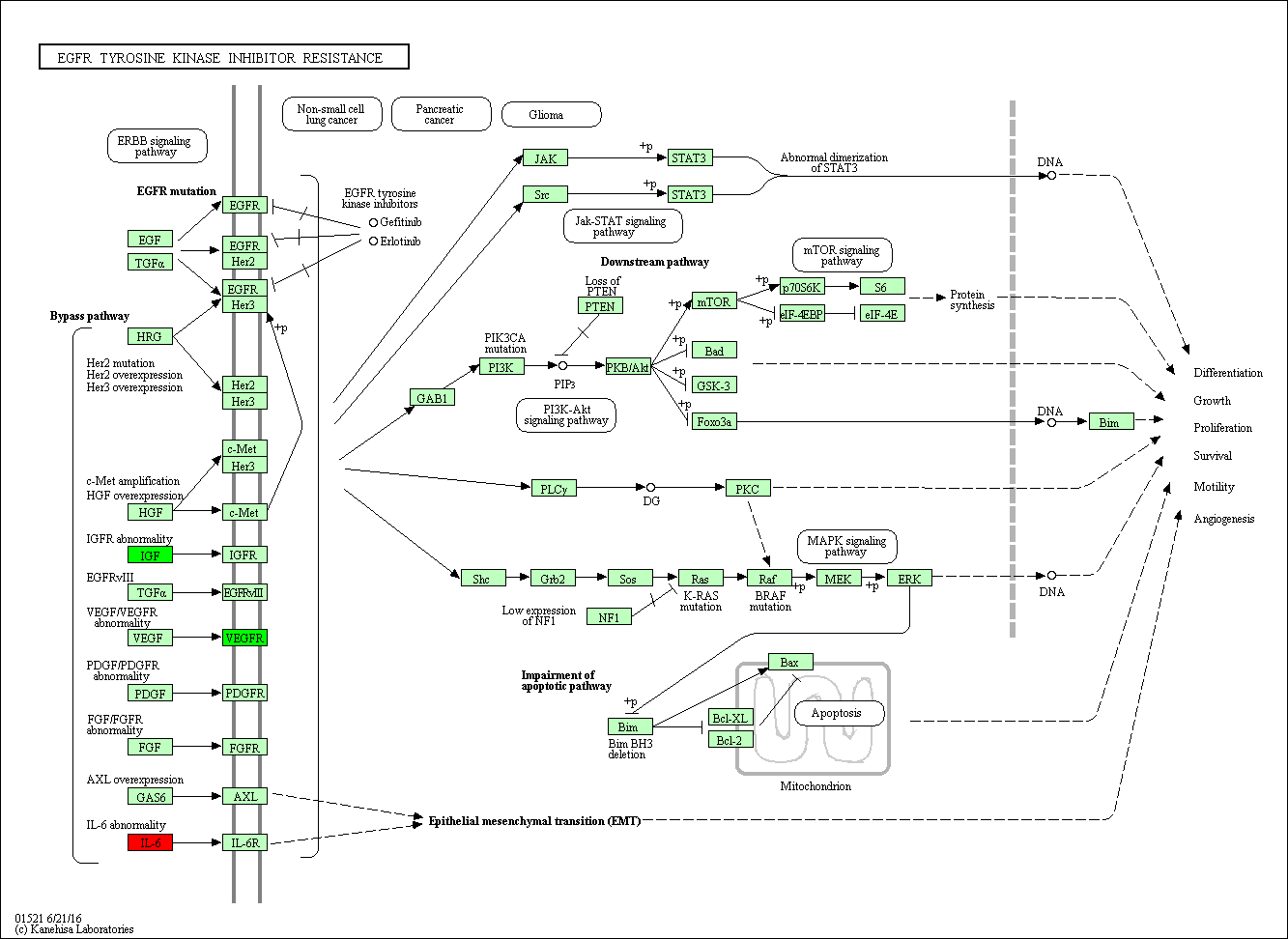

Supplement: Supplementary file 1 [file molecules-28-01606-s001.zip › raw data/KEGG/IL-1b_vs_N/DEG_pathway/mmu01521.png]

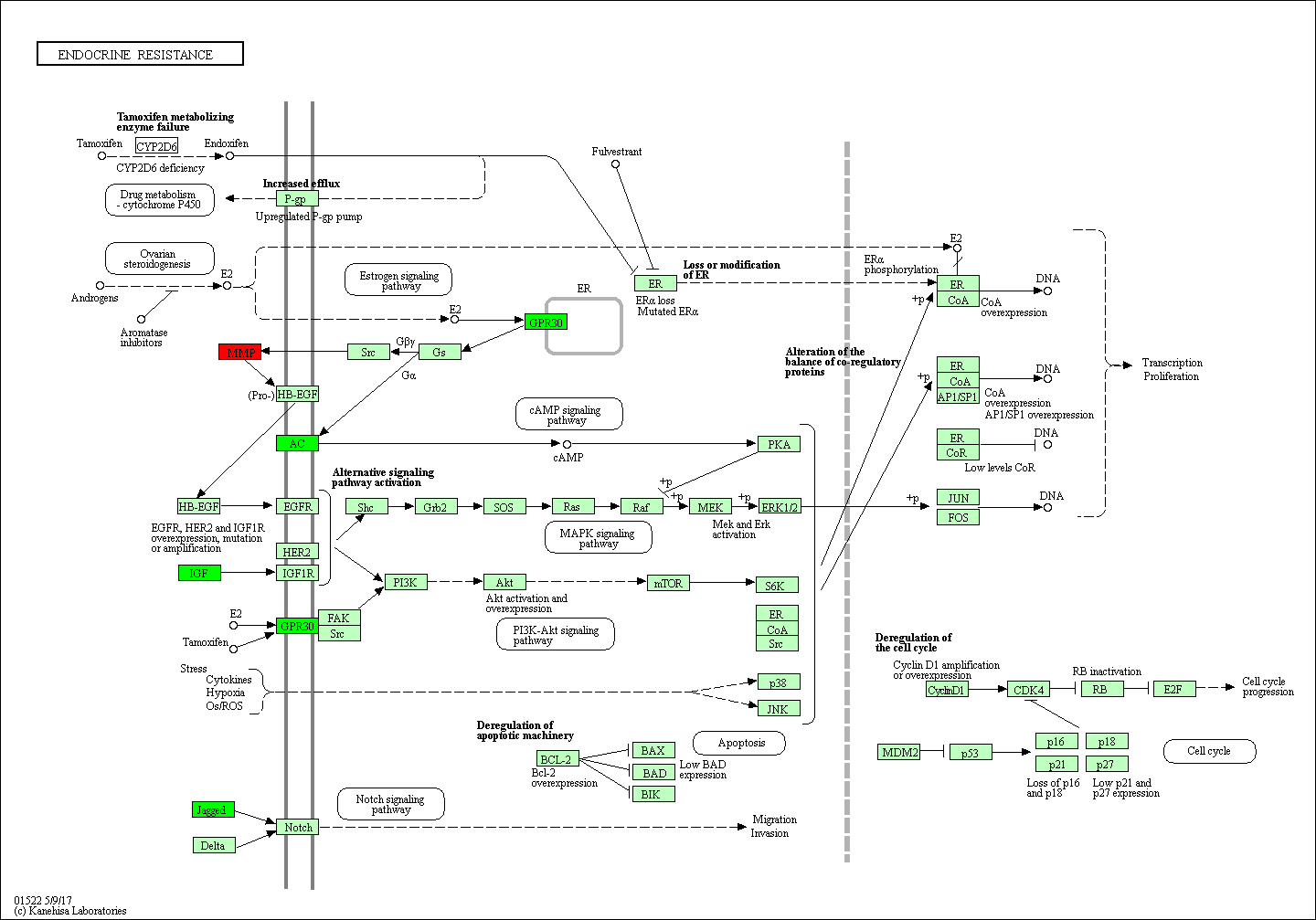

Supplement: Supplementary file 1 [file molecules-28-01606-s001.zip › raw data/KEGG/IL-1b_vs_N/DEG_pathway/mmu01522.png]

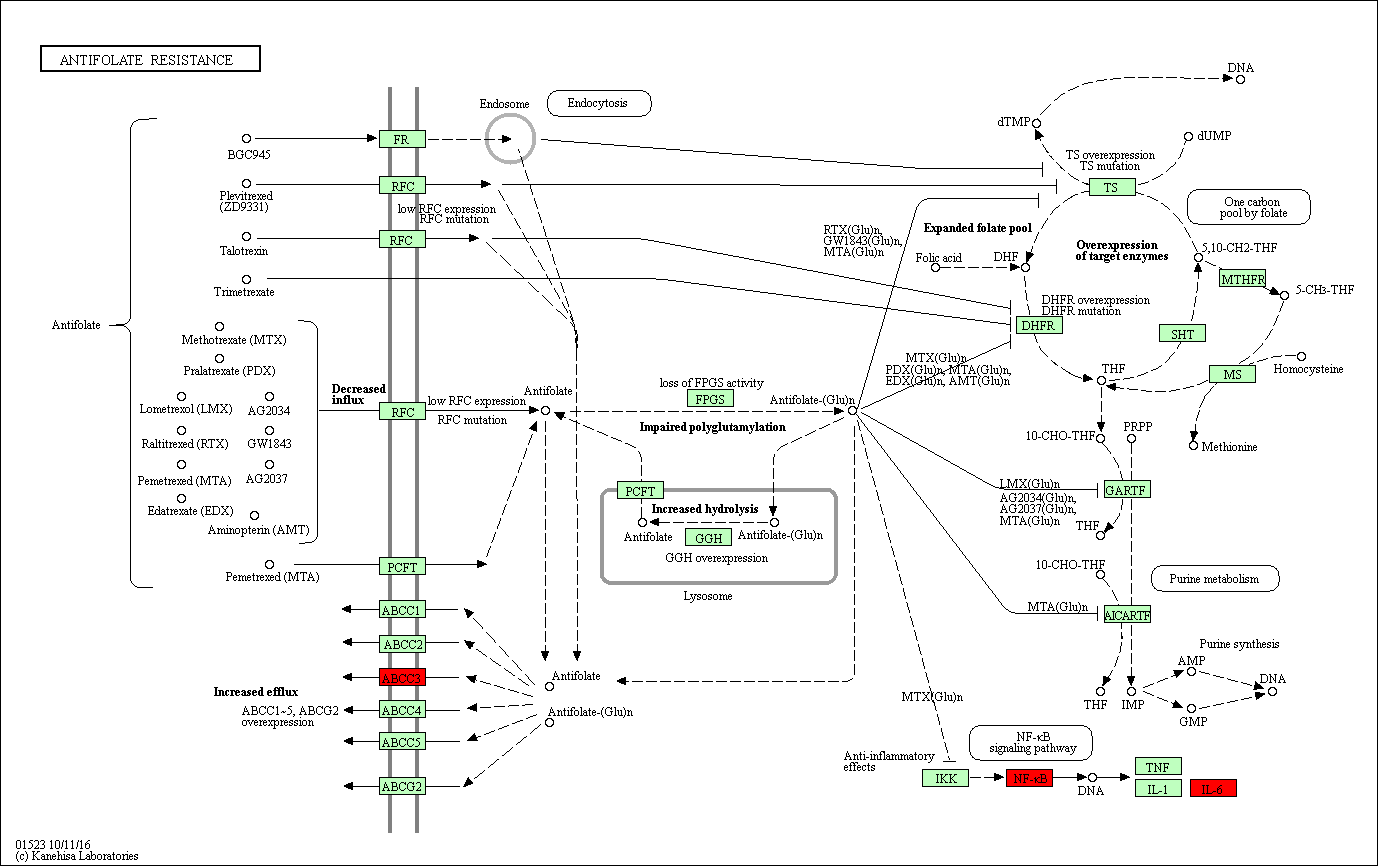

Supplement: Supplementary file 1 [file molecules-28-01606-s001.zip › raw data/KEGG/IL-1b_vs_N/DEG_pathway/mmu01523.png]

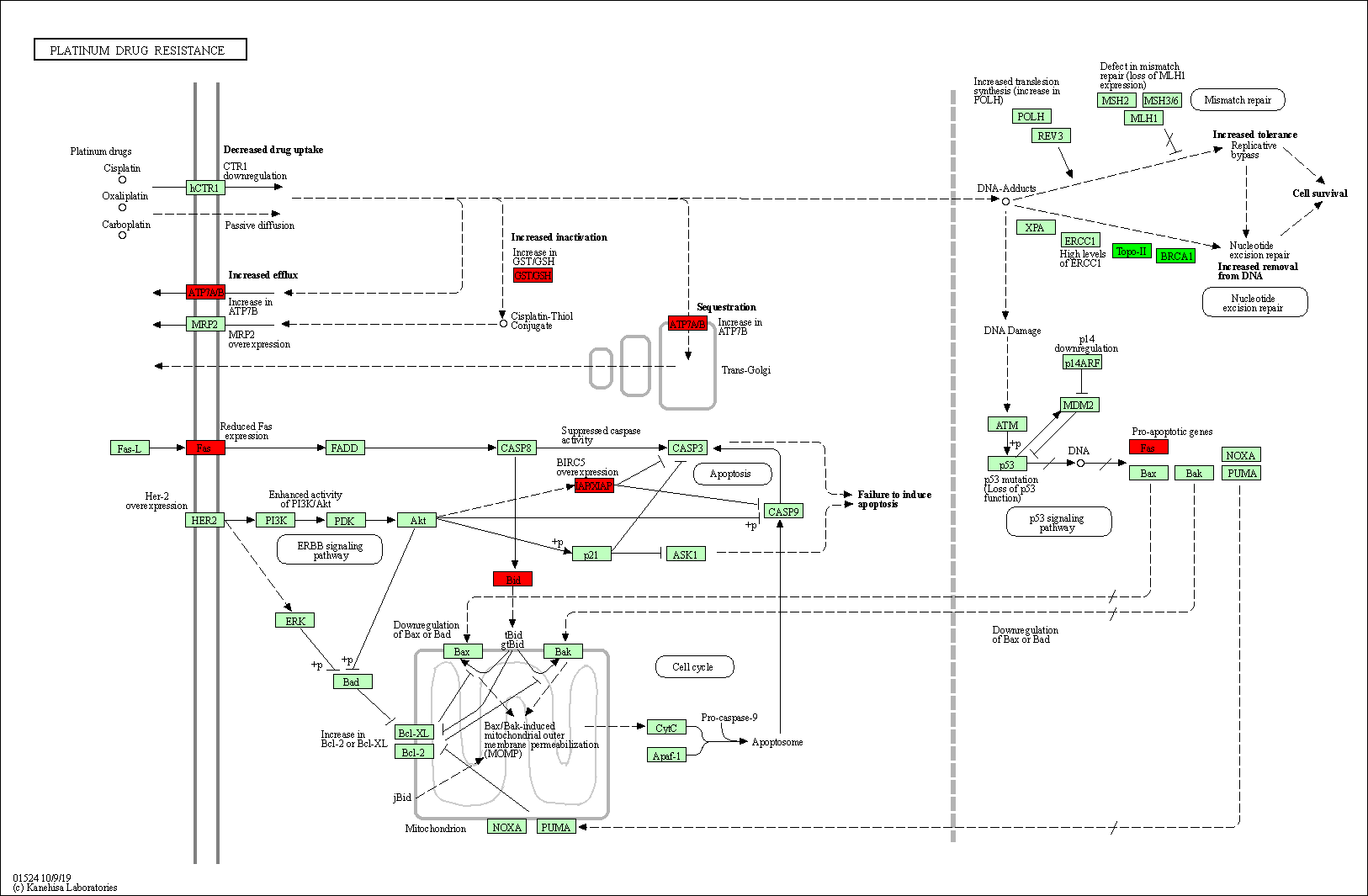

Supplement: Supplementary file 1 [file molecules-28-01606-s001.zip › raw data/KEGG/IL-1b_vs_N/DEG_pathway/mmu01524.png]

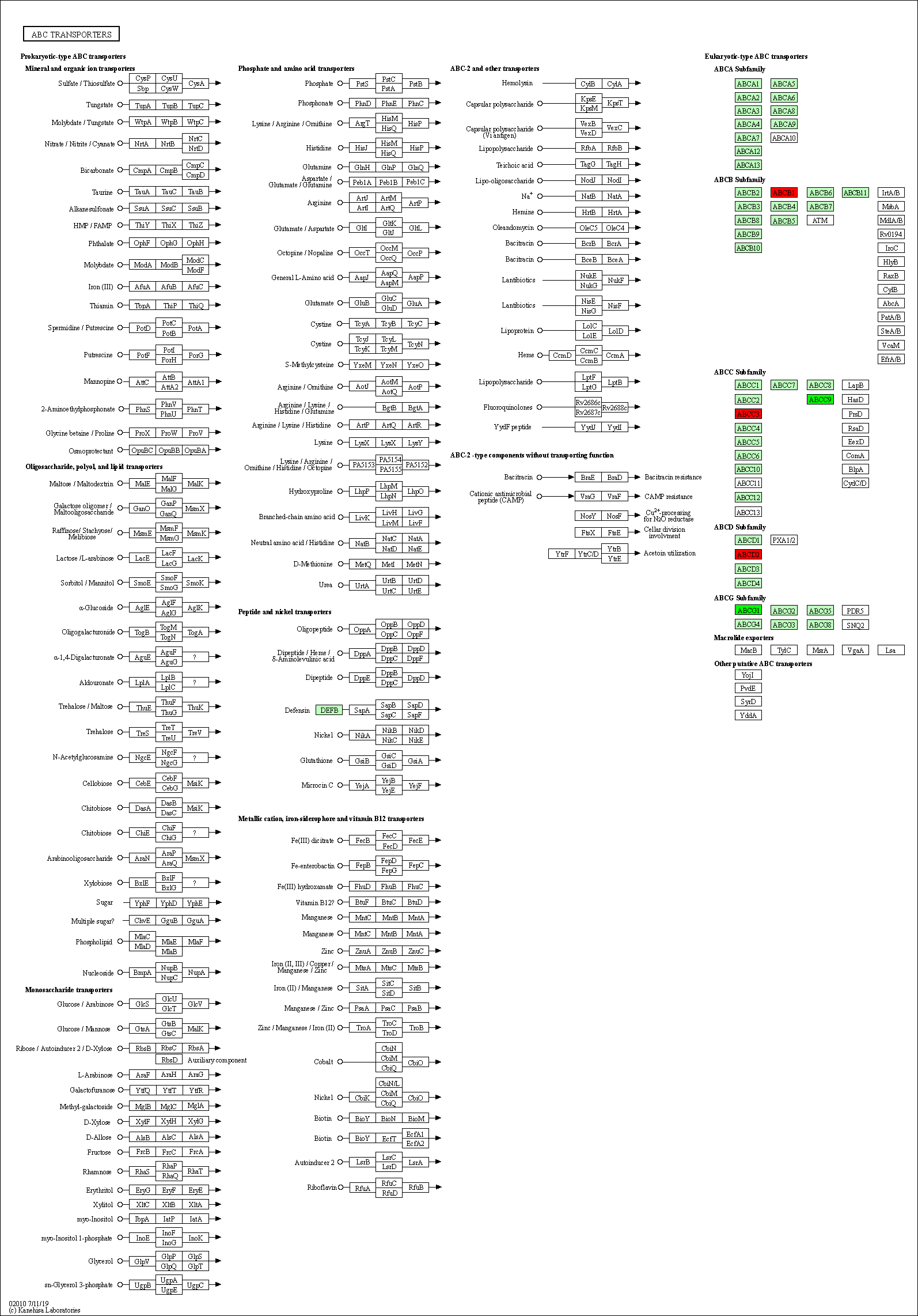

Supplement: Supplementary file 1 [file molecules-28-01606-s001.zip › raw data/KEGG/IL-1b_vs_N/DEG_pathway/mmu02010.png]

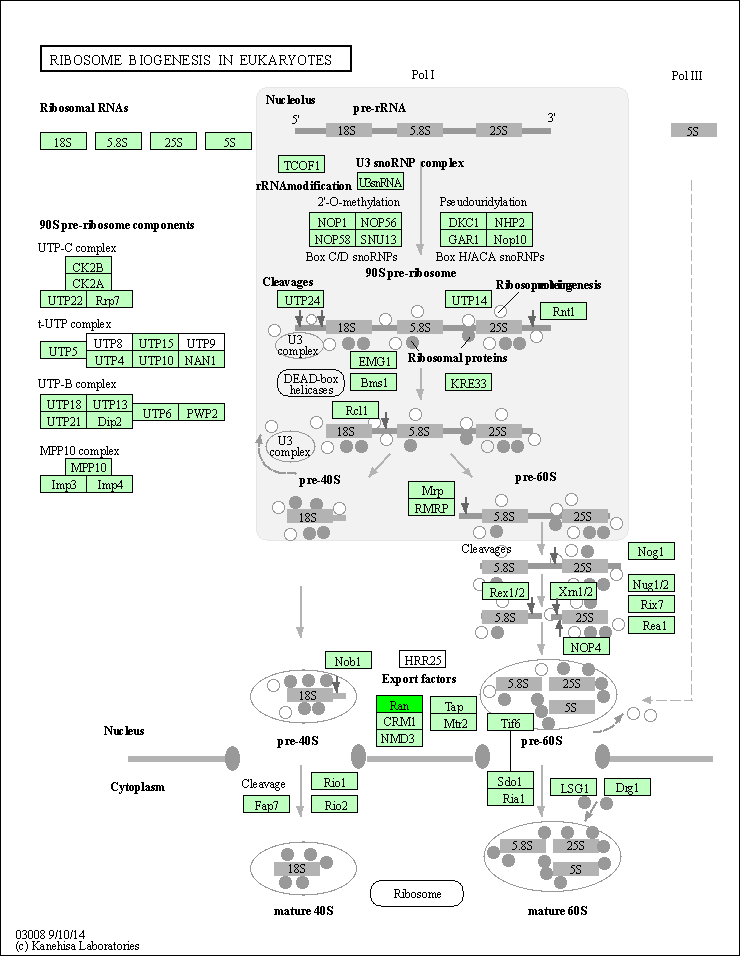

Supplement: Supplementary file 1 [file molecules-28-01606-s001.zip › raw data/KEGG/IL-1b_vs_N/DEG_pathway/mmu03008.png]

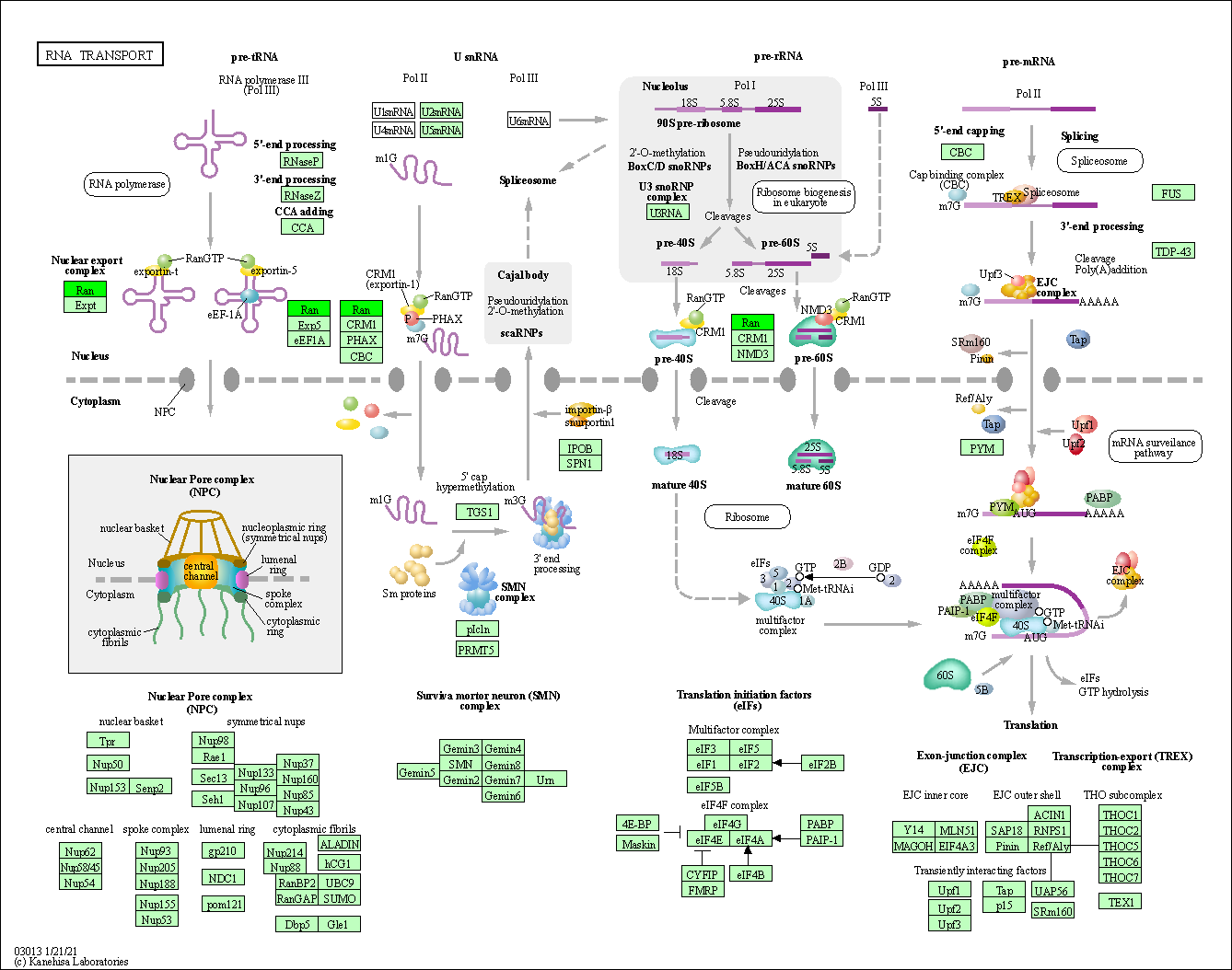

Supplement: Supplementary file 1 [file molecules-28-01606-s001.zip › raw data/KEGG/IL-1b_vs_N/DEG_pathway/mmu03013.png]

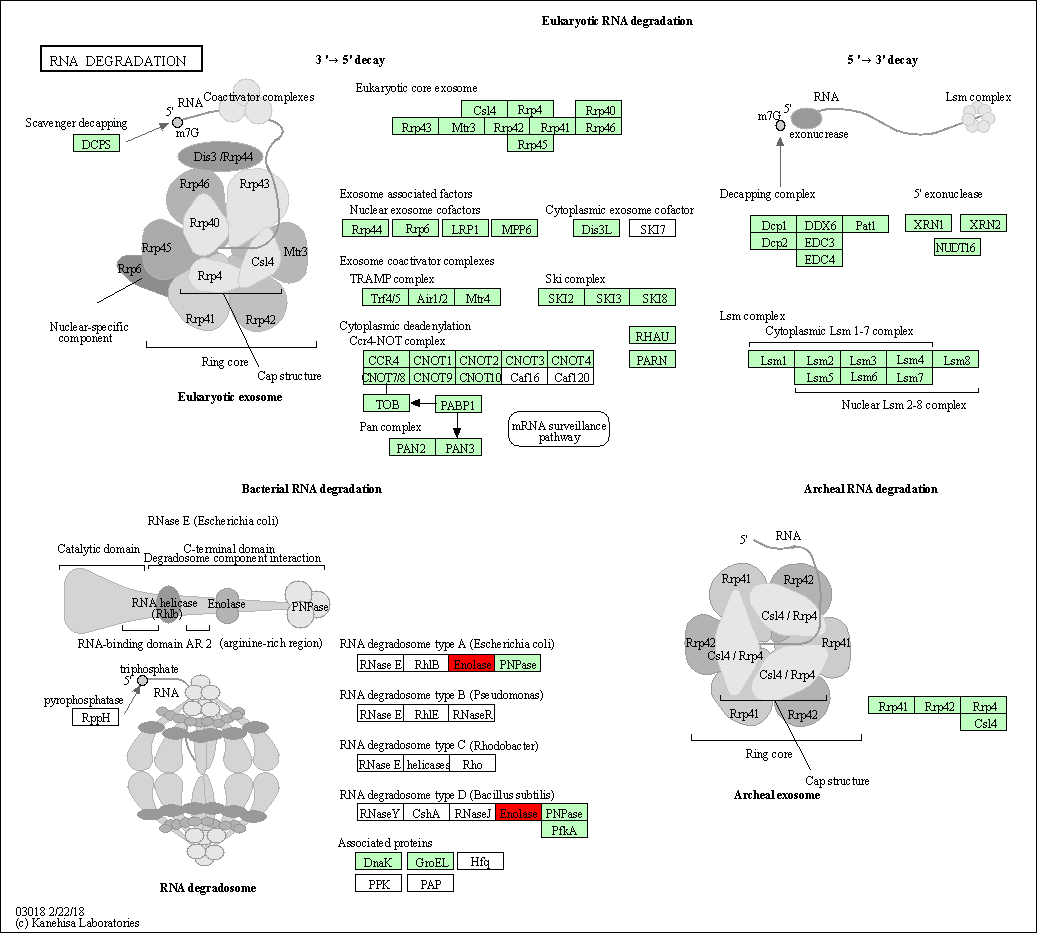

Supplement: Supplementary file 1 [file molecules-28-01606-s001.zip › raw data/KEGG/IL-1b_vs_N/DEG_pathway/mmu03018.png]

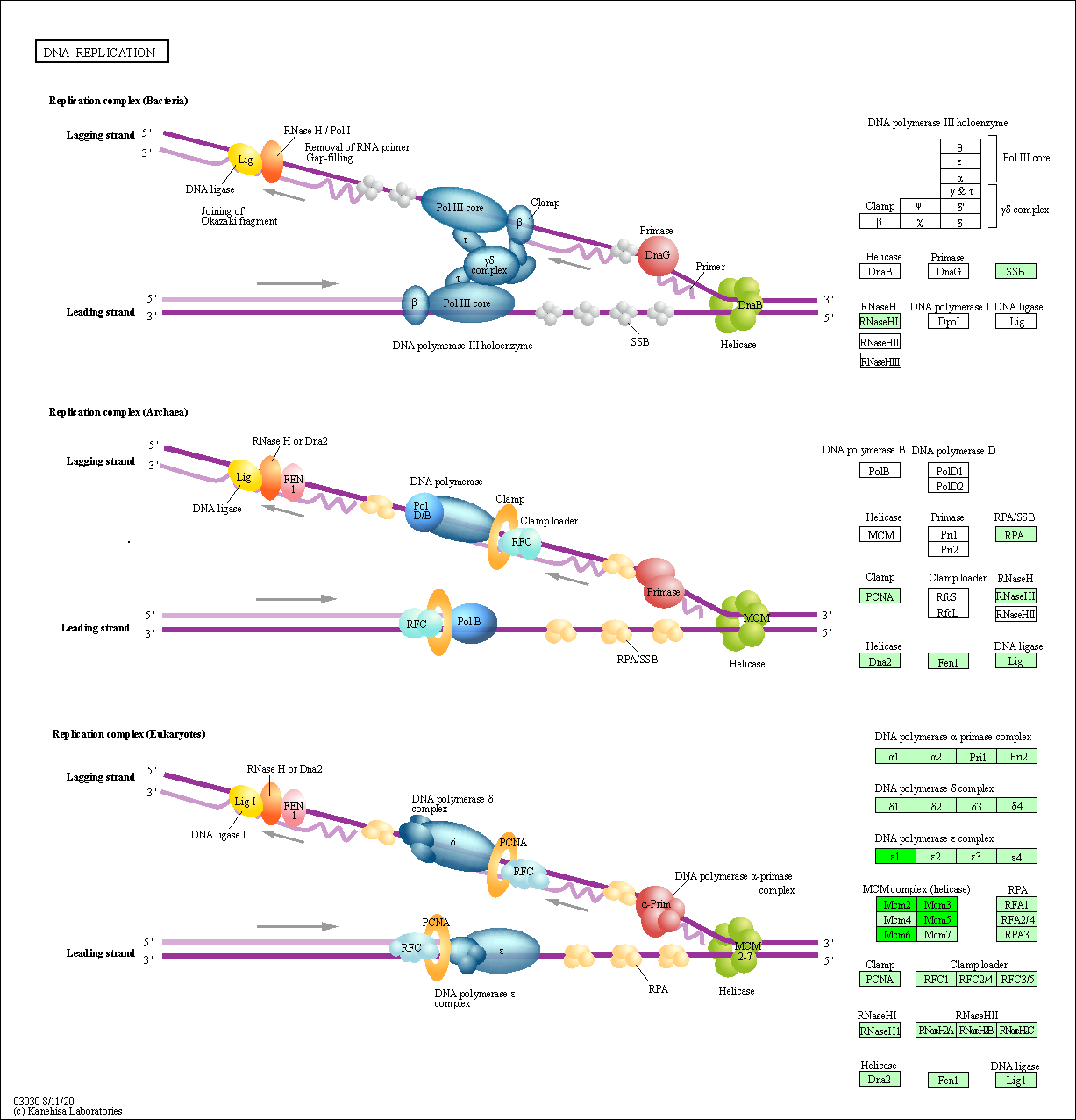

Supplement: Supplementary file 1 [file molecules-28-01606-s001.zip › raw data/KEGG/IL-1b_vs_N/DEG_pathway/mmu03030.png]

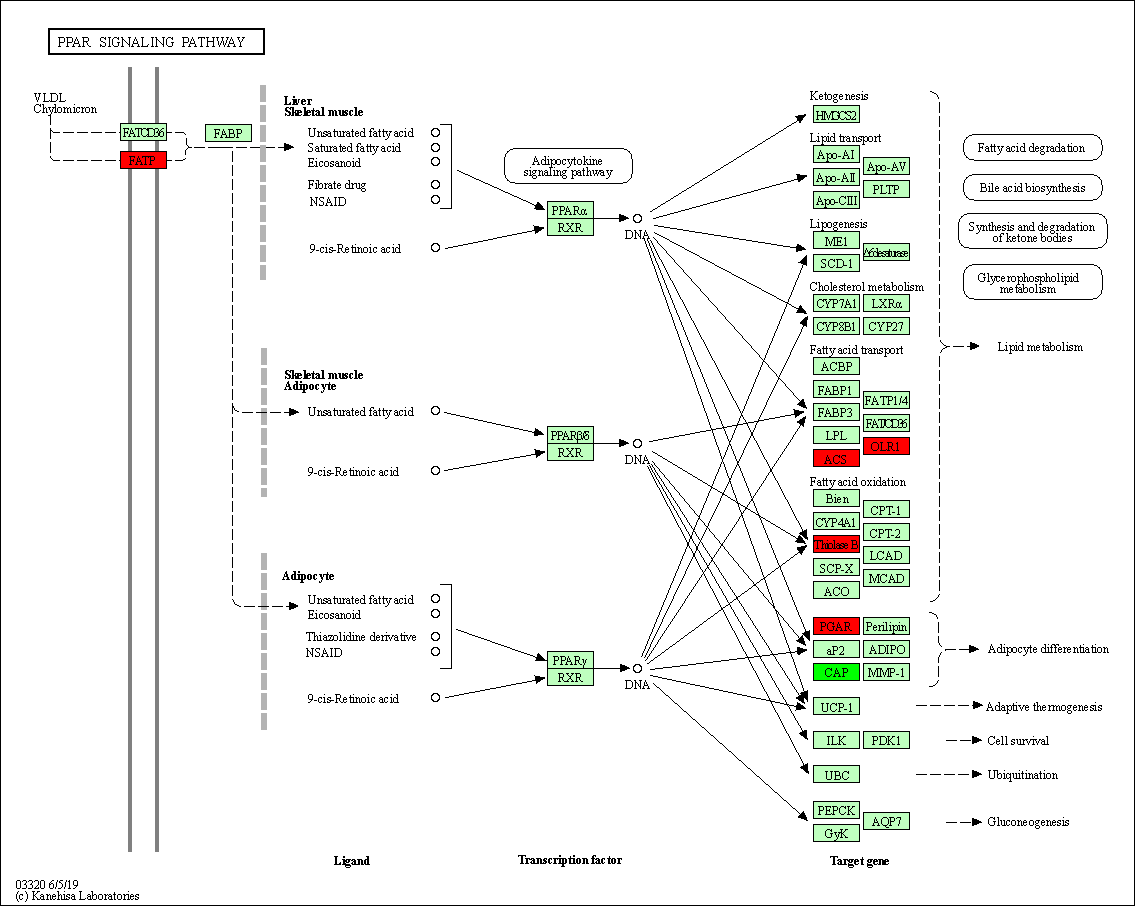

Supplement: Supplementary file 1 [file molecules-28-01606-s001.zip › raw data/KEGG/IL-1b_vs_N/DEG_pathway/mmu03320.png]

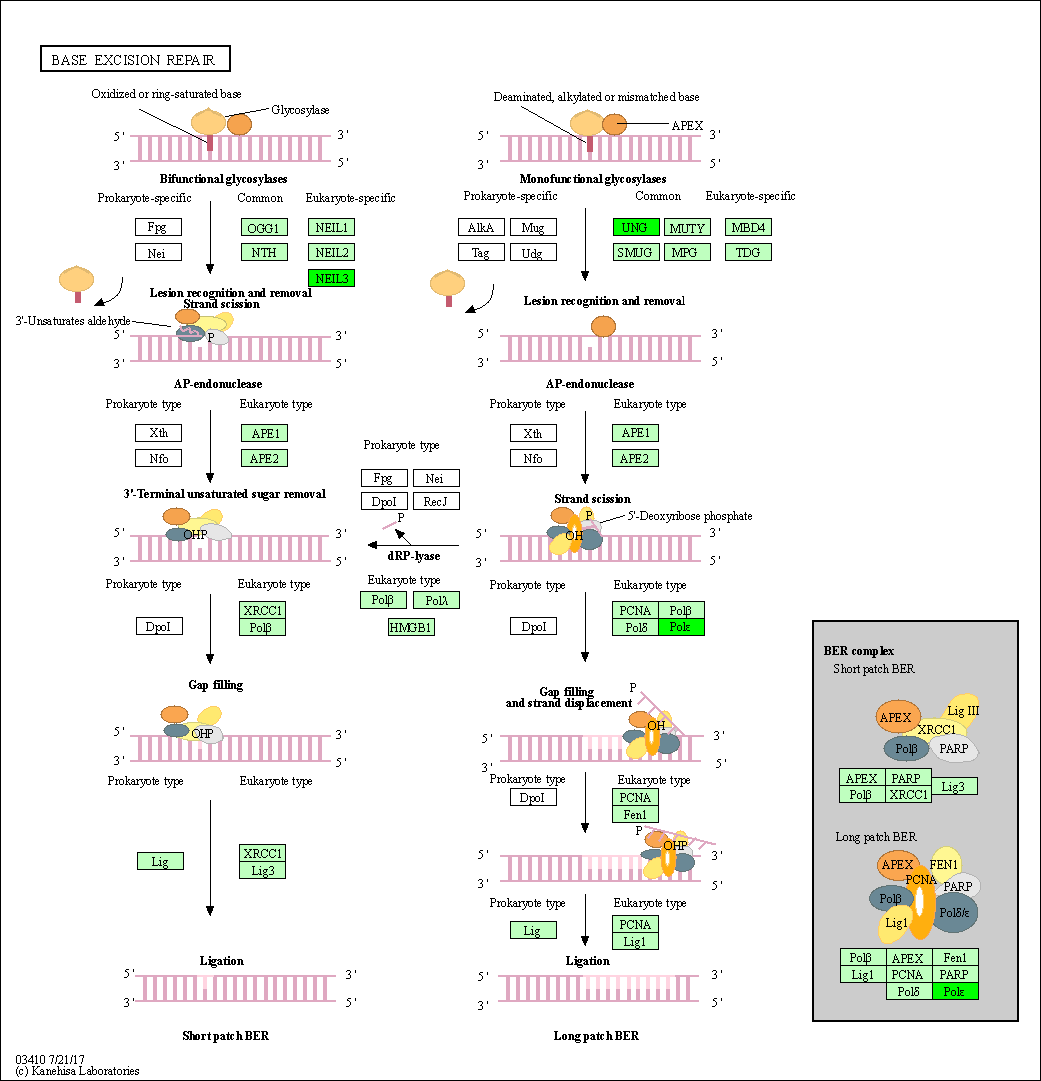

Supplement: Supplementary file 1 [file molecules-28-01606-s001.zip › raw data/KEGG/IL-1b_vs_N/DEG_pathway/mmu03410.png]

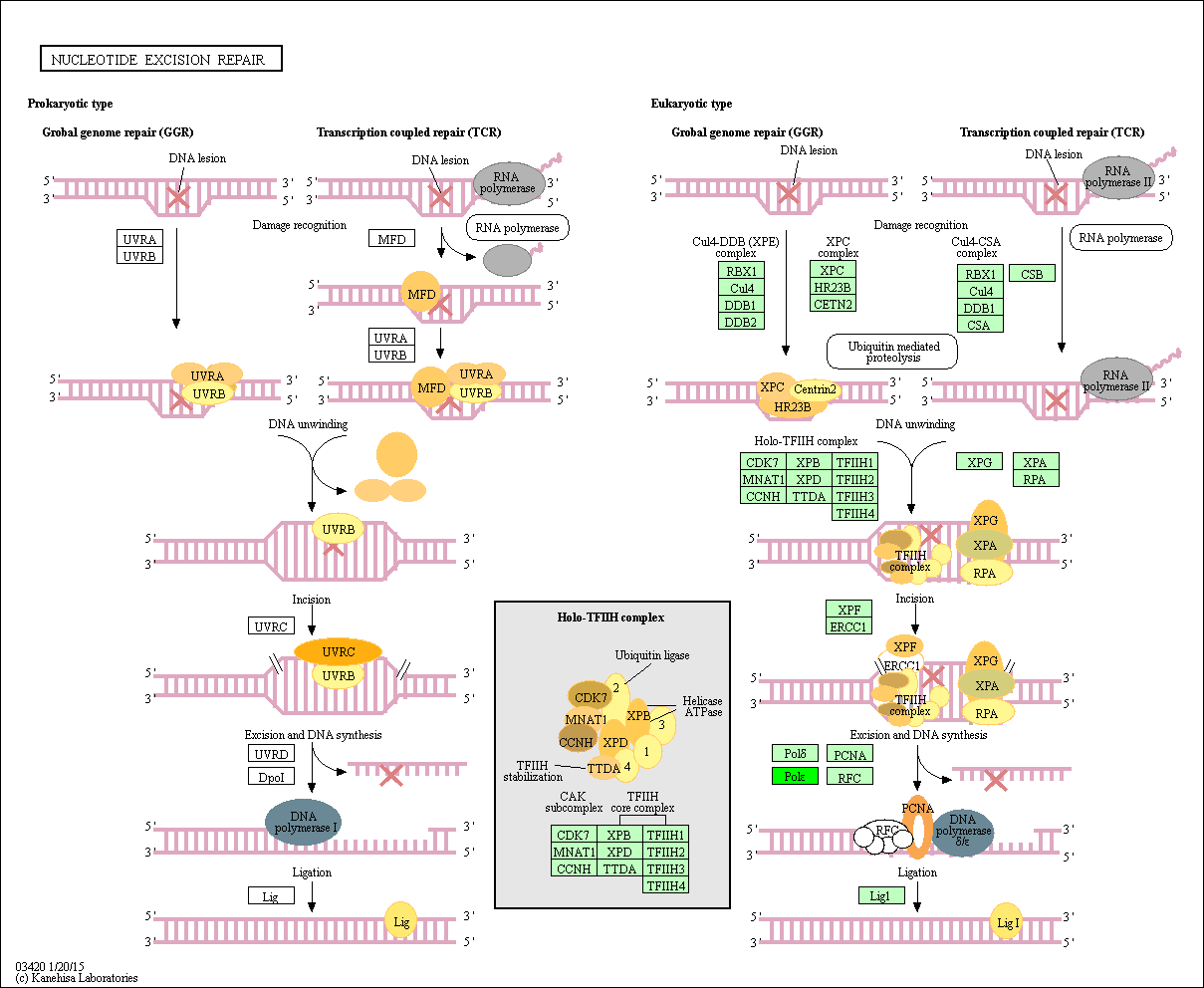

Supplement: Supplementary file 1 [file molecules-28-01606-s001.zip › raw data/KEGG/IL-1b_vs_N/DEG_pathway/mmu03420.png]
